# Synthesis and structure–activity relationships of indole-3-butyric acid-based hydrazones: predicting their antibacterial and antioxidant potential through integrated experimental, molecular docking and DFT studies

**DOI:** 10.1039/d6ra01763k

**Published:** 2026-06-03

**Authors:** Mudasara Azam, Talha Mashhood, Shehar Bano, Muhammad Ibrahim, Sehar Nadeem, Humaira Zulfiqar, Nora Hamad Al-Shaalan, Sarah Alharthi, Mohammed A. Amin, Muhammad Usman Khan

**Affiliations:** a Department of Applied Chemistry, Government College University Faisalabad Faisalabad-38000 Pakistan ibrahim@gcuf.edu.pk; b Department of Chemistry, University of Okara Okara-56300 Pakistan usman.chemistry@gmail.com usmankhan@uo.edu.pk; c Department of Chemistry, COMSATS University Islamabad Abbottabad Pakistan; d Department of Chemistry, College of Science, Princess Nourah bint Abdulrahman University P.O. Box 84428 Riyadh 11671 Saudi Arabia; e Department of Chemistry, College of Science, Taif University P.O. Box 11099 Taif 21944 Saudi Arabia; f Center of Advanced Research in Science and Technology, Taif University P.O. Box 11099 Taif 21944 Saudi Arabia; g Chemistry Department, Faculty of Science, Ain Shams University Abbassia Cairo 11566 Egypt

## Abstract

Indole-based hydrazone derivatives are a valuable group of molecules owing to their adjustable electronic structures, strong donor–acceptor properties, and broad biological applications in antioxidants, antibacterials, and optoelectronics. In the current work, indole 3-butyric acid (IBH) derivatives of hydrazones (DHIBH, PIBH, NIBH, MBIBH, 4-MBIBH, ICIBH, 4-PCIBH, and TCIBH) were prepared using a multi-step method with aldehydes or ketones substituted to form the hydrazide group. Melting point analysis and FTIR, UV-visible and NMR spectroscopy confirmed the structures of the hydrazine derivatives. The evaluation of biological activity showed trends in activity. DHIBH outperformed all other compounds synthesized in terms of antibacterial screening against *Escherichia coli* and *Staphylococcus aureus*, with maximum zones of inhibition of 18.3 mm and 19.3 mm, respectively, and an MIC of 2.5 mg mL^−1^. Meanwhile, ICIBH and 4-PCIBH exhibited selective performance against the Gram-positive strain at a dosage of 2.5 mg mL^−1^, while the antioxidant activity was observed using the DPPH assay, with DHIBH being the most effective radical scavenger with an IC_50_ of 44.51 ± 7.46 µL mL^−1^. The findings of the experiment were justified by extensive DFT computations. There was good agreement between the experimental vibrational frequencies and the calculated vibrational frequencies, which verified the structural stability and functional group integrity of the structure using comparative IR analysis. Frontier molecular orbital analysis has shown that the HOMO–LUMO energy gaps are substituent-dependent, which shows variable chemical reactivity and charge-transfer capacity. UV-vis analysis favors the predominant π → π and n → π transitions with the bathochromic effects of extended conjugation and electron-withdrawing substituents, respectively. Nonlinear optical (NLO) computations proved an increase in the dipole moment, polarizability, and first hyperpolarizability of a few derivatives, which denoted their multifunctional behavior. Molecular electrostatic potential, global reactivity descriptors, and NBO analyses were further used to provide greater insights into and confirm the presence of efficient intramolecular charge transfer. A strong correlation between molecular docking with DNA gyrase B (PDB ID: 6F86) and antioxidant-related protein (PDB ID: 1HD2) was observed against experimental antibacterial and antioxidant data, especially with DHIBH and NIBH. The ADMET results confirmed the favorable drug-like and pharmacokinetic properties of all the compounds. In general, these findings indicate that indole-based hydrazones are promising lead structures that should be further optimized structurally and biologically prior to the determination of their therapeutic potential.

## Abstract 2

The synthesis and DFT-guided analysis of indole-3-butyric acid hydrazones reveal DHIBH as a potent antibacterial and antioxidant, with docking, NLO, and ADMET studies supporting its multifunctional potential.

## Graphical abstract

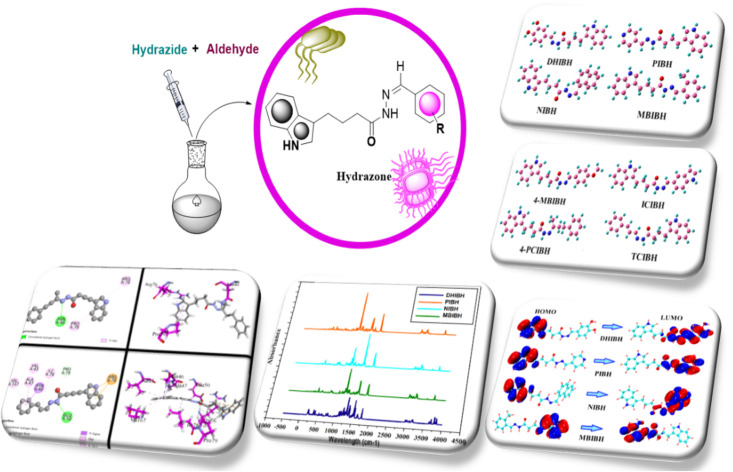

## 1 Introduction

**Fig. 1:**
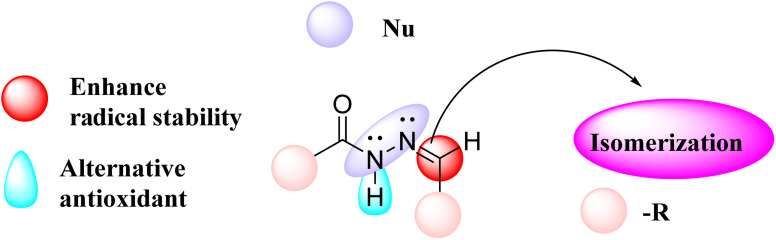
Structural assortment of the hydrazone functionality.

Nitrogen and oxygen heterocyclic frameworks are of considerable pharmaceutical significance as numerous bioactive natural products and clinically approved drugs incorporate these privileged scaffolds.^1^ Among these architectures, hydrazones featuring the characteristic –CONH–N=CH– linkage with multiple electron donor sites constitute a distinct subclass within the Schiff base family.^2^ From a synthetic standpoint, hydrazones participate as key substrates in several named transformations, notably the Shapiro, Bamford–Stevens and iodination reactions. Conversely, they function as critical intermediates in Wolff–Kishner reductions.^3^ Structurally, these compounds are obtained *via* the condensation of hydrazides with an aldehyde or ketone, yielding a conjugated framework enriched with nitrogen and oxygen heteroatoms (Fig. 1). The resulting donor–acceptor architecture facilitates hydrogen bond formation with biological macromolecules, thereby modulating receptor interactions and interfering with diverse physiological processes.^4^ Importantly, hydrazones have demonstrated therapeutic utility extending well beyond antimicrobial applications, offering viable strategies to overcome drug resistance while maintaining a favorable toxicity profile.^5^ Consequently, the design and synthesis of multi-target hydrazone derivatives, exhibiting antimicrobial,^6^ cardioprotective,^7^ anti-HIV,^8^ anti-inflammatory,^9^ antioxidant,^10^ antihypertensive, anticancer,^11^ antitubercular,^12^ antidepressant,^13^ antimalarial^14^ and anticonvulsant^15^ properties.

In this regard, hydrazones are considered an important group of organic compounds due to their various applications in medicine. Hydrazone derivatives are highly reactive because the NH functional group is acidic and has electrophilic and nucleophilic sites. We note that acidic hydrazones are subject to amido–iminol tautomerism, making them interesting bidentate ligands. They are keto preferred in the solid phase but are found as an equilibrium mixture of enol and keto species in solution. The key structural attributes responsible for the medicinal relevance of hydrazones include their ability to enhance radical stability, act as alternative antioxidants, participate in nucleophilic reactions, and undergo isomerization.

**Fig. 2:**
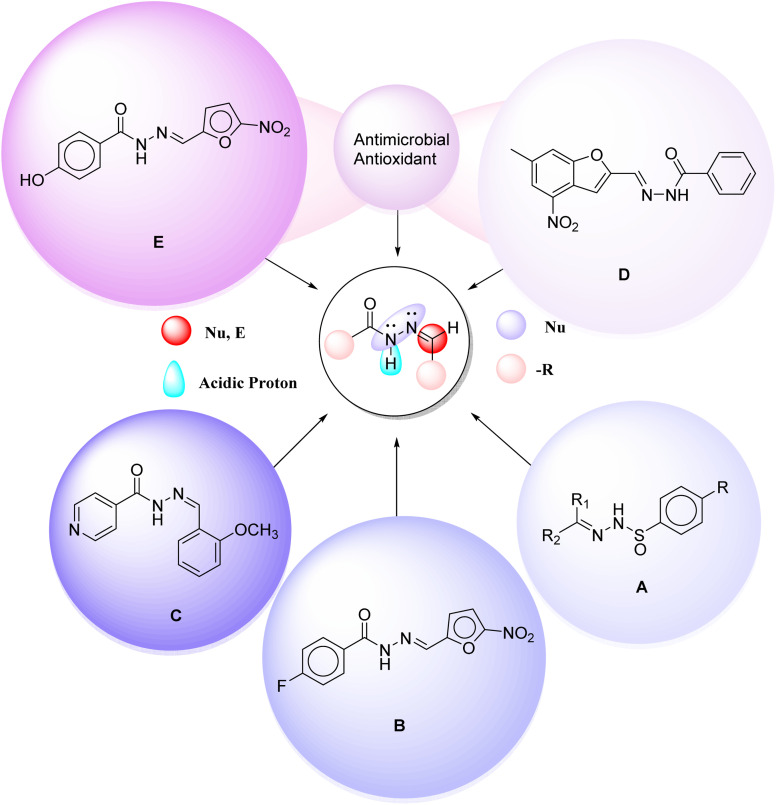
Representative hydrazone derivatives (A)–(E) with reported antimicrobial and antioxidant activities from the literature.

Hydrazones have been extensively studied for their broad spectrum of biological activities.^16^ According to Sondhi *et al.* (2006),^17^ compound A (Fig. 2) is a sufficiently stable compound that neutralizes free radicals *via* electron transfer, thereby reducing their destructive capability. This is based on an evaluation of the active characteristics of synthetic hydrazone antioxidants. Compound B (Fig. 2) was reported by Shah *et al.* (2012) as a registered antitubercular and antibacterial agent. Küçükgüzel *et al.* (2003).^18^ Tayade *et al.* (2022)^19^ synthesized compound C (Fig. 2), which exhibited the highest antioxidant activity among the synthesized compounds. Jabeen (2022)^20^ synthesized D (Fig. 2), an antibacterial drug containing a hydrazone moiety. Shah *et al.* (2012)^21^ reported that the nifuroxazide structure is used as an intestinal antiseptic compound E (Fig. 2).

**Fig. 3:**
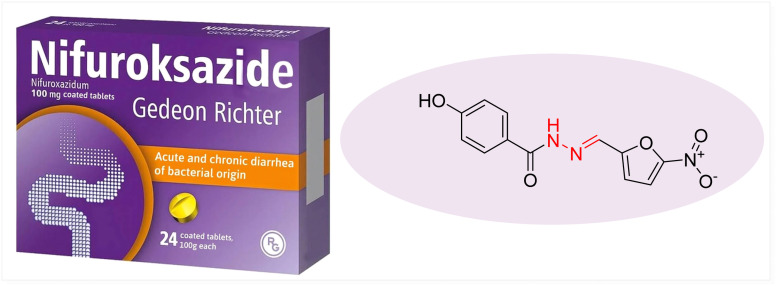
Antibacterial drug nifuroxazide.

Nifuroxazide (Fig. 3) is a well-known nitrofuran-based drug containing a hydrazone moiety that has attracted significant attention in medicinal chemistry due to its vast spectrum of antibacterial activity and low toxicity.^22^ It is used to treat acute diarrheal infections.^23^ It affects the gastrointestinal tract, with its effects retained, and does not affect the entire intestinal flora.^24^ The hydrazone functionality enhances its molecular stability, biological activity, and conjugation between the aromatic ring and nitrofuran.^25^ This conjugation enhances the enzymatic reduction of the nitro group inside bacterial cells. Hydrazone proton donor and acceptor sites interact strongly with bacterial enzymes and proteins by improving their binding ability.^26^

Despite several hydrazone analogs reported in the literature, no systematic study has been performed to synthesize, fully characterize spectroscopically, analyze by DFT, and molecularly dock IBA-derived hydrazones with dual antibacterial and antioxidant profiling.

In multidisciplinary research, computational studies of newly synthesized organic compounds have increasingly gained significance in elucidating optoelectronic characteristics, molecular stability, and atomic-level reactivity.^27–30^ Density functional theory (DFT) simulations provide reliable information on ideal geometries, electronic structures, charge distribution, and frontier molecular orbitals, which are crucial for correlating structural properties with experimental data.^31–34^ Furthermore, molecular docking studies offer molecular-level elucidation of biological activity by detailing the binding modalities, interaction patterns, and binding stability in biological targets.^35^ The current research thus proposes to synthesize and spectroscopically characterize eight new IBA-based hydrazone analogs,^36^ determine their *in vitro* antibacterial and antioxidant properties, and (3) rationalize the resultant structure–activity relationships using DFT calculations and molecular docking.

## 2 Materials and methods

Indole-3-butyric acid (Sigma-Aldrich), acetophenone (Sigma-Aldrich), 4-hydroxy acetophenone (Sigma-Aldrich), 2-hydroxybenzaldehyde (Sigma-Aldrich), 2,4,5-trimethoxybenzaldehyde (Sigma-Aldrich), 4-chloroacetophenone (Sigma-Aldrich), sulfuric acid (MERCK), hydrazine (Sigma-Aldrich), methanol (Sigma-Aldrich), Cefixime (Sigma-Aldrich), and Ciprofloxacin (MERCK).

### 2.1 General procedure

The synthesis of the hydrazones was carried out in a series of steps that began with the esterification of indole-3-butyric acid. In the next step, the hydrazide was synthesized by refluxing the ester with hydrazine hydrate for 4 h using methanol as a solvent. All the reagents used were of analytical purity, and the solvents were used without additional purification. The transformation process was evaluated by thin-layer chromatography using silica gel plates (0.25 mm) and visual observation. NMR spectra were obtained using a Bruker Avance 400 MHz spectrometer in DMSO-d_6_, and Fourier-transform infrared spectra were obtained using a Shimadzu IR Prestige-21 instrument. Absorption analysis was carried out using a Shimadzu UV-vis spectrophotometer (model UV-5100) with a spectral window of 190–1000 nm, using a deuterium lamp as a source of short wavelength radiation and a tungsten filament as the source of long wavelength radiation.

### 2.2 Technique for the synthesis of indole-3-butyric acid-based hydrazones (DHIBH, PIBH, NIBH, MBIBH, 4-MBIBH, ICIBH, 4-PCIBH and TCIBH)

In methanol, a solution of indole-3-butyric acid hydrazide (0.47 mmol) and substituted aromatic aldehydes (0.47 mmol) was allowed to reflux for four hours. At the end of the reaction, which was followed by the use of thin-layer chromatography, the reaction mixture was concentrated using a rotary evaporator. The crude material was then subjected to routine workup, and the final products were purified using column chromatography.

#### 2.2.1 *N′*-(3,4-Dihydroxybenzylidene)-4-(1*H*-indol-3-yl)butanehydrazide (DHIBH)

M.P: 131–132 °C, yield: 72%.

IR *ν*_max_ (cm^−1^): 3373 (N–H, amidic) str, 3045 (C–H, sp^2^) str, 2914–2868 (C–H, sp^3^) str, 1664 (–C=O, amidic), 1533 (–C=N, iminic), 1372 (C–N).

UV *λ*_max_ = 368 nm corresponds to the π → π* and n → π* transitions of the conjugated hydrazone (–C=N–NH–) system, indicating extended conjugation in the molecule.

^1^H-NMR (400 MHz, DMSO) *δ* 10.96 (s, 1H, N–H^15^, amide), 10.74 (s, 1H, N–H^1^), 9.30 (s, 1H, OH^25^), 9.24 (s, 1H, OH^24^), 7.94 (s, 1H, Ar–H^5^), 7.79 (s, 1H, Ar–H^26^), 7.52 (dd, *J* = 7.9, 4.7 Hz, 1H, Ar–H^8^), 7.33 (d, *J* = 8.3 Hz, 1H, Ar–H^23^), 7.18–7.16 (m, 1H, Ar–H^19^), 7.13–7.01 (m, 2H, Ar–H^2,7^), 6.96 (td, *J* = 7.4, 4.1 Hz, 1H, Ar–H^6^), 6.75 (d, *J* = 8.9 Hz, 1H, Ar–H^22^), 2.79–2.62 (m, 3H, H^13″,12^), 2.22 (t, *J* = 7.4 Hz, 1H, H^13′^), 1.94 (td, *J* = 7.7, 4.3 Hz, 2H, H^12^).

^13^C-NMR (101 MHz, DMSO) *δ* 174.42, 168.69, 147.92, 146.06, 143.48, 136.78, 126.29, 122.77, 122.66, 121.29, 120.78, 120.12, 118.81, 118.57, 116.07, 115.96, 113.08, 111.79, 40.58, 40.37, 40.16, 39.96, 39.75, 39.54, 39.33, 32.41, 26.37, 24.98.

#### 2.2.2 (*E*)-4-(1*H*-Indol-3-yl)-*N′*-(pyridin-4-ylmethylene)butanehydrazide (PIBH)

M.P: 127–129 °C, yield: 70%.

IR *ν*_max_ (cm^−1^): 3343 (N–H, amidic) str, 3055 (C–H, sp^2^) str, 2914–2868 (C–H, sp^3^) str, 1684 (–C=O, amidic), 1531 (–C=N, iminic), 1361 (C–N).

UV *λ*_max_ = 335 nm corresponds to the π → π* and n → π* transitions of the conjugated hydrazone (–C=N–NH–) system, indicating extended conjugation in the molecule.

^1^H-NMR (400 MHz, DMSO) *δ* 11.48 (s, 1H, N–H^1^), 10.78 (s, 1H, N–H^15^, amide), 8.58–8.56 (m, 2H, Ar–H^17,21^), 7.91 (s, 1H, Ar–H^23^), 7.52 (dd, *J* = 7.9, 3.4 Hz, 1H, Ar–H^8^), 7.48–7.42 (m, 2H, Ar–H^18,20^), 7.34 (dd, *J* = 8.2, 4.3 Hz, 1H, Ar–H^5^), 7.13 (d, *J* = 2.3 Hz, 1H, Ar–H^2^), 7.06 (t, *J* = 7.6 Hz, 1H, Ar–H^7^), 7.01–6.91 (m, 1H, Ar–H^6^), 2.81–2.66 (m, 3H, H^13″,11^), 2.30 (t, *J* = 7.4 Hz, 1H, H^13′^), 1.96 (dt, *J* = 14.9, 5.5 Hz, 2H, H^12^).

^13^C-NMR (101 MHz, DMSO) *δ* 169.56, 150.65, 150.62, 143.71, 140.25, 136.78, 127.70, 122.84, 121.29, 120.99, 118.79, 118.59, 114.55, 111.81, 40.59, 40.39, 40.18, 39.97, 39.76, 39.55, 39.34, 32.39, 25.84, 24.82.

#### 2.2.3 (*Z*)-4-(1*H*-Indol-3-yl)-*N′*-(naphthalen-1-ylmethylene)butanehydrazide (NIBH)

M.P: 132–134 °C, yield: 68%.

IR *ν*_max_ (cm^−1^): 3366 (N–H, amidic) str, 3054 (C–H, sp^2^) str, 2924–2863 (C–H, sp^3^) str, 1656 (–C=O, amidic), 1548 (–C=N, iminic), 1372 (C–N).

UV *λ*_max_ = 347 nm corresponds to the π → π* and n → π* transitions of the conjugated hydrazone (–C=N–NH–) system, indicating extended conjugation in the molecule.

^1^H-NMR (400 MHz, DMSO) *δ* 11.27 (s, 1H, N–H^15^, amide), 10.78 (s, 1H, N–H^1^), 8.60 (d, *J* = 8.0 Hz, 1H, Ar–H^26^), 7.98 (dq, *J* = 6.3, 3.0 Hz, 2H, Ar–H^19,24^), 7.86 (d, *J* = 7.2 Hz, 1H, Ar–H^22^), 7.78 (d, *J* = 7.3 Hz, 1H, Ar–H^8^), 7.68–7.51 (m, 4H, Ar–H^5,20,21,25^), 7.34 (dd, *J* = 8.2, 4.3 Hz, 1H, Ar–H^2^), 7.15 (s, 1H, Ar–H^17^), 7.06 (q, *J* = 7.1 Hz, 1H, Ar–H^7^), 6.95 (dt, *J* = 15.1, 7.4 Hz, 1H, Ar–H^6^), 2.78 (dt, *J* = 15.8, 7.5 Hz, 3H, H^13″,11^), 2.33 (t, *J* = 7.4 Hz, 1H, H^13′^), 2.01 (td, *J* = 7.7, 3.7 Hz, 2H, H^12^).

^13^C-NMR (101 MHz, DMSO) *δ* 169.14, 142.60, 136.80, 133.98, 130.79, 130.51, 129.28, 129.21, 127.68, 127.36, 126.67, 126.03, 125.98, 124.13, 122.78, 121.27, 118.81, 118.60, 118.57, 111.81, 32.63, 25.72, 24.98.

#### 2.2.4 (*E*)-4-(1*H*-Indol-3-yl)-*N′*-(2-methylbenzylidene)butanehydrazide (MBIBH)

M.P: 140–141.5 °C, yield: 78%.

IR *ν*_max_ (cm^−1^): 3365 (N–H, amidic) str, 3042 (C–H, sp^2^) str, 2915–2862 (C–H, sp^3^) str, 1655 (–C=O, amidic), 1556 (–C=N, iminic), 1377 (C–N).

UV *λ*_max_ = 304 nm corresponds to the π → π* and n → π* transitions of the conjugated hydrazone (–C=N–NH–) system, indicating extended conjugation in the molecule.

^1^H-NMR (400 MHz, DMSO) *δ* 11.13 (s, 1H, N–H^24^, amide), 10.77 (s, 1H, N–H^23^), 8.23 (s, 1H, Ar–H^15^), 7.63–7.56 (m, 1H, Ar–H^2^), 7.52 (d, *J* = 7.8 Hz, 1H, Ar–H^4^), 7.37–7.17 (m, 4H, Ar–H^1,18,19,20^), 7.12 (dd, *J* = 5.9, 2.3 Hz, 1H, Ar–H^6^), 7.09–7.01 (m, 1H, Ar–H^7^), 6.96 (q, *J* = 7.6 Hz, 1H, Ar–H^5^), 2.80–2.63 (m, 4H, Ar–H^10″,12^), 2.38 (d, *J* = 10.7 Hz, 3H, H^22^), 2.26 (t, *J* = 7.4 Hz, 1H, H^10′^), 1.95 (p, *J* = 7.4 Hz, 2H, H^12^).

^13^C-NMR (101 MHz, DMSO) *δ* 168.98, 144.69, 141.97, 136.78, 132.70, 131.34, 129.67, 127.68, 126.61, 126.55, 122.75, 121.26, 118.78, 118.55, 114.63, 111.79, 40.60, 40.39, 40.19, 39.98, 39.77, 39.56, 39.35, 32.51, 25.79, 24.92, 19.84.

#### 2.2.5 (*E*)-4-(1*H*-Indol-3-yl)-*N′*-(4-methoxybenzylidene)butanehydrazide (4-MBIBH)

M.P: 116–118 °C, yield: 83%.

IR *ν*_max_ (cm^−1^): 3299–3230 (–N–H, amidic), 3060 (Ar–H) str, 2938–2847 (C–H, sp^3^) str, 1652 (–C=O, amidic)str, 1606 (C=C)str, 1542 (–C=N, iminic)str, 1246 (C–N)str.

UV *λ*_max_ = 389 nm corresponds to the π → π* and n → π* transitions of the conjugated hydrazone (–C=N–NH–) system, indicating extended conjugation in the molecule.

^1^H-NMR (400 MHz, DMSO) *δ* 11.05 (s, 1H, N–H^1^), 10.75 (s, 1H, N–H^15^, amide), 7.88 (s, 1H, Ar–H^25^), 7.64–7.56 (m, 1H, Ar–H^5^), 7.55–7.43 (m, 2H, Ar–H^18,22^), 7.33 (dq, *J* = 7.9, 1.1 Hz, 1H, Ar–H^8^), 7.11 (d, *J* = 2.3 Hz, 1H, Ar–H^2^), 7.05 (ddd, *J* = 8.1, 7.0, 1.2 Hz, 1H, Ar–H^7^), 7.01–6.91 (m, 3H, Ar–H^6,19,21^), 3.78 (d, *J* = 1.5 Hz, 3H, CH_3_^24^), 2.70 (ddd, *J* = 31.1, 15.1, 7.5 Hz, 3H, H^11″,13^), 2.23 (t, *J* = 7.4 Hz, 1H, H^11′^), 1.93 (p, *J* = 7.5 Hz, 2H, H^12^).

^13^C-NMR (101 MHz, DMSO) *δ* 168.36, 160.38, 142.12, 136.29, 128.46, 128.07, 127.21, 126.92, 122.27, 120.77, 118.31, 118.09, 118.07, 114.23, 111.30, 55.24, 32.02, 25.35, 24.43.

#### 2.2.6 (*E*)-*N′*-((1*H*-Indol-5-yl)methylene)-4-(1*H*-indol-3-yl)butanehydrazide (ICIBH)

M.P: 126–127 °C, yield: 76%.

IR *ν*_max_ (cm^−1^): 3368 (–N–H, amidic), 3036 (Ar–H) str, 2926–2865 (C–H, sp^3^) str, 1643 (–C=O, amidic)str, 1598 (C=C)str, 1557 (–C=N, iminic)str, 1284 (C–N)str.

UV *λ*_max_ = 308 nm corresponds to the π → π* and n → π* transitions of the conjugated hydrazone (–C=N–NH–) system, indicating extended conjugation in the molecule.

^1^H-NMR (500 MHz, DMSO) *δ* 11.26 (s, 1H, Ar–H^21^), 10.99 (s, 1H, Ar–H^17^), 10.76 (s, 1H, Ar–H^15^, amide), 8.03 (s, 1H, Ar–H^26^), 7.70 (s, 1H, Ar–H^18^), 7.53 (t, *J* = 9.5 Hz, 2H, Ar–H^5,8^), 7.42 (d, *J* = 9.8 Hz, 1H, Ar–H^24^), 7.37 (s, 1H, Ar–H^23^), 7.33 (d, *J* = 7.8 Hz, 1H, Ar–H^2^), 7.13 (s, 1H, Ar–H^21^), 7.05 (d, *J* = 7.8 Hz, 1H, Ar–H^6^), 6.98–6.92 (m, 1H, Ar–H^7^), 6.48 (s, 1H, Ar–H^19^), 2.77 (t, *J* = 7.3 Hz, 1H, H^12″^), 2.71 (d, *J* = 9.9 Hz, 2H, H^13^), 2.25 (t, *J* = 7.3 Hz, 1H, H^12^), 2.00–1.93 (m, 2H, H^11^).

^13^C-NMR (126 MHz, DMSO) *δ* 168.21, 144.35, 136.68, 136.30, 127.56, 127.23, 126.26, 125.52, 122.27, 120.75, 120.33, 118.95, 118.32, 118.05, 111.92, 111.88, 111.29, 101.82, 32.04, 25.32, 24.48.

#### 2.2.7 (*E*)-4-(1*H*-Indol-3-yl)-*N′*-(4-phenylbutan-2-ylidene)butanehydrazide (4-PCIBH)

M.P: 137–138 °C, yield: 64%.

IR *ν*_max_ (cm^−1^): 3363 (–N–H, amidic)str, 3036 (Ar–H)str, 2946–2865 (C–H, sp^3^)str, 1646 (–C=O, amidic)str, 1599 (C=C)str, 1565 (–C=N, iminic)str, 1278 (C–N)str.

UV *λ*_max_ = 309 nm corresponds to the π → π* and n → π* transitions of the conjugated hydrazone (–C=N–NH–) system, indicating extended conjugation in the molecule.

^1^H-NMR (500 MHz, DMSO) *δ* 10.74 (s, 1H, N–H^15^, amide), 9.91 (s, 1H, N–H^1^), 7.52–7.48 (m, 1H, Ar–H^5^), 7.29–7.21 (m, 5H, Ar–H^22,23,24,25,26^), 7.18–7.13 (m, 2H, Ar–H^7,8^), 7.10–7.04 (m, 2H, Ar–H^2,6^), 2.77 (dt, *J* = 12.7, 7.7 Hz, 2H, H^13^), 2.68 (d, *J* = 7.5 Hz, 2H, H^12^), 2.53 (d, *J* = 7.5 Hz, 1H, H^11″^), 2.47–2.43 (m, 1H, H^11′^), 1.93–1.83 (m, 4H, H^12,18^), 1.82 (s, 3H, CH_3_^19^).

^13^C-NMR (126 MHz, DMSO) *δ* 168.57, 151.19, 141.37, 136.28, 128.24, 128.13, 127.18, 125.79, 125.69, 122.14, 120.77, 120.74, 118.25, 118.01, 111.27, 33.72, 32.19, 31.54, 26.03, 24.43, 15.99.

#### 2.2.8 4-(1*H*-Indol-3-yl)-*N′*-((1*E*,2*E*)-3-phenylallylidene)butanehydrazide (TCIBH)

M.P: 114–116 °C, yield: 62%.

IR *ν*_max_ (cm^−1^): 3305 (–N–H, amidic)str, 3160 (C–H, sp^2^)str, 3041 (Ar–H)str, 2921–2865 (C–H, sp^3^)str, 1643 (–C=O, amidic)str, 1544 (C=C)str, 1486 (–C=N, iminic)str, 1202 (C–N)str.

UV *λ*_max_ = 332 nm corresponds to the π → π* and n → π* transitions of the conjugated hydrazone (–C=N–NH–) system, indicating extended conjugation in the molecule.

^1^H-NMR (400 MHz, DMSO) *δ* 11.12 (s, 1H, N–H^25^), 10.76 (s, 1H, N–H^1^), 7.79 (d, *J* = 8.4 Hz, 1H, Ar–H^5^), 7.58 (dt, *J* = 8.2, 2.5 Hz, 2H, Ar–H^20,24^), 7.52 (dd, *J* = 7.9, 3.4 Hz, 1H, Ar–H^16^), 7.40–7.27 (m, 4H, Ar–H^6,8,18,22^), 7.12 (d, *J* = 2.3 Hz, 1H, Ar–H^2^), 7.06 (t, *J* = 7.5 Hz, 1H, Ar–H^7^), 7.00–6.82 (m, 3H, Ar–H^21,23,27^), 2.72 (q, *J* = 7.9 Hz, 2H, H^13^), 2.60 (t, *J* = 7.4 Hz, 1H, H^11″^), 2.24 (t, *J* = 7.4 Hz, 1H, H^11′^), 1.94 (p, *J* = 7.4 Hz, 2H, H^12^).

^13^C-NMR (101 MHz, DMSO) *δ* 168.98, 138.54, 136.80, 136.41, 129.28, 129.24, 129.18, 129.11, 127.64, 127.44, 122.72, 121.29, 118.82, 118.78, 118.55, 111.80, 32.13, 26.25, 24.87.

### 2.3 *In vitro* antioxidant activity

The potential antioxidant capabilities of the derivatized compounds (DHIBH, PIBH, NIBH, MBIBH, 4-MBIBH, ICIBH, 4-PCIBH, and TCIBH) were assessed by preparing stock solutions of the test samples in DMSO at a concentration of 10 mg mL^−1^. A DPPH solution comprising 9.2 mg of 2,2-diphenyl-1-picrylhydrazyl (DPPH) dissolved in 100 mL of methanol was also prepared. Subsequently, different concentrations of each test sample (12.5, 25, 50, and 100 µL) were combined with DPPH solution, maintaining a final volume of 1 mL to obtain final concentrations of 0.125, 0.25, 0.5 and 1 mg mL^−1^, respectively. Ascorbic acid was used as the standard, while the DPPH solution served as the control (*A*_blank_). The reaction mixtures were incubated in the dark at 37 °C for 60 minutes, and the absorbance of the samples was assessed at 517 nm using a UV-visible spectrophotometer. The proportion of inhibition or scavenging activity was assessed by quantifying the discolouration of the DPPH solution. The free radical scavenging activity was quantified as percentage inhibition and calculated using the following equation:Inhibition (%) = [(*A*_blank_ − *A*_sample_)/*A*_blank_] × 100,where *A*_blank_ represents the absorbance of the DPPH solution and *A*_sample_ represents the absorbance of the test compound. The calculated inhibition values indicate the DPPH radical scavenging efficiency of each compound.

### 2.4 Antibacterial activity of synthesized compounds

The agar well diffusion assay was utilized to assess the antibacterial effectiveness of the synthetic compounds DHIBH, PIBH, NIBH, MBIBH, 4-MBIBH, ICIBH, 4-PCIBH and TCIBH against pathogenic strains *Escherichia coli* ATCC 25922 and *Staphylococcus aureus* ATCC 25923 at a concentration of 10^8^ CFU per mL. Wells of 8 mm were created in Luria Bertani (LB) agar plates by a sterilized cork borer. Pathogenic bacterial strains were disseminated across the plates using sterilized cotton swabs. After dissolving the compounds (DHIBH, PIBH, NIBH, MBIBH, 4-MBIBH, ICIBH, 4-PCIBH, and TCIBH) in 10 mg mL^−1^ of DMSO, 80 µL of each compound was added to the well. Ciprofloxacin functioned as a positive control, while the solvent (DMSO) served as the negative control. The plates were incubated at 37 °C for 24 hours. After 24 hours, the inhibition zones around the wells were measured in millimeters with a zone meter.^37^

### 2.5 Minimum inhibitory concentration

The minimum inhibitory concentration (MIC) of the synthesized compounds was determined by the broth microdilution assay previously described by Swebocki *et al.*^38^ as per the Clinical and Laboratory Standards Institute^39^ procedures for antimicrobial susceptibility testing, with few minor modifications. A stock solution of each compound was prepared in DMSO at a concentration of 10 mg mL^−1^. Initially, 90 µL of sterile Luria Bertani (LB) broth was added to each well of the microtiter plate. Then, 90 µL of stock solution was added to the first well of the plate. A two-fold dilution was accomplished by shifting 90 µL from the first well to subsequent wells. The bacterial suspension was diluted to achieve 0.5 McFarland at an optical density (OD) of 0.08 at 600 nm. In each well of the microtiter plate, except for the blank, 10 µL of standardized inoculum of reference strains (*E. coli* and *S. aureus*) was introduced. LB broth served as the negative control and positive control wells that contained LB with bacterial suspension. Then, all the plates were incubated at 37 °C for 24 h. The MIC was calculated by comparing each well with the negative and positive controls.

### 2.6 *In silico* molecular docking

*In silico* docking studies of synthesized analogues were carried out according to the procedure reported by Blessy and Sharmila,^48^ with minor modifications. The synthesized ligands (DHIBH, PIBH, NIBH, MBIBH, 4-MBIBH, ICIBH, 4-PCIBH, and TCIBH) were found to interact with specific biological targets of *Escherichia coli* DNA gyrase B (PDB ID: 6F86) to exhibit antibacterial activity and antioxidant-related proteins (PDB ID: 1HD2) using AutoDock Tool 1.5.7.^40^ Initial drawing of the chemical structures of the resulting compounds was performed with the ChemOffice Suite (ChemDraw 16.0) in an appropriate two-dimensional orientation. Thereafter, ChemBio3D was used to energy-minimize the molecular geometries, and optimized geometries were used as inputs for the ligands in the docking simulations. The crystallographic structures of the target proteins were obtained from the Protein Data Bank (PDB). Protein preparation was performed using conventional procedures, which included the elimination of co-crystallized ligands, the choice of water molecules, and cofactors. The ready protein structures were subjected to AutoPrep in AutoDock Tool 1.5.7, and all crucial residues were retained on the target proteins. The parameters of the docking grids were specified through the graphical user interface of AutoDock, and the grid box was placed in such a way that it covered the active site region of the macromolecule. To find the most preferred interaction conformations between the ligand and the protein, docking simulations were performed using the AutoDock Tool 1.5.7 algorithm. A maximum of nine conformers were produced in each case, with the lowest binding free energy chosen for further analysis. The resulting ligand–protein interactions were studied and visualized by Discovery Studio, in which the ligands were presented in different colors, and the hydrogen bonds and interacting amino acid residues were indicated with ball-and-stick models.^41^

The robustness of the adopted docking methodology was verified *via* a self-docking validation step. Native co-crystallized ligands were obtained from crystal structures of human peroxiredoxin 5 (PDB ID: 1HD2) and *E. coli* gyrase B; each was independently re-docked into its corresponding active sites under identical grid and scoring parameters. The accuracy of the docking method was evaluated by calculating the RMSD values between the experimentally determined binding pose and the re-docked conformation. The obtained RMSD values ≤ 2.0 Å remained reproducible and confirmed that the docking algorithm reliably recapitulated the experimentally resolved ligand orientations within the catalytic pockets.

### 2.7 ADMET prediction

The pharmacokinetic properties, encompassing absorption, distribution, metabolism, and excretion (ADME), of all synthesized compounds were evaluated using the SwissADME online tool (https://www.swissadme.ch/). The SMILES notation of each compound was used as an input to predict key parameters, such as drug-likeness, lipophilicity, solubility, and gastrointestinal absorption.

### 2.8 Density functional theory (DFT) calculation

DFT computation of DHIBH, PIBH, NIBH, MBIBH, 4-MBIBH, ICIBH, 4-PCIBH and TCIBH was carried out with Gaussian 09 (ref. 42) at the B3LYP/6-311+G(d,p) theory level. All molecular geometries were optimized completely, and the vibrational frequencies were calculated to give no imaginary frequencies, which was the true indication of minima on the potential energy surface. Frontier molecular orbital (FMO), natural bond orbital (NBO), molecular electrostatic potential (MEP), global reactivity descriptors, and nonlinear optical (NLO) properties were considered using optimized geometries at the same level of theory. The absorption spectra in the UV-visible region were simulated by performing time-dependent DFT (TD-DFT) calculations in the gas phase. Multiwfn 3.7 (ref. 43) was used to carry out electron localization function (ELF) and localized orbital locator (LOL) analyses. GaussView 5.0,^44^ GaussSum,^45^ ChemCraft^46^ and Avogadro^47^ software were used to visualize molecular structures, orbitals, spectra and topological maps.

## 3 Results and discussion

**Scheme 1:**
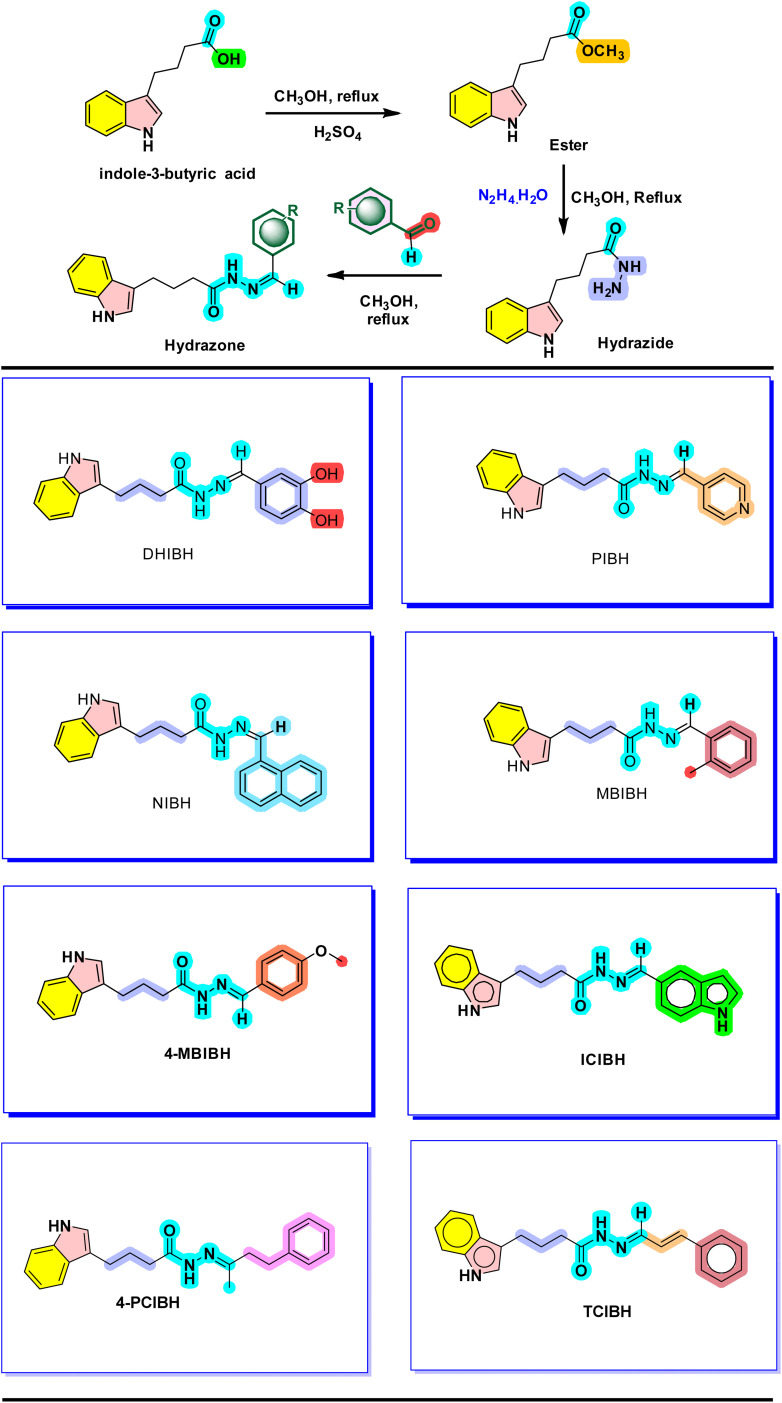
Synthetic route for the preparation of hydrazone derivatives from the naturally occurring indole-3-butyric acid.

The first step involved the transformation of indole-3-butyric acid into its ester using methanol as a solvent and a reagent in the presence of two drops of concentrated H_2_SO_4_ as a catalyst (dehydrating agent) after 4 hours of stirring. Subsequently, the reaction of ester with hydrazine monohydrate in the presence of methanol solvent was performed, thus enabling the reaction to produce indole-3-butyric acid hydrazide. Finally, substituted benzaldehydes were reacted with the hydrazide in methanol for 4 hours under reflux conditions to form the desired hydrazones (Scheme 1).

The synthesis of the hydrazones was analyzed using various spectroscopic methods. The formation of the hydrazone linkage (–C(O)–NH–N=CH–) of all the eight derivatives was validated through convergent spectroscopic evidence. The absence of the aldehyde C–H group in the products and the loss of the hydrazide –NH_2_ group (∼3288 cm^−1^) in the IR spectra provide the first indication of the effectiveness of the condensation. The typical N–H stretching band of the amide hydrazone (3373–3299 cm^−1^) is broad in each of the compounds, which is in line with intermolecular hydrogen bonding. The amide C=O vibration (1684–1643 cm^−1^) is greatly red-shifted compared to the esters (1735 cm^−1^), confirming conjugation with the azomethine bond. The appearance of the azomethine C=N stretching band (1571–1456 cm^−1^) is the most diagnostically evident in all derivatives and gives positive evidence of hydrazone formation. Interestingly, the C=N band changes frequency (to higher frequencies) with electron-withdrawing groups (pyridyl in PIBH, 1571 cm^−1^) compared to electron-donating groups (styryl in TCIBH, 1456 cm^−1^), indicating that the aromatic substituent can modulate the electronic properties of the amino-group. The geometry of the hydrazone bond was determined from the ^1^H-NMR spectra. The azomethine proton (–N=CH–), which appears as a singlet downfield (7.79–8.60 ppm), and the broad amide NH signal are evidence of the geometry of the hydrazone bond. The ^13^C-NMR carbonyl carbon (*δ* ∼168–174 ppm) and azomethine carbon (*δ* ∼140–151 ppm) prove the assigned structures.

### 3.1 Evaluation of the antioxidant potential of indole-3-butyric acid-based hydrazones

**Fig. 4:**
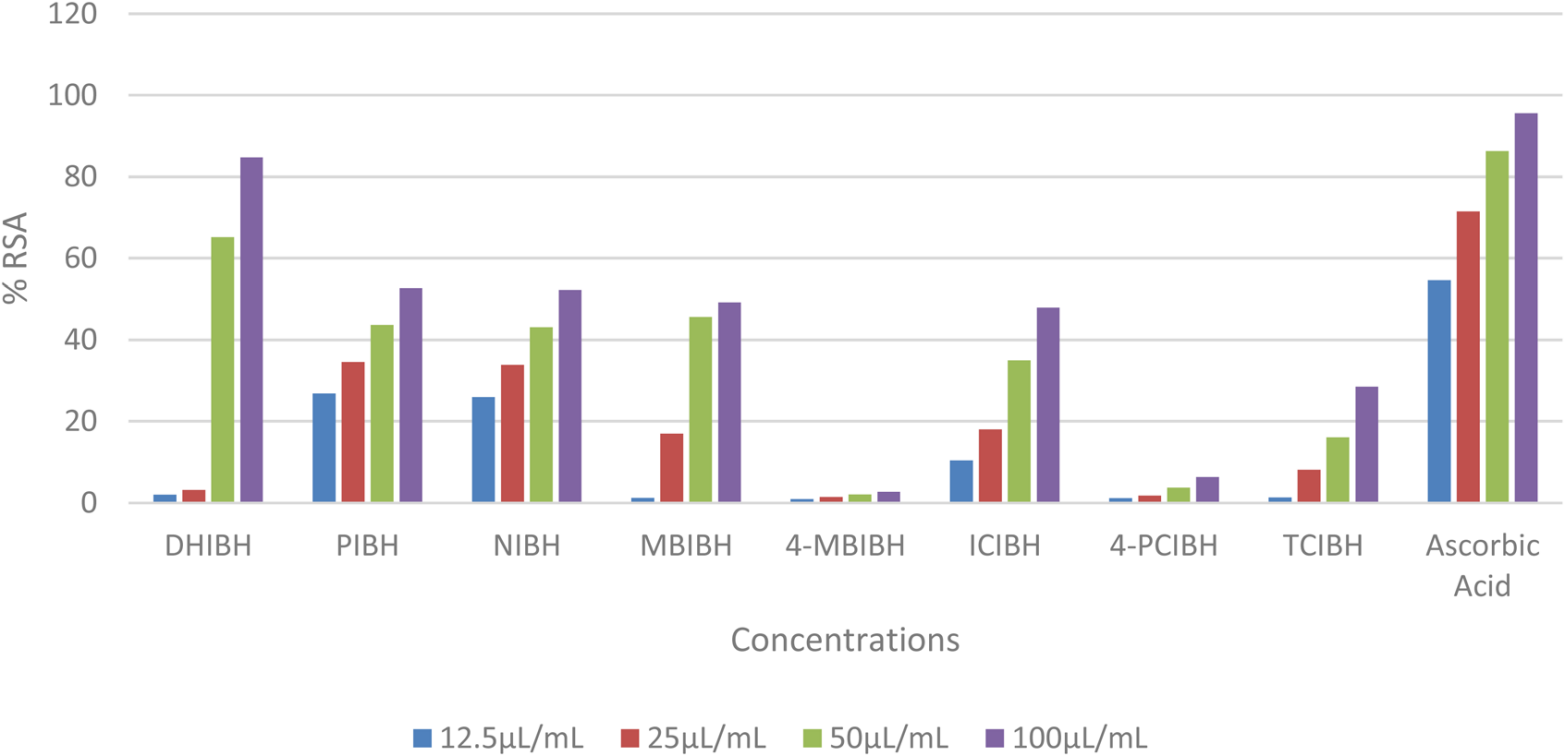
Antioxidant activity of the synthesized derivatives.

The antioxidant activity of DHIBH, PIBH, NIBH, MBIBH, 4-MBIBH, ICIBH, 4-PCIBH, and TCIBH was assessed using the DPPH assay (Table 1). The results demonstrate that DHIBH had the most significant dose-dependent increase in radical scavenging, with the most notable mean activity of 84.79% ± 0.21% at 100 µL mL^−1^. Therefore, DHIBH showed the lowest IC_50_ value (44.51 ± 7.46 µL mL^−1^) compared to PIBH (81.16 ± 0.67 µL mL^−1^) and NIBH (83.94 ± 0.46 µL mL^−1^). Other derivatives, such as MBIBH and TCIBH, did not achieve 50% inhibition in the range tested, thus resulting in ˃100 µL mL^−1^. Standard ascorbic acid was most effective with an IC_50_ of 11.07 ± 1.55 µL mL^−1^. The structural substituents of DHIBH significantly enhance their hydrogen-donating potential compared to other substitutes, as demonstrated by their high efficacy. The variation in the antioxidant capacity of derivatized substances resulted from aromatic substitution. Indole-3-butyric acid hydrazones contain conjugated electron rich structures and charge transferring properties.^48^ Hydroxyl substitution increases antioxidant properties *via* membrane affinity, electron donation and solubility.^49^ Consequently, the hierarchy of antioxidant activity for the derivatized compounds is as follows: 4-MBIBH < 4-PCIBH < TCIBH < ICIBH < MBIBH < NIBH < PIBH < DHIBH < ascorbic acid (Fig. 4).

**Table 1:** Results for the antibacterial activity of the synthesized hydrazone derivatives

Compound	Anti-oxidant activity (%) (1 mg mL^−1^)	Anti-microbial activity ZOI (mm)	Anti-microbial activity ZOI (mm)
		*E. coli*	*S. aureus*
DHIBH	84.79 ± 0.21	18.3 ± 0.88	19.3 ± 0.33
PIBH	52.75 ± 0.38	15.7 ± 0.33	10.0 ± 0.58
NIBH	52.32 ± 0.39	15.0 ± 0.58	8.7 ± 0.33
MBIBH	49.23 ± 0.16	11.3 ± 0.33	0.0
4-MBIBH	2.72 ± 0.38	8.3 ± 0.33	8.3 ± 0.33
ICIBH	47.99 ± 0.22	10.7 ± 0.38	9.7 ± 0.38
4-PCIBH	6.37 ± 0.51	13.0 ± 0.58	12.0 ± 0.58
TCIBH	28.51 ± 0.16	12.7 ± 0.88	10.7 ± 0.33
Ciprofloxacin	—	26.7 ± 0.33	26.3 ± 0.33
Ascorbic acid	95.67 ± 0.38	—	—

### 3.2 Evaluation of the anti-bacterial potential of indole-3-butyric acid-based hydrazones

**Fig. 5:**
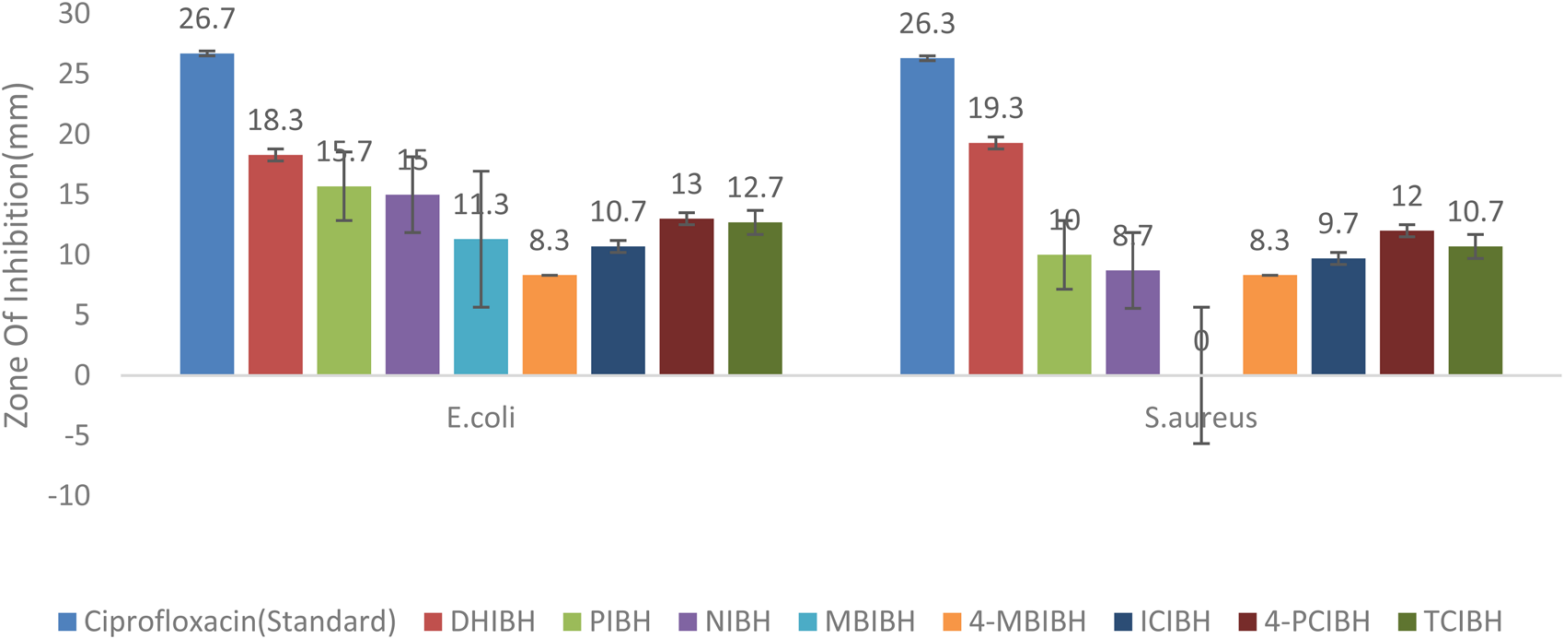
Antibacterial activity of the synthesized compounds.

The antibacterial screening results showed that the largest zone of inhibition (26.7 and 26.3 mm) was created by the reference agent ciprofloxacin^39^ against pathogenic strains of *Escherichia coli* ATCC 25922 and *Staphylococcus aureus* ATCC 25923. The consecutive synthesized indole-3-butyric acid-based hydrazone derivatives demonstrated a range of antibacterial activity, with the strongest ZOI of DHIBH, 4-MBIBH, PIBH, 4-PCIBH, and TCIBH, revealing a moderate inhibitory ability. Molecule DHIBH exhibits good activity against both pathogenic strains. The presence of hydroxyl substitution on the molecule increases its bacterial effectiveness *via* hydrogen bonding with bacterial enzymes and membranes, damaging the membrane structure and leaking the intracellular contents.^50,51^ Furthermore, ICIBH did not show activity against *S. aureus* but showed moderate activity against *E. coli* under all conditions of the experiment. Although the antibacterial activity of the synthesized compounds was lower than that of the standard antibiotic, the observed differences can be attributed to the structural differences in the hydrazone scaffold, highlighting the key role of substituent identity and position in antibacterial activity (Fig. 5). Altogether, these results support the idea of the potential of selected hydrazone analogs as a prospective scaffold to be further optimized structurally in order to increase antibacterial effectiveness. The antibacterial activity results are shown in Table 1.

### 3.3 Minimum inhibitory concentration

Minimum inhibitory concentration (MIC) screening demonstrated that a significant number of compounds exhibited moderate antibacterial activity against the Gram-negative bacteria *E. coli* within the examined concentration range (0.0024–5 mg mL^−1^). Compound DHIBH exhibited the most significant inhibitory profile, holding growth suppression at MIC concentrations of 5 and 2.5 mg mL^−1^, with calculated inhibition values of 58.09% and 40.32%, respectively. 4-PCIBH demonstrated a minimum inhibitory concentration (MIC) of 5 mg mL^−1^, indicating an average inhibition of 16.45%. In contrast, substances PIBH, NIBH, MBIBH, 4-MBIBH, ICIBH, and TCIBH did not significantly inhibit growth at the highest concentration tested, resulting in MIC values surpassing 5 mg mL^−1^. DHIBH was found to be the most potent derivative, exhibiting an MIC of 2.5 mg mL^−1^ against both strains. The high OD_600_ values recorded for substances, such as TCIBH (OD = 2.183 at 5 mg mL^−1^), indicate a notable resilience of the Gram-negative cell membrane to these chemical structures. The diverse antibacterial properties of the two strains emphasize a distinct structure–activity relationship (SAR) shaped by the characteristics of the substituents on the synthetic benzohydrazide framework. The extensive action of DHIBH indicates that its particular electronic or steric arrangement facilitates better permeability through the protein peptidoglycan layer of the Gram-positive bacteria and the lipopolysaccharide-based outer membrane of Gram-negative bacteria. Notably, numerous substances demonstrated negative inhibition values, indicating that the sample OD_600_ exceeded the control at higher concentrations. This behavior is usually attributed to the inherent chromophoric characteristics of the chemicals or solubility constraints in the aqueous broth medium, resulting in turbidity rather than the true enhancement of bacterial metabolic activity. This evidence establishes DHIBH as a principal candidate for further optimization to improve pharmacological potency and bioavailability. Ciprofloxacin displayed a minimum inhibitory concentration (MIC) of 0.078 mg mL^−1^ against *E. coli* and had remarkable antibacterial properties against *S. aureus* with an MIC of 0.0195 mg mL^−1^.

### 3.4 Molecular docking studies

**Fig. 6:**
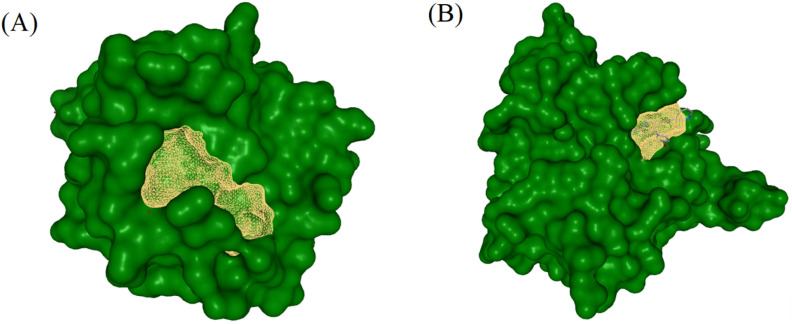
Binding pocket prediction by DoGSiteScorer for the target proteins (A) 1HD2 and (B) 6F86.

Three-dimensional crystallographic structures of the two target proteins (PDB IDs: 1HD2 and 6F86) were retrieved from the Protein Data Bank (PDB). Binding pocket identification was performed using a DoGSiteScorer. The predicted active site residues for 1HD2 (Chain A) as shown in Fig. 6(A) comprise GLU16, GLY17, PHE55, ALA59, GLN58, LEU62, LYS63, GLN68, VAL67, VAL69, THR80, VAL70, GLY82, ARG86, GLY85, ALA90, GLU91, GLY92, LYS93, ARG95, VAL94 and LEU96. The predicted active site residues for 6F86 (Chain A) as shown in Fig. 6(B) are GLU42, VAL44, VAL43, ASN46, ALA47, GLU50, ILE59, ASP49, VAL71, GLN72, ASP73, GLY75, ARG76, ILE78, GLY77, PRO79, ILE94, VAL120, MET95, ARG136, THR165, GLY164, MET166 and VAL167 (Fig. 6).

Following the completion of the docking process, strict selection criteria were applied to identify the most energetically favorable binding pose for each ligand protein. In the visual representations, ligands docked within the antioxidant protein are rendered in grey, while interacting amino acid residues are highlighted in pink. The computed binding scores for all derivatives revealed differential affinities toward the 1HD2 and 6F86 receptors, reflecting distinct interaction profiles that may govern their respective inhibitory potential.

#### 3.4.1 Antioxidant activity (target protein: PDB ID 1HD2)

The molecular docking findings with the antioxidant-related protein 1HD2 show clear variations in the binding affinity of the synthesized hydroxyl amino acid derivatives based on their structure. The most stable poses have the following docking scores: NIBH (−6.0 kcal mol^−1^), 4-PCIBH (−5.6 kcal mol^−1^), ICIBH (−5.5 kcal mol^−1^), DHIBH (−5.4 kcal mol^−1^), 4-MBIBH (−5.3 kcal mol^−1^), PIBH (−5.3 kcal mol^−1^), MBIBH (−5.3 kcal mol^−1^), and TCIBH (−5.2 kcal mol^−1^). The low RMSD values suggest that the antioxidant binding pocket accommodates its ligands in a reliable manner.

**Fig. 7:**
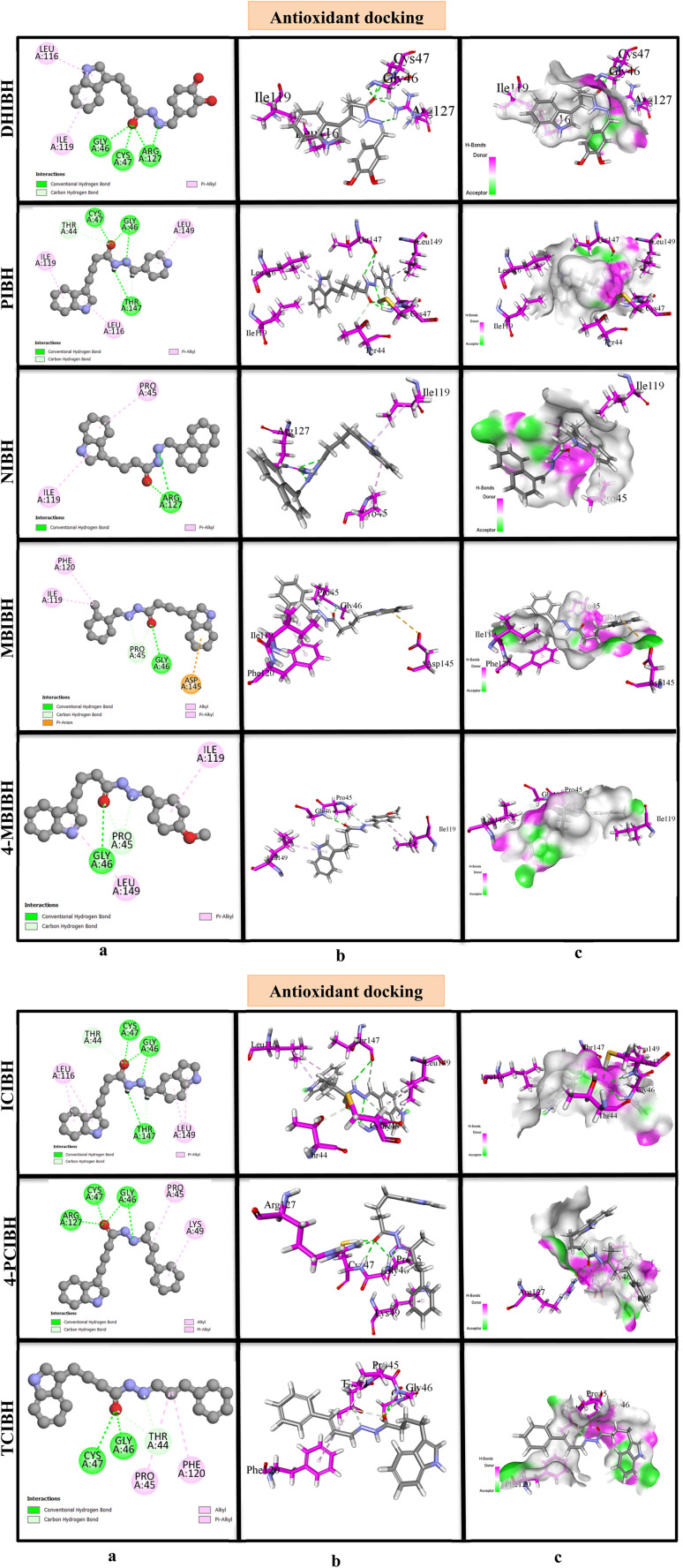
Molecular docking analysis of antioxidant compounds with DHIBH, PIBH, NIBH, MBIBH, 4-MBIBH, ICIBH, 4-PCIBH and TCIBH. (a) 2D interaction diagram showing the hydrogen bonding and hydrophobic interactions between the ligands and active site residues. (b) 3D binding interactions illustrating the ligand orientation within the binding pocket and binding interactions with surrounding residues. (c) Surface representation of the protein–ligand complexes highlighting the hydrogen bond donor and acceptor regions and hydrophobic contact areas.

The pattern of analyzing the 2D interaction diagrams reveals that the conventional hydrogen bonding, π-alkyl, and hydrophobic interaction with the residue GLY46, CYS47, ARG127, THR147, PRO45, ILE119, PHE120 and LYS49 are predominant in antioxidant binding. The hydrazone moiety, amide carbonyl oxygen and indolic nitrogen are always important anchoring sites. Short and directional hydrogen bonds are observed in the hydrogen-bonding diagrams and are usually between 2.09 and 2.53 Å, which are strong polar interactions that hold the ligand–protein complexes stable (Fig. 7).

NIBH with the highest favorable antioxidant docking score (−6.0 kcal mol^−1^, RMSD 1.912 Å) has several hydrogen bonds with ARG127, and π-alkyl interactions with ILE119 and PRO45. The long naphthyl aromatic system promotes the 3D delocalization of electrons and hydrophobic surface area through which it is able to penetrate farther into the antioxidant pocket, which is easily observed in the 3D docked pose with the aromatic moiety in a hydrophobic groove and the hydrazone linkage in an optimal orientation to polar residues.

The 4-PCIBH has a very interaction-rich antioxidant profile with a slightly reduced docking score. Multiple hydrogen bonds are indicated as strong with GLY46 (2.53 Å), CYS47 (2.09 Å) and ARG127 (2.23 Å), and this is backed by hydrophobic contacts with PRO45 and LYS49. The chloro substituent enhances lipophilicity and pocket complementarity, while the hydrazone-amide structure guarantees high levels of electrostatic anchoring. This dual polar-hydrophobic binding mode was observed in the 2D interaction map and in the 3D complex orientation.

The moderate antioxidant binding observed between ICIBH and DHIBH is due to an equal balance of hydrogen bonding and alkyl interactions; TCIBH, which is the weakest in antioxidant docking (score −5.2 kcal mol^−1^), has fewer hydrogen bonds and depends more on hydrophobic contacts (resulting in reduced binding within the binding site).

In general, antioxidant docking behavior significantly correlates with the length of π-conjugation, as shown in Table S1 (SI), the presence of heteroatoms, and electronic delocalization. Docking indicates that NIBH is the best binder for 1HD2.

#### 3.4.2 Antibacterial activity (DNA gyrase B, PDB ID 6F86)

The docking studies of *Escherichia coli* DNA gyrase B (PDB ID: 6F86) have strong molecular evidence in the presence of the antibacterial activity of the synthesized compounds. DHIBH (−6.8 kcal mol^−1^), MBIBH (−6.4 kcal mol^−1^), 4-PCIBH (−6.4 kcal mol^−1^), 4-MBIBH (−6.3 kcal mol^−1^), NIBH (−6.1 kcal mol^−1^), TCIBH (−6.1 kcal mol^−1^), and ICIBH (−6.0 kcal mol^−1^) are in order of antibacterial docking scores with stable binding conformations.

**Fig. 8:**
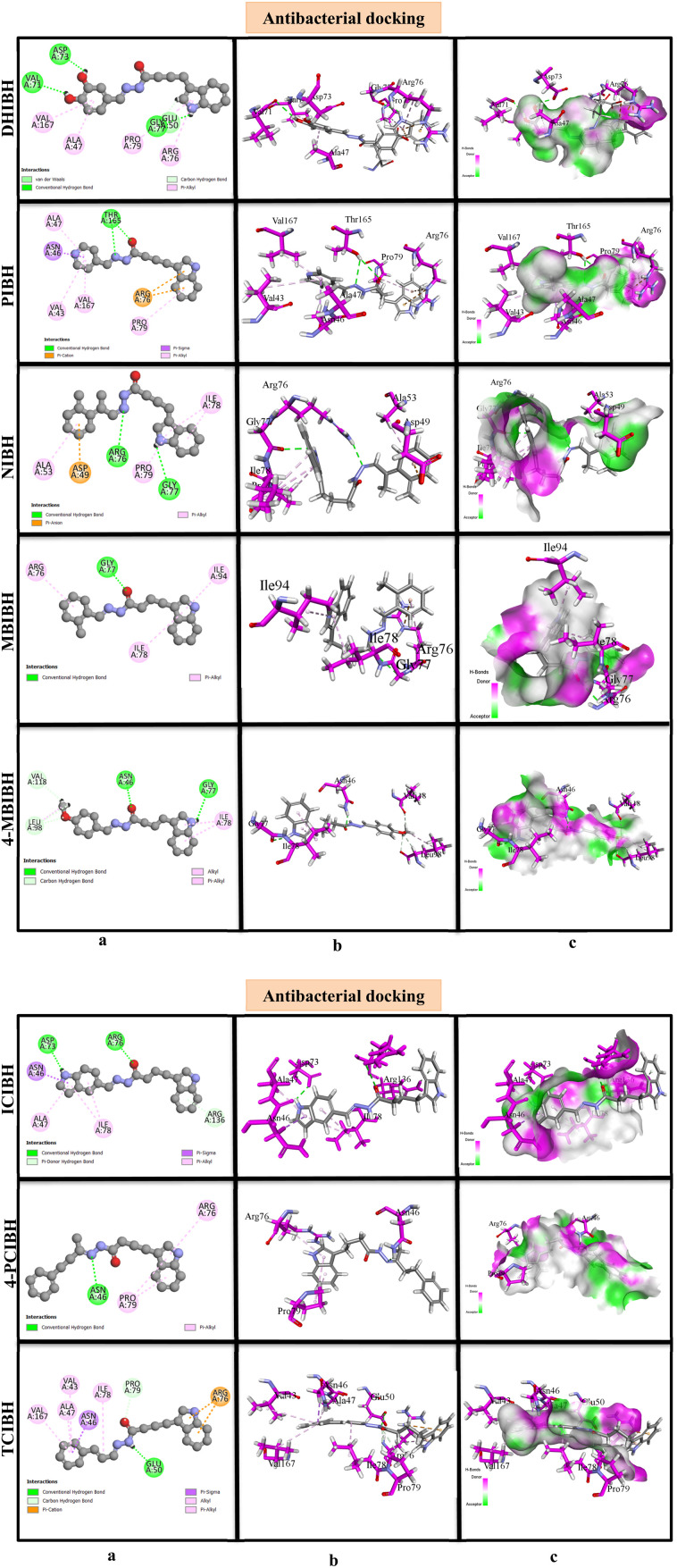
Molecular docking analysis of antibacterial compounds with DHIBH, PIBH, NIBH, MBIBH, 4-MBIBH, ICIBH, 4-PCIBH and TCIBH. (a) 2D interaction diagram showing the hydrogen bonding and hydrophobic interactions between the ligands and active site residues. (b) 3D binding interactions illustrating the ligand orientation within the binding pocket and the binding interactions with surrounding residues. (c) Surface representation of the protein–ligand complexes highlighting the hydrogen bond donor and acceptor regions and hydrophobic contact areas.

GLY77, ASP73, ARG76, PRO79, ALA47, ILE78, VAL167, and ASN46 are always determined as important active-site residues in 2D interaction diagrams. DHIBH with the highest binding affinity (−6.8 kcal mol^−1^) has a dense interaction network consisting of three strong conventional hydrogen bonds with ASP73 (1.9 Å), GLY77 (2.3 Å), and VAL71 (2.5 Å). Additional stabilization is caused by π-cation and π-anion interactions with ARG76 and many π-alkyl interactions between ARG76, PRO79, ALA47, and VAL167. Interactions are well demonstrated in the hydrogen-bonding and 3D docked models, in which DHIBH is in great detail embedded into the gyrase-B catalytic pocket (Fig. 8).

MBIBH and 4-MBIBH indicate that the effect of methoxy substitution on antibacterial binding is that 4-PCIBH has more hydrogen bonding with ASN46 and GLY77 and more carbon–hydrogen bonding interactions with LEU98 and VAL118, which leads to a high docking score and low RMSD. Conversely, the least potent antibacterial binder, PIBH, has fewer hydrogen bonds (especially with THR165); instead, it depends predominantly on less strong hydrophobic interactions, which are indicated in its spacer 2D interaction map and worse 3D orientation. Taken together, the data of the antibacterial docking reveal that compounds with multiple hydrogen-bond donors and acceptors (phenolic OH, hydrazone NH, and carbonyl O) and aromatic systems with the ability of 2-dimensional interaction (2 stacks) and intermolecular electrostatic interactions exhibit better interactions with DNA gyrase B. The high concordance between the docking activity of DHIBH and the maximum inhibitory zone of activity of the enzyme, which is experimentally determined, is strong evidence that DNA gyrase B inhibition is a potential antibacterial process. This suggests that the compound may inhibit bacterial growth by interfering with DNA replication.

The selection of target proteins was based on their well-established biological relevance. DNA gyrase B is a validated antibacterial target responsible for ATP-dependent DNA supercoiling, and its inhibition is a proven strategy in antimicrobial drug development. For antioxidant studies, human peroxiredoxin 5 was selected due to its role in cellular redox homeostasis and peroxide detoxification. Compounds capable of interacting with their proteins may influence oxidative stress pathways. Thus, both targets were chosen to provide a mechanistic basis for the experimentally observed antibacterial and antioxidant activities. Overall, molecular docking results provide mechanistic support for enzyme inhibition by demonstrating that synthesized compounds bind preferentially within the active sites of antioxidant and antibacterial proteins.

The docking scores and biological assay results reported in this study provide insight into the structure–activity relationships of synthesized hydrazone compounds; their interpretation must be approached with careful consideration of both their relevance and inherent limitations. Docking scores serve as a relative indicator of ligand–protein binding affinity, where more negative values generally reflect stronger predicted interactions and enhanced stability of the ligand–enzyme complex. In the present study, DHIBH shows comparatively favorable docking values toward DNA gyrase B, which is observed to some extent with its antibacterial activity. This suggests that the effective occupation of the ATP-binding pocket may contribute to its biological performance or disrupt the proper orientation of catalytic residues, thereby potentially inhibiting enzyme function. In addition, detailed interaction profiles, particularly hydrogen bonding and hydrophobic contacts within the binding cavity, may contribute to the stabilization of the ligand–protein complex and support the inhibitory mechanism.

The rank of docking scores across all eight compounds is in strong agreement with the experimental inhibition zones, with molecules displaying more hydrogen bond donors and aromatic π-systems achieving both higher binding affinities and greater antibacterial potency. Compounds with less productive contact showed correspondingly weaker antibacterial activity. Despite these observations, the predictive capability of docking remains limited by simplification within scoring functions, which do not adequately capture protein flexibility, solvent effect, entropic contribution and the dynamic nature of biological systems. Consequently, small differences in binding energies (<1–2 kcal mol^−1^) should not be interpreted as definitive evidence of superior activity.

Notably, several inconsistencies between the computational predictions and the experimental findings were identified. NIBHDH displayed one of the most favorable docking scores in antioxidant-related docking yet exhibited relatively weak radical scavenging activity, while 4-PCIBH exhibited the highest antioxidant activity but only moderate docking affinity. ICIBH showed strong antioxidant potential but lacked antibacterial activity even though it formed reasonable interactions with the bacterial target. These discrepancies indicate that biological activity is influenced by multiple factors beyond binding affinity, including solubility, membrane permeability, molecular stability and alternate mechanisms, such as direct free radical scavenging.

The integration of docking and experimental results offers complementary perspectives on the potential bioactivity of these molecules; the findings should be interpreted with appropriate caution. Molecular docking should be regarded as a supportive tool for elucidating possible molecular interactions and guiding molecular design, rather than as definitive proof of biological activity. Establishing a stronger correlation between computational predictions and experimental outcomes requires further validation through quantitative biochemical assays and kinetic studies to better capture the dynamic behavior of ligand–protein interaction.

Collectively, these results suggest that the inhibition of DNA gyrase B is likely the primary mechanism underlying antibacterial activity, while the modulation of peroxiredoxin 5 may contribute to a secondary enzyme-mediated pathway in antioxidant activity, complementing the direct radical scavenging activity observed in the DPPH assay.

#### 3.4.3 Docking validation

**Fig. 9:**
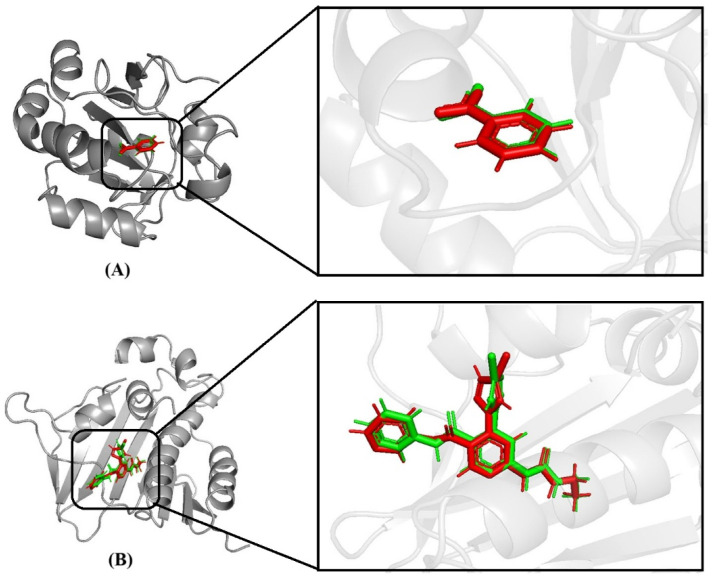
Docking validation results showing the redocking of the native co-crystallized ligand into the active site target protein. Superimposition of the experimentally observed and re-docked ligand conformations within the binding pockets of (A) PDB ID: 1HD2 and (B) PDB ID: 6F86.

The docking protocol was validated by extracting and re-docking the native co-crystallized ligands into the active site of 1HD2 and 6F86 protein using AutoDock Tool 1.5.7. The computationally reproduced binding conformations exhibited excellent spatial agreement with the experimentally resolved crystallographic orientation, thereby confirming the precision and validity of the docking approach. The successful superimposition of re-docked and reference poses demonstrates that the grid parameters, score functions and search algorithms were appropriately calibrated for reliable ligand binding predictions. Validation is crucial for ensuring the robustness and dependability of the docking protocol used for subsequent binding affinity and interaction analyses (Fig. 9).

### 3.5 ADMET analysis

**Fig. 10:**
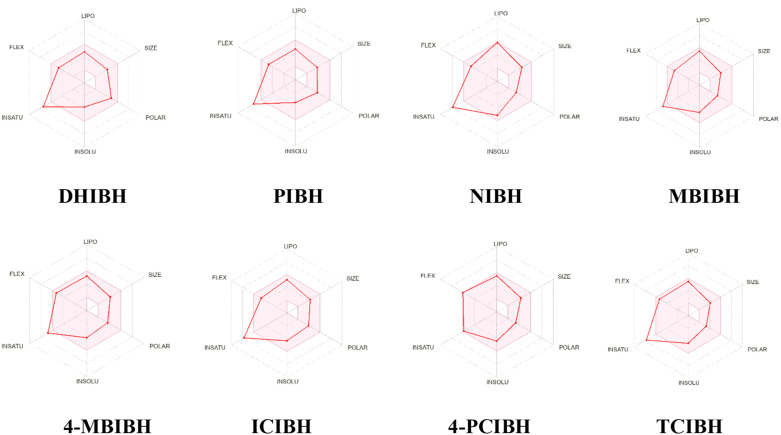
SwissADME bioavailability radar of the DHIBH, PIBH, NIBH, MBIBH, 4-MBIBH, ICIBH, 4-PCIBH and TCIBH compounds.

The pharmacokinetic and drug-likeness profiles of all synthesized derivatives were computationally predicted using the SwissADME web server. Key parameters evaluated included compliance with Lipinski's rule violations, BBB penetration, bioavailability score, GI absorption, PAINS alerts, lead likeness violations and log *K*_p_ (cm s^−1^) (Table 2 and Fig. 10). The bioavailability radar plots offer a consolidated visual summary of six critical physicochemical properties (lipophilicity, molecular size, polarity, aqueous solubility, saturation, and flexibility) that collectively define the drug-like chemical space. In these plots, the red contour traces the compound's physicochemical space, while the shaded pink zone delineates the optimal range for orally bioavailable drugs. Any excursion of the red line beyond the pink boundary indicates a potential limitation in drug-like behavior. Notably, all compounds displayed molecular weights within acceptable limits and exhibited full compliance with Lipinski's criteria without any violations, indicating promising oral drug-like characteristics.

**Table 2:** ADMET profiling results of DHIBH, PIBH, NIBH, MBIBH, 4-MBIBH, ICIBH, 4-PCIBH and TCIBH determined by SwissADME

Compound	MW (g mol^−1^)	Rotatable bonds	H-bond acceptors	H-bond donors	GI	BBB	log *K*_p_ (cm s^−1^)	Lipinski violations	BS	PAINS alert	Lead-likeness violations
DHIBH	337.37	7	4	4	High	No	−6.25	0	0.55	2	0
PIBH	306.36	7	3	2	High	Yes	−6.32	0	0.55	0	0
NIBH	355.43	7	2	2	High	Yes	−4.97	0	0.55	0	2
MBIBH	319.4	7	2	2	High	Yes	−5.37	0	0.55	0	1
4-MBIBH	335.4	8	3	2	High	Yes	−5.75	0	0.55	0	2
ICIBH	344.41	7	2	3	High	Yes	−5.8	0	0.55	0	1
4-PCIBH	347.45	9	2	2	High	Yes	−5.48	0	0.55	0	2
TCIBH	331.41	8	2	2	High	Yes	−5.4	0	0.55	0	2

### 3.6 DFT analysis

#### 3.6.1 Optimized geometries

**Fig. 11:**
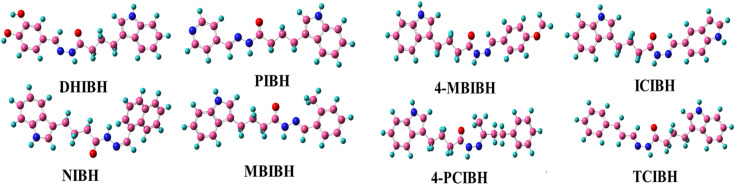
Optimized geometries of all the designed compounds.

All the synthesized hydrazone derivatives are based on the same type of hydrazone structure (indole-3-butyric acid), with three major structural units: (i) indole nucleus, (ii) hydrazone linkage (–CONH–NH–C=N–), and (iii) substituted aromatic or heteroaromatic terminals. DFT at the B3LYP/6-311+G(d,p) level was used to obtain optimized molecular geometries (Fig. 11) that provide information on electronic delocalization and structural stability.

The indole ring preserved in all derivatives serves as a rigid π-conjugated system and provides hydrogen-bonding properties by the indolic NH group, which promotes intermolecular engagements. The main pharmacophoric group is the hydrazone moiety, in which the conjugation between the carbonyl group and the azomethine (C=N) bond facilitates electron delocalization, as confirmed by the optimized structures. Several hydrogen-bond acceptor and donor sites are also present here, which increases chemical reactivity and biological relevance. The different derivatives vary in their terminal substituents, which creates structural differences between the derivatives. DHIBH has a dihydroxyphenyl group added, which adds powerful donating hydroxyl functionalities and increases polarity and hydrogen-bonding capacity. PIBH and NIBH have heteroaromatic pyridyl and extended naphthyl systems, respectively, which enhance π-surface area and conjugation length. MBIBH and 4-MBIBH are differentiated by the position of the methoxy group, which affects the planarity of molecules and their electronic distribution. ICIBH also has an extra indole moiety, increasing the level of heteroatoms and aromatic conjugation with the electron-withdrawing effects observed in 4-PCIBH, controlling charge-distribution and the increase in π-conjugation in TCIBH as a result of its styryl extension.

#### 3.6.2 Frontier molecular orbitals (FMOs)

FMOs are the highest occupied molecular orbital (HOMO)^52^ and the lowest unoccupied molecular orbital (LUMO), which are crucial determinants of the electronic, optoelectronic, and charge-transfer characteristics of organic molecules.^53^ The HOMO–LUMO energy gap (Δ*E*) is commonly employed as a measure of chemical reactivity, kinetic stability and intramolecular charge-transfer efficiency.

Table 3 shows that the calculated energies of HOMO and LUMO at the B3LYP/6-311+G(d,p) level (see Table 3) are significantly different among the derivatives of hydrazone, which is caused by the electronic effect of substituents. The HOMO energies show limited variation (−5.27 to −5.36 eV), which suggests a similar capability to donate electrons in the indole-hydrazone backbone. Conversely, there is increased dispersion in the LUMO energies representing the impact of terminal substituents on the electron-accepting nature. Consequently, the HOMO–LUMO gaps (Δ*E*) range from 3.570 to 5.109 eV, indicating that there are important structural property links.

**Table 3:** Calculated values of HOMO and LUMO in eV of all the synthesized compounds

Molecule	HOMO (eV)	LUMO (eV)	Δ*E* = *E*_LUMO_ − *E*_HOMO_ (eV)
DHIBH	−5.304	−1.057	4.247
PIBH	−5.363	−1.792	3.570
NIBH	−5.270	−1.684	3.586
MBIBH	−5.299	−1.291	4.009
4-MBIBH	−5.310	−1.030	4.280
ICIBH	−5.278	−0.848	4.430
4-PCIBH	−5.304	−0.195	5.109
TCIBH	−5.331	−1.605	3.726

The lowest energy gaps are observed in compounds PIBH (3.570 eV) and NIBH (3.586 eV), and this fact can be explained by the increased π-conjugation and the increased aromaticity of the model compounds (pyridyl and naphthyl units) that stabilize the LUMO and allow intramolecular charge transfer. The gap is also lower in TCIBH (3.726 eV) because it has a longer styryl conjugation. Conversely, the broadest gap is at 4-PCIBH (5.109 eV), which is in line with the great electron-withdrawing influence of the *para*-chloro substituent that destabilizes charge delocalization and enhances molecular hardness. Intermediate gaps occur in ICIBH (4.430 eV) and 4-MBIBH (4.280 eV), indicating a compromise between heteroaromatic conjugation and steric/electronic modulation by substituents.

The general pattern of the HOMO–LUMO gap is as follows: PIBH < NIBH < TCIBH < MBIBH < DHIBH < 4-MBIBH < ICIBH < 4-PCIBH.

Using the orbital contour plots, it is possible to determine that red and blue isosurfaces indicate opposite phases of the molecular wavefunction. The orbital contour plots give more insight into these trends. The red and blue lobes shown in all derivatives are opposite phases of the molecular wavefunction, which depict the geometrical distribution and phase symmetry of the FMOs. The HOMO density is mainly concentrated in the indole nucleus and hydrazone linker areas, indicating that these regions have the highest number of electrons as donors. The presence of heteroatoms (N and O) and the conjugation of the C=N bond contribute to this localization and increase the density of electrons and polarizability. Conversely, the LUMO is predominantly concentrated over the terminal aromatic or heteroaromatic substituents, especially in compounds with extended conjugation (PIBH, NIBH, and TCIBH). This change in electron density on the cores of the indole-hydrazone to the substituent on the excitation of the core is a clear indication of an intramolecular charge-transfer (ICT) process. In the case of 4-PCIBH, the chlorine atom is an electron-withdrawing atom that causes the localization of the LUMO region, which leads to diminished charge mobility and an increased gap between the energy levels.

**Fig. 12:**
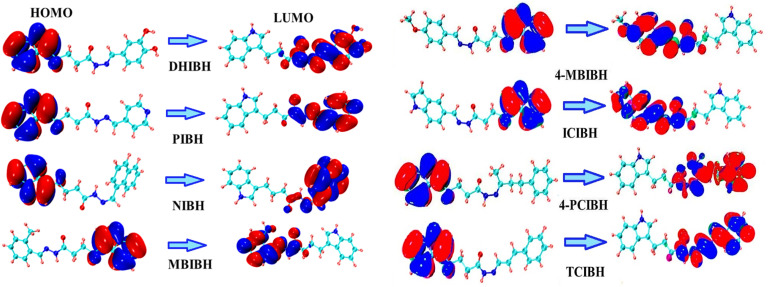
Highest and lowest molecular orbitals of all the synthesized compounds.

In general, the orbital contour analysis allows for the conclusion that the charge-transfer efficiency is dictated by the substituent-controlled modulation of orbital localization and conjugation length, which dictates the electronic behavior of such indole-based hydrazone derivatives, as shown in Fig. 12.

Biologically, the experimentally recorded antioxidant and antibacterial activities can be theoretically explained by the observed FMO qualities. The lower the HOMO-energy gap of the compounds, such as PIBH, NIBH and TCIBH, the greater the chemical reactivity of the compound and the stronger the ability to transfer electrons, which is desirable in radical scavenging and redox-mediated biological processes. The strong HOMO localization on the indole-hydrazone core facilitates electronic donation to support antioxidant functionality, and the well-circumvented LUMO localization on the terminal aromatic moieties supports interactions with biological targets. In contrast, these molecules with higher energy differences, such as 4-PCIBH, exhibit kinetic stability, which can serve as a selective antioxidant activity with regulated electron acceptance. In general, the parameters of FMO calculated with DFT are consistent with the biological screening data, which proves that the modification of electronic structure with the help of substituents is a key factor in determining the antibacterial and antioxidant properties of these indole-based hydrazone derivatives.

#### 3.6.3 Molecular electrostatic potential analysis (MEP)

A strong visual charge distribution, polarity, and reactive site visualization description of a molecule, MEP, is complementary to frontier molecular orbital (FMO) analysis.^54^ Overall, MEP and FMO studies explain the control of intramolecular charge transfer, intermolecular interactions and structure-specific biological activity by electronic structure.

**Fig. 13:**
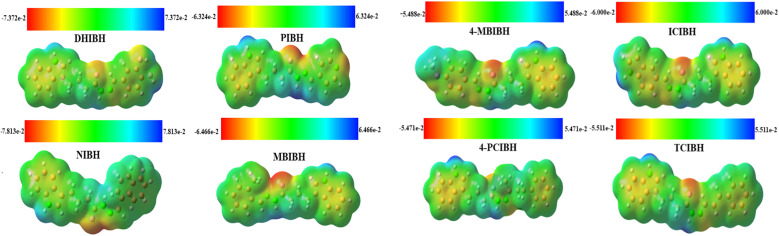
Molecular electrostatic potential surfaces of all the investigated compounds.

The MEP maps of the calculated B3LYP/6-311+G(d,p)-optimized geometries of the indole and hydrazone show the charge distribution within the indole and hydrazone structures, as displayed in Fig. 13. The regions in such color-coded surfaces are red/orange (negative electrostatic potential, *i.e.* electron-rich and nucleophilic), blue (positive electrostatic potential, *i.e.* electron-deficient and electrophilic) and green (near-neutral) in potential. In all derivatives, the most negative potential is always concentrated near the carbonyl oxygen atom and azomethine/hydrazide nitrogen atom, and the hydrazone moiety is the major site of interaction. However, positive potential is primarily observed around the indolic and hydrazide NH protons, indicating that they are hydrogen-bond donors.

The variation in the substituent greatly contributes to the variation in the extent and distribution of the electrostatic potential. DHIBH carrying dihydroxy groups also has stronger negative potentials around the phenolic oxygens, enhancing the local electron density and polarization. PIBH demonstrates a deep dark spot close to the nitrogen of the pyridyl ring, which readily accepts electrons and encourages the charge transfer of the indole-hydrazone core to the heteroaromatic ring on its terminal. NIBH has a more diffuse, dispersed potential across the naphthyl moiety, as expected in the large-scale π-delocalization of the electrons. The positional dynamics of the methoxy group slightly remodel the surface potential in MBIBH and 4-MBIBH, and the *para* isomer exhibits greater delocalization along the conjugated backbone. The addition of the heteroatoms increases polarity in ICIBH and furthers negative regions on the units of indole. The chloro withdrawal group in 4-PCIBH diminishes the amount of electrophilic species on the phenyl ring, directing negative potential on the hydrazone unit. TCIBH has a sustained electrostatic gradient along the extended conjugated system, which is a sign of effective long-range charge communication.

Notably, the trends of the MEP are very supportive in the context of the FMO-based charge-transfer reaction with the localization of HOMO on the core of the indole-hydrazone complex and the extension of LUMO into substituted aromatic units as a means of facilitating intramolecular electron migration. This electronic asymmetry increases the radical scavenging capacity and target-site interactions, which can give a theoretical role to the observed antioxidant activity and antibacterial activity of synthesized hydrazone derivatives.

#### 3.6.4 Global reactivity indices

Conceptual DFT global reactivity descriptors are directly related to biological activity because they measure the electron-donating/accepting potential, charge-transfer propensity, and kinetic stability of a molecule, which are the determinants of redox activity, binding affinity, and intermolecular interactions.^55^ The descriptors most strongly correlated with biological performance include electron affinity (EA), chemical hardness (*η*), softness (*S*), electrophilicity (*ω*) and maximum charge transfer (Δ*N*_max_).

As illustrated in Table 4, the EA order (electron-accepting ability) is PIBH > NIBH > TCIBH > MBIBH > DHIBH > 4-MBIBH > ICIBH > 4-PCIBH.

**Table 4:** Reactivity parameters of all the studied compounds (in eV)^a^

Molecule	IP	EA	*χ*	*Μ*	*η*	*S*	*ω*	Δ*N*_max_
DHIBH	5.304	1.057	3.181	−3.181	2.123	0.235	2.382	1.498
PIBH	5.363	1.792	3.577	−3.577	1.785	0.280	3.585	2.004
NIBH	5.270	1.684	3.477	−3.477	1.793	0.279	3.372	1.939
MBIBH	5.299	1.291	3.295	−3.295	2.004	0.249	2.708	1.644
4-MBIBH	5.310	1.030	3.170	−3.170	2.140	0.234	2.348	1.482
ICIBH	5.278	0.848	3.063	−3.063	2.215	0.226	2.118	1.383
4-PCIBH	5.304	0.195	2.750	−2.750	2.554	0.196	1.480	1.076
TCIBH	5.331	1.605	3.468	−3.468	1.863	0.268	3.229	1.862

a Units are in eV for IP, EA, *χ*, *µ*, *η*, *σ*, and *ω* of the studied compounds. For *σ*, the unit is eV^−1^.

The increased EA in PIBH and NIBH is due to the heteroaromatic substituents (pyridyl and naphthyl), stabilizing the mode of EA LUMO due to extended pi-conjugation, which is more favorable to the uptake of electrons during redox processes. However, 4-PCIBH has the lowest EA because the electron-withdrawing chloro group enhances localization and prevents charge acceptance. Reactivity *versus* stability is expressed through chemical hardness (*η*) and softness (*S*). The *η* order is where 4-PCIBH = 4-MBIBH = DHIBH = MBIBH = TCIBH = NIBH = PIBH, with mildness (*S*), while the opposite is true. The weaker molecules (PIBH, NIBH, and TCIBH) are easier to polarize and respond to chemicals, which is in line with the increased biological interaction. The 4-PCIBH is highly hard, which implies that it is more kinetically stable and less reactive.

An important measure of the reaction with the nucleophilic biological sites is electrophilicity (*ω*): PIBH > NIBH > TCIBH > MBIBH > DHIBH > 4-MBIBH > ICIBH > 4-PCIBH. However, Δ*N*_max_ reflects this sequence, which validates the high charge-accepting capacity of heteroaromatic derivatives. Methoxy replacement controls these parameters subtly; positional (MBIBH*vs.*4-MBIBH) and *para*-substitution (the *para*-substitution slightly decreases electrophilicity because of the stabilization caused by symmetry) effects control delocalization.

These results have a significant relationship with the FMO and MEP analyses. Molecules with larger EA, *ω* and S also have smaller HOMO–LUMO gaps and strong MEP polarization, allowing for efficient intramolecular charge transfer. This *e*-flexibility facilitates radical scavenging (antioxidant activity) and high electrostatic/hydrogen-bonding affinity with bacterial targets and is a consistent theoretical account of the observed trends in biological activity.

#### 3.6.5 UV-visible analysis

UV-visible spectroscopy is a key instrument for investigating electronic transitions and conjugation and hence for determining optoelectronic behavior, including light absorption, charge transfer probability, and excitation energy.^56^ In the case of bioactive π-systems, electron delocalization and redox responsiveness are also manifested in these transitions, which frequently form the basis of antioxidant activity and biomolecular interactions.

**Fig. 14:**
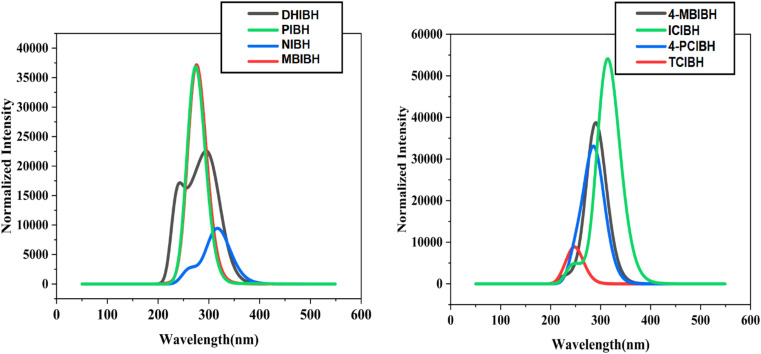
UV-vis spectra of all the designed compounds.

Fig. 14 depicts normalized intensity (*y*-axis) *vs.* wavelength (nm, *x*-axis) spectra with strong absorption bands in the UV region, which are typical of a π → π* transition and n–>π transition in the indole-hydrazone chromophore. Table 5 shows that the *λ*_max_ values lie between 246.4 and 317.46 nm and the excitation energy (*E*_x_) of the system is 3.2771 to 4.7406 eV, with the greatest contribution being HOMO−*n* to LUMO transitions (69–99%).

**Table 5:** Maximum absorption wavelength, excitation energy, oscillation strength, and major electronic contribution of all the studied compounds

Molecule	*λ*_max_ (nm)	*f*_os_	*E*_x_ (eV)	% ECT
DHIBH	302.74	0.4548	3.9254	HOMO−1 → LUMO (92%)
PIBH	275.51	0.8177	3.2771	HOMO−2 → LUMO (97%)
NIBH	317.46	0.2302	3.3127	HOMO−2 → LUMO (95%)
MBIBH	278.30	0.7506	3.7089	HOMO−2 → LUMO (90%)
4-MBIBH	314.4	1.329	3.4394	HOMO−1 → LUMO (99%)
ICIBH	288.0	0.7387	4.1037	HOMO−1 → LUMO (82%)
4-PCIBH	291.1	0.9226	3.9598	HOMO−1 → LUMO (97%)
TCIBH	246.4	0.1293	4.7406	HOMO−2 → LUMO (69%)

There is also an evident structure–property dependence. The red-shifted absorptions in NIBH (317.46 nm) and 4-MBIBH (314.4 nm) correspond to the lowest-energy transitions, which are explained by an increase in π-delocalization: the extended aromatic structure (naphthyl in NIBH) and a good *para*-substitution/conjugation orientation (4-MBIBH) stabilize the excited state and decrease transition energy. In contrast, TCIBH (246.4 nm) shows a strong blue shift and the highest *E*_x_ (4.7406 eV), which agrees with a weaker ICT contribution (69%) and a single excitation led by higher occupied orbitals (HOMO–2LUMO), implying weak donor–acceptor coupling in the most important excitation. Intermediate 6-max values are observed with PIBH (275.51 nm) and MBIBH (278.30 nm), indicating a moderate degree of conjugation and substituent-controlled polarization, while DHIBH (302.74 nm) has the advantage of electron-donating hydroxyl groups, which enhance the delocalization of charge and red-shift absorption compared to less donating substituents. The comparatively good oscillator strengths, especially of 4-MBIBH (*f*_os_ = 1.329) and 4-PCIBH (0.9226), indicate higher transition probabilities, which are linked to better orbital overlaps in the major excitation channels.

Connected to previous discussions, it is associated with the UV-vis trends: compounds with stronger ICT character (high HOMO−*n* to LUMO contribution) and stabilized excited state are associated with more polarized MEP surfaces and effective redistribution of charges. These characteristics can coexist with increased electron-transfer capacity, justifying antioxidant capability, and optimized electronic distribution and transition intensity could also bias target-site interactions, which would be beneficial for antibacterial action.

#### 3.6.6 Density of state (DOS)

DOS analysis gives a quantitative representation of available electronic states around the frontier region by which charge-transport, optical response and excitation pathways in the π-conjugated systems are explained.^57^ DOS characteristics in bioactive molecules also indicate the capacity to donate electrons and accept electrons as well as the accessibility of redox potential that may affect antioxidant and binding interactions. The *x*-axis (energy, eV) of the DOS plots is the orbital energy with respect to the reference level, and the *y*-axis (relative intensity) is the number of states available at this energy. The blue trace represents the total DOS, and the black and red curves are the contributions of the two major fragments (butanehydrazide core and the substituted aldehyde/ketone moiety). The observation of a distinct low-DOS region about the frontier energies agrees with the calculated HOMO–LUMO gaps, and the striking peaks at lower (more negative) energies are attributed to σ/π states further located deeper within the bandgap. In all the derivatives, the profile of DOS confirms that both fragments play a role in the frontier region, yet the quantity of their overlap varies with substitution, indicating varying levels of electronic coupling.

**Fig. 15:**
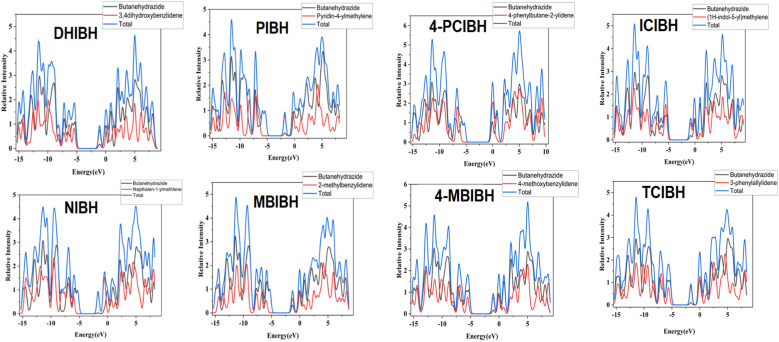
DOS spectra of all the studied compounds.

More highly conjugated and heteroaromatic analogues (*e.g.*PIBH, NIBH, and TCIBH) are more likely to have the mixing of fragment DOS around the frontier energies, and this should happen due to the increased delocalization and intramolecular charge transfer. In comparison, more localized systems (*e.g.*4-PCIBH) have comparatively lower frontier mixing and, therefore, can be more kinetically stable and have a more localized electronic density. Substituent electronics also modulate the positions and strength of its peaks: densities of accessible states on the aromatic segment are enhanced by electron-rich groups (*e.g.*DHIBH with phenolic OH), and states with the potential accessed in electron acceptance or polarization are created by heteroatoms (pyridyl N in PIBH and another indole N in ICIBH). The change in coupling (positional methoxy effects MBIBH*vs.*4-MBIBH) is observed as changes in frontier DOS overlap (Fig. 15).

In sum, the trends of DOS are consistent with the gaps in the FMO and the polarization of the MEPs, which validates the presence of the charge-transfer excitations observed in the UV-vis spectrum. The molecules with higher frontier-state mixing and greater state availability are likely to be redox responsive, giving the theoretical basis for the antioxidant and antibacterial activities.

#### 3.6.7 Infrared spectrum

IR spectroscopy is a key method for investigating the vibrational properties, bonding conditions, and integrity of the functional groups of organic molecules, which offers straightforward information about their electronic structure and molecular stability. In π-conjugated and heteroatom-rich systems, IR vibrational vibrations are directly related to the delocalization of electrons, the capacity to form hydrogen bonds, and the distribution of charge within the intramolecular framework of systems, which dictate optoelectronic behavior. Moreover, the position and strength of characteristic IR bands corresponding to functional groups of molecules, including NH, OH, CO, and CN, are essential in determining biological activity since they are the ones involved in hydrogen bonding and electrostatic interactions with biological targets. Thus, IR studies conducted using both experimental and DFT computations can provide a solid system for correlating the structural stability, electrical characteristics, and biotic activity of the obtained indole-3-butyric acid-based hydrazone analogs.

##### 3.6.7.1 O–H/N–H vibrations (∼3600–3200 cm^−1^)

**Fig. 16:**
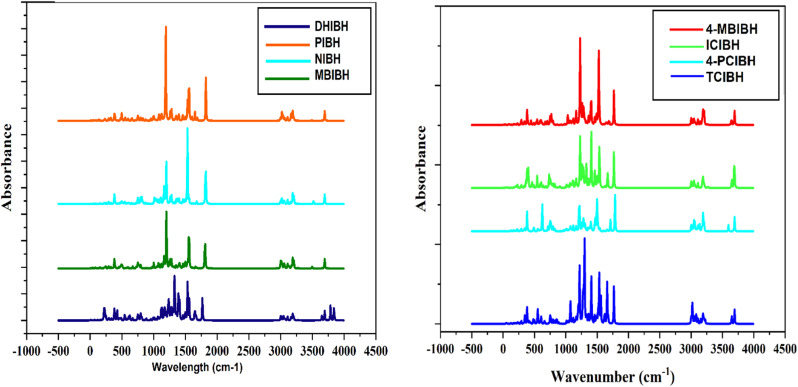
IR spectra of the synthesized compounds.

The DFT-computed IR spectra of DHIBH also have a broad and strong band in the range of 3400–3300 cm^−1^, which is also consistent with the experimental assignment of overlapping OH and NH groups in the range of phenolic hydroxyl groups and hydroxyl hydrazide/indolic NH niches. This expansion is a manifestation of hydrogen-bonding interactions, which are also experimentally observed. The calculated spectra of PIBH, NIBH, MBIBH, 4-MBIBH, ICIBH, 4-PCIBH, and TCIBH also consistently show bands within the same range, which can be attributed to the N3H stretching of the hydrazide and indole units, in good agreement with their corresponding FTIR spectra (about 3400–3300 cm^−1^). Slight deviation in frequencies should be observed as a result of harmonic approximation and gas-phase maximization in DFT (Fig. 16).

##### 3.6.7.2 C–H aromatic and aliphatic stretching vibrations (∼3100–2850 cm^−1^)

Theoretical calculations of all the compounds reproduce the multiple medium intensity bands at around 3050–3000 cm^−1^ based on the aromatic C–H stretching, in agreement with the experimental results for DHIBH, PIBH, NIBH and ICIBH. Further bands in the 2950–2850 cm^−1^ range are attributed to aliphatic C–H (sp^3^) stretching, which is especially intense in MBIBH, 4-MBIBH and TCIBH, and attributed to methoxy (or alkyl/styryl) substituents. The structural assignments are also confirmed by these intensity variations, which are substituent-dependent (Fig. 16).

##### 3.6.7.3 Carbonyl stretching vibrations, *ν*(C=O) (∼1680–1620 cm^−1^)

There is always a strong, sharp DFT-predicted absorption in the 1650–1620 cm^−1^ range of DHIBH, PIBH, NIBH, MBIBH, 4-MBIBH, ICIBH, 4-PCIBH and TCIBH, which is related to amide carbonyl (C=O) stretching vibration. The fact that the experimental FTIR values are close to the values in the close match confirms the presence of the acyl hydrazide functional group in all derivatives. Small red shifts of certain compounds can be ascribed to conjugation with the azomethine group and electronic delocalization of substituents.

##### 3.6.7.4 Azomethine vibrations (*ν*(C=N)) ∼1600–1530 cm^−1^

The calculated IR spectra exhibit clearly defined bands in the range of 1580–1530 cm^−1^ for all derivatives, which are in good agreement with the experimental C=N stretching frequencies. In the case of DHIBH, the calculated band at the 1580–1550 cm^−1^ range coincides with the experimental band, while PIBH, NIBH, MBIBH, 4-MBIBH, ICIBH, 4-PCIBH, and TCIBH present bands at the positions of 1560–1530 cm^−1^. Minor changes between compounds indicate the electronic influences of electron-giving or electron-taking away substituents on the azomethine linkage (Fig. 16).

##### 3.6.7.5 C–N stretching and fingerprint area

The 1350–1200/1150 cm^−1^ DFT predictions are close to the experimental C–N stretching vibrations of all compounds. The high concentration of bands below 1100 cm^−1^ is a result of aromatic ring deformations, in-plane and out-of-plane C–H bending and skeletal vibrations, which clearly demonstrate the presence of substituted aromatic and hetero-aromatic structures.

#### 3.6.8 ELF and LOL analyses

**Fig. 17:**
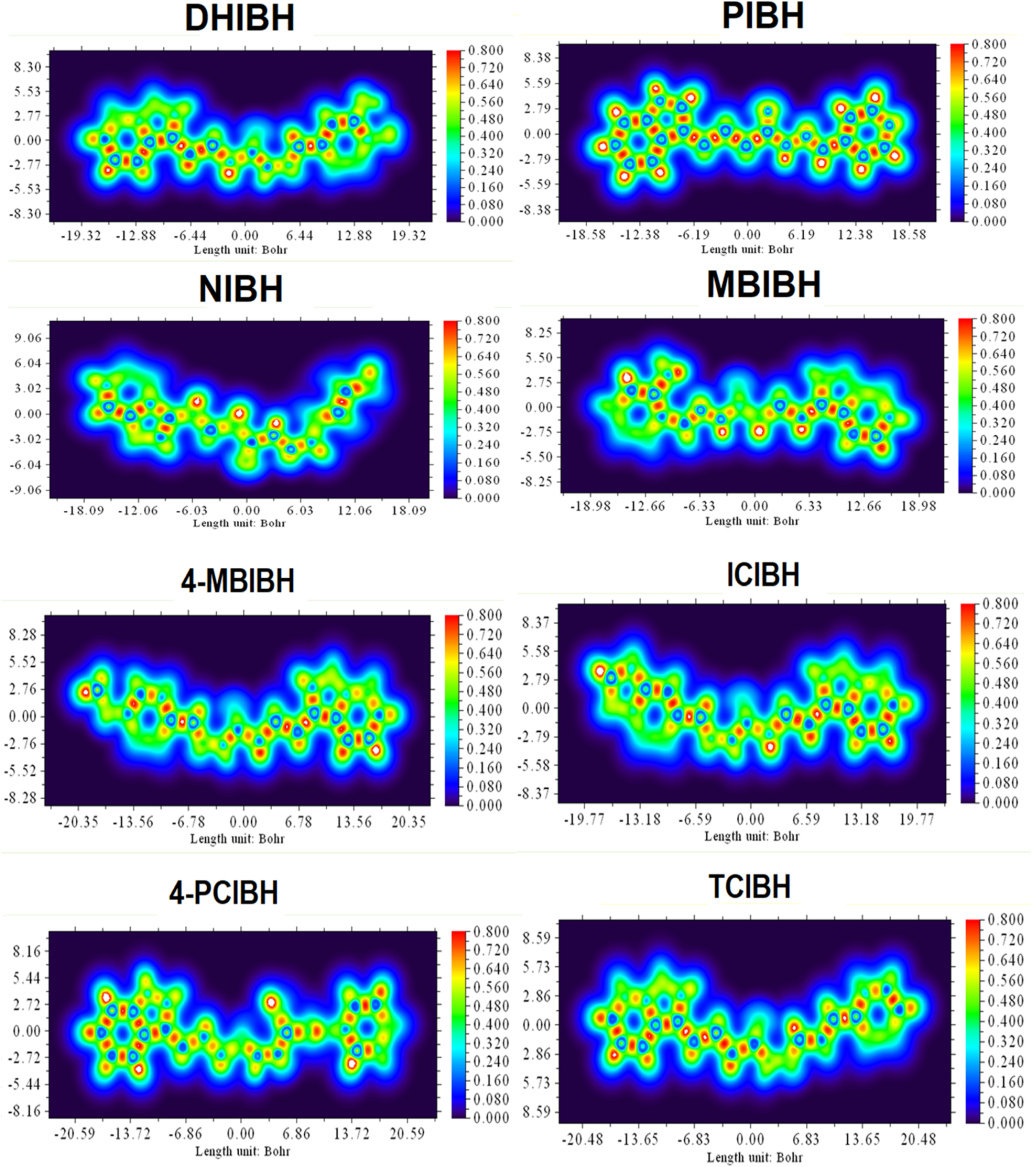
LOL spectra of all the synthesized molecules.

Topologically localized orbital locator (LOL) maps give a visualization of the localization of electrons in real space in the context of the molecular framework. The colors of the given diagrams vary among blue (low localization of electrons), green/yellow (moderate localization of electrons) and red (high localization of electrons). Molecular length in Bohr is used as the *x*-axis, and spatial distribution perpendicular to the molecular backbone is used as the *y*-axis in order to visualize electronic communication along the conjugated system (Fig. 17).

In the case of DHIBH, the LOL map displays a high concentration of red/yellow areas localized around the phenolic oxygen atoms and the hydrazone core, indicating a high level of electron localization favored by hydrogen bonding and lone-pair density. PIBH demonstrates an increase in the localization of the pyridyl nitrogen, which shows the electron-withdrawing heteroaromatic unit and asymmetric charge distribution across the backbone. The longer naphthyl moiety in NIBH has broader green-yellow regions, indicating greater delocalization of π-electrons compared to the fused aromatic system.

The positional effects are evident in the methoxy-substituted MBIBH and 4-MBIBH; *para* substitution (4-MBIBH) provides improved conjugative interaction between rings, resulting in a more continuous localization pattern, while meta substitution provides slightly fragmented localization. The data of the increasing electron localization around the two indole units of ICIBH confirm the presence of heteroatom-assisted charge retention and two-way donor properties. Conversely, 4-PCIBH exhibits smaller red regions around the chloro-substituted ring with less delocalization of the linker, which is expected in the case of an electron-withdrawing effect. TCIBH displays comparatively homogeneous green-yellow outlines in the background, that is, the prolonged and less intense localization is caused by the styryl fragment.

The electron localization function (ELF) is a powerful topological measure of electron pairing, bond character and lone-pair localization, which directly affects charge transport, optical response, and molecular stability. ELF in bioactive scaffolds identifies electron-rich areas that control redox activity and noncovalent interactions (H-bonding and electrostatics) associated with antioxidant and antibacterial activity.

**Fig. 18:**
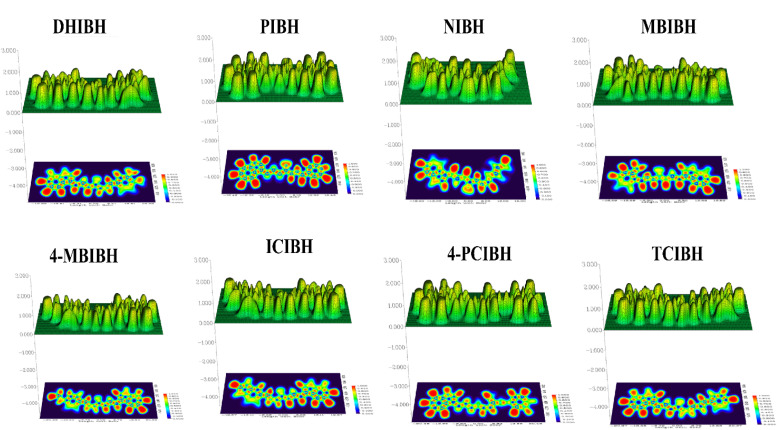
ELF plots of all the designed molecules.

The ELF plots are displayed in the form of 3D surfaces and a color map (2D); the molecular backbone is traced on the *x*-axis (length unit: Bohr), and the spatial distribution of the framework is reflected on the perpendicular axis. In the color scale (intensity of localization), red and yellow indicate the most localized electron density (bonding and lone pairs), while green and blue indicate a more delocalized electron density. High ELF is uniformly observed at heteroatom-rich locations (carbonyl O and hydrazone N atom) and aromatic rings, indicating strong sigma/pi interactions and concentrated lone pairs around the core of the indole-hydrazone moieties (Fig. 18).

A continuity of localization is also modulated along the conjugated path by substituent effects. DHIBH demonstrates increased localization around phenolic O atoms, which agrees with a high lone-pair density and hydrogen-bonding ability. PIBH shows localization around pyridyl N and polarization in the backbone. NIBH is more extensively and continuously localized throughout the extended naphthyl pi-system, which favors delocalization. MBIBH*vs.*4-MBIBH shows that conjugation is positionally controlled by the presence of *para* substitution and allows for easier ELF connectivity compared to the more discontinuous meta analogue. ICIBH displays more localization of two units of indole, but 4-PCIBH displays more localized distributions around the chloro-phenyl end, which is expected of less delocalization. This has TCIBH with long-term though mid-range localization along the styryl fragment.

The trends of LOL and ELFs in general support FMO, MEP, UV-vis and DOS analyses to establish substituent-controlled localization/delocalization and charge-transfer mechanisms. These digital derivatives justify the noted antioxidant redox ability and antibacterial correlations of the hydrazone derivatives.

#### 3.6.9 Natural bond orbital analysis

Natural bond orbital (NBO) is a useful method for measuring intramolecular charge transfer (ICT) due to the interaction between a donor and an acceptor, thus explaining the electronic delocalization applicable to the optoelectronic response and chemical reactivity. These hyperconjugative pathways also explain the redox behavior and the noncovalent binding potential of bioactive hydrazones, which affect antioxidant and antibacterial activity.

Table 6 accentuates the important stabilizing interactions between donor and acceptor orbitals responsible for charge delocalization. Throughout the series, the π → π* interactions are observed within the conjugated systems, *e.g.*, π (C4) → π* (C3) interactions in DHIBH (1.37 kcal mol^−1^), NIBH (1.38 kcal mol^−1^), and TCIBH (1.37 kcal mol^−1^), indicating that the series is uniformly conjugated at π-systems. There is also stronger σ → σ* hyperconjugation with σ (C2–C3) → σ* (C4–C5) in DHIBH (17.56 kcal mol^−1^) and σ (C4–C9) → σ* (C5–C6) in ICIBH (19.96 kcal mol^−1^), indicating that conformationally facilitated charge redistribution happens through the linker.

**Table 6:** Natural bond orbitals of all the designed compounds

Molecule	Donor (i)	Type	Acceptor (*j*)	Type	*E* ^36^ kcal mol^−1^	*E*(*j*) − *E*(*i*) (a.u.)	*F*(*i*; *j*) (a.u.)
DHIBH	π	C4	π*	C3	1.37	11.31	0.111
	σ	C2–C3	σ*	C4–C5	17.56	0.3	0.071
	LP (1)	N16	σ*	C10–N15	10.85	0.83	0.085
	LP (2)	O25	π*	H44	1.7	2.58	0.061
PIBH	π	C6	π*	C5	1.53	10.79	0.115
	σ	C2–C3	σ*	C4–C9	15.44	0.29	0.066
	LP (1)	O14	σ*	C10–C11	2.64	1.05	0.047
	LP (2)	O14	σ*	C10	2.32	2.51	0.071
NIBH	π	C4	π*	C3	1.38	11.31	0.112
	σ	C2–C3	σ*	C4–C9	15.48	0.29	0.066
	LP (1)	N15	σ*	C10–O14	51.21	0.3	0.113
	LP (2)	O14	π*	C10	2.06	2.47	0.066
MBIBH	π	C3	π*	C8	1.8	11.22	0.127
	σ	C3–C8	σ*	C4–C5	19.96	0.28	0.068
	LP (1)	N14	σ*	C15–H36	10.5	0.8	0.083
	LP (2)	O13	π*	C9	2.09	2.47	0.066
4-MBIBH	π	C4–C9	π*	C3	1.37	11.31	0.111
	σ	C4–C9	σ*	C7–C8	18.94	0.27	0.065
	LP (1)	N15	σ*	C10–O14	60.68	0.28	0.119
	LP (2)	O24	π*	C21	2.36	2.27	0.068
ICIBH	π	C5	π*	C6	1.74	10.75	0.122
	σ	C4–C9	σ*	C5–C6	19.96	0.28	0.068
	LP (1)	N15	σ*	C10–O14	61.04	0.28	0.119
	LP (2)	O14	π*	C10	1.93	2.44	0.063
4-PCIBH	π	C3	π*	C4–C5	1.48	10.62	0.113
	σ	C2–C3	σ*	C4–C9	15.42	0.29	0.066
	LP (1)	N1	σ*	C4–C9	35.45	0.3	0.095
	LP (2)	O14	π*	C10	1.79	2.27	0.059
TCIBH	π	C4	π*	C3	1.37	11.32	0.111
	σ	C5–C6	σ*	C7–C8	19.52	0.28	0.067
	LP (1)	N1	σ*	C4–C9	35.45	0.3	0.095
	LP (2)	O14	σ*	C10	1.95	2.45	0.064

Above all, overwhelming stabilization is a result of lone-pair donation on heteroatoms to antibonding σ and π orbitals, which implies that ICT at the hydrazone core is intense. Some examples of representative transitions are LP^36^ N16 → σ* (C10–N15) in DHIBH (10.85 kcal mol^−1^), LP^36^ N15 → σ* (C10–O14) in NIBH (51.21 kcal mol^−1^), LP^36^ N14 → σ* (C15–H36) in MBIBH (10.50 kcal mol^−1^), and LP^36^ N15 → σ* (C10–O14) in 4-MBIBH (60.68 kcal mol^−1^). The greatest *E*^36^ values were found in 4-MBIBH (60.68 kcal mol^−1^) and ICIBH (61.04 kcal mol^−1^) with LP^36^ N15 → σ* (C10–O14), indicating very strong n → σ* interactions with extensive resonance delocalization. Considerable interactions in 4-PCIBH, including LP^36^ N1 → σ* (C4–C9) (35.45 kcal mol^−1^), further support the participation of nitrogen lone pairs in electron delocalization across the hydrazone framework.

Comparing these results, the high HOMO–LUMO energy gap of 4-PCIBH (5.109 eV) indicates greater electronic hardness and kinetic stability, which is consistent with its selective and regulated electron-transfer behaviour. In contrast, compounds with smaller gaps, such as PIBH (3.570 eV) and NIBH (3.586 eV), exhibit greater electronic softness, facilitating electron donation and explaining their relatively stronger DPPH radical scavenging activity. Similarly, the strong NBO interaction of LP(N) → 4-(C=O) in 4-MBIBH (60.68 kcal mol^−1^) indicates the high polarization of the intramolecular charge in the hydrazone structure, which also increases its hydrogen bonding capacity, thus facilitating the increased affinity of the compound with the protein targets and higher docking scores.

The NBO data in general support the FMO/UV-vis charge-transfer transitions and the localization of MEP/ELF at the O/N-rich sites and prove that heteroatom lone pairs contribute to ICT *via* heterodimeric hydrazone linkage. This improved delocalization facilitates electron-transfer mechanisms pertinent to antioxidant activity and increases interaction ability with bacterial targets, which is in line with the recorded biological profiles.

#### 3.6.10 Non-linear optical (NLO) properties

The NLO optical study is crucial in determining how a molecule delocalizes its electrons, efficiency in charge-transfer and response to polarization when it is subjected to an external electric field. These are properties that underlie optoelectronic performance (electro-optic modulation and frequency conversion) and have applications in biological activity, where polarizability and dipole moment affect molecular recognition and redox processes. Donor–pi–acceptor systems are sensitive to substituent-controlled intramolecular charge transfer (ICT).

The dipole moments vary between 1.02 and 3.91 D, showing different degrees of asymmetry of the molecules. The highest *µ* is observed in NIBH (3.91 D) and PIBH (3.57 D), which can be explained by heteroaromatic substituents (naphthyl/pyridyl), increasing charge separation within the indole-hydrazone system. The reduced *µ* values in 4-MBIBH (1.82 D), 4-PCIBH (1.89 D) and TCIBH (1.02 D) indicate reduced donor–acceptor asymmetry owing to substitution effects (Table 7). The dipole moment of all the compounds is larger than that of urea (about 1.37 D), which means that they are more polarized.

**Table 7:** Non-linear optical properties (*µ* = dipole moment (Debye), polarizability (*α*), and hyperpolarizability (*β*)) of all the proposed compounds

Molecule	*µ* (D)	*α* (a.u.)	*β* (a.u.)
DHIBH	3.04	246.86	1408.39
PIBH	3.57	231.59	794.63
NIBH	3.91	272.87	210.67
MBIBH	2.50	250.30	200.51
4-MBIBH	1.82	258.25	1678.20
ICIBH	3.15	269.61	1535.66
4-PCIBH	1.89	255.15	110.15
TCIBH	1.02	281.24	297.09

The values of polarizability (231.59–281.24 a.u.) are on the rise with molecular size and pi-conjugation. TCIBH (281.24 a.u.) and NIBH (272.87 a.u.) exhibit the highest *α* because of the long aromatic system, which makes electron cloud distortion easier. The inductive and resonance effects of methoxy and chloro substitutions (MBIBH/4-MBIBH/4-PCIBH) have a moderate effect on 3. These *α* values are significantly higher than those of urea, which shows greater electronic softness and optical responsiveness.

There is a wide range from 66.15 to 1678.20 a.u. for 4-MBIBH (1678.20 a.u.), ICIBH (1535.66 a.u.) and DHIBH (1408.39 a.u.) due to effective donor–acceptor coupling within the structures. Conversely, the 4-PCIBH (110.15 a.u.) exhibits a suppressed ICT owing to an electron-withdrawing chloro substitution. Significantly, the *β* values, including the largest ones, are multiple times greater than those of urea (*β* = 0.343 a.u.), and this fact highlights the great NLO potential.

In general, the NLO trends are very consistent with the FMO, NBO, MEP, ELF, DOS, and UV-vis analyses, all of which indicate the presence of a substituent-based intramolecular charge transfer along the indole-hydrazone backbone. The compounds with a larger dipole moment and hyperpolarizability have lower HOMO–LUMO gaps, NBO interaction, potent MEP polarization, and higher ELF delocalization, supporting effective electronic communication between segments of donors and acceptors. This shared electronic flexibility is not only the basis of excellent optoelectronic/NLO response but can also be used to explain the observed antioxidant/antibacterial activity, the result of which is dependent on the efficient electron transfer and polarized site of interaction.

### 3.7 Structure–activity relationship analysis

An examination of how different substituents influence biological outcomes reveals distinct electronic and steric factors controlling activity profiles. DHIBH, which carries a 3,4-dihydroxyphenyl group, demonstrated the strongest antibacterial response potency (ZOI: 18.3 mm against *E. coli*; MIC: 2.5 mg mL^−1^) alongside the most effective radical scavenging ability (IC_50_: 44.51 µL mL^−1^). This enhanced performance can be attributed to the presence of two phenolic hydroxyl groups that act as hydrogen-bond donors during interactions with bacterial enzymes and simultaneously facilitate hydrogen atom donation for DPPH radical neutralization.

In contrast, compounds bearing electron-withdrawing groups (4-PCIBH and chlorophenyl ketone) or those devoid of active hydrogen-bond donor functionalities (MBIBH and TCIBH) showed weaker antibacterial activity and were unable to reach 50% DPPH inhibition at the tested concentration range (IC_50_ > 100 µL mL^−1^). PIBH and NIBH occupied intermediate positions, indicating that extended π-conjugation alone increases target binding affinity (supported by docking scores) yet remains inadequate for effective radical scavenging in the absence of active hydrogen-donor groups.

Based on these findings, the SAR ranking for antioxidant activity follows the order catechol-OH (DHIBH) >> heteroaromatic-N (NIBH, PIBH) > methoxy-OMe (4-MBIBH) > halogen/alkyl (4-PCIBH, MBIBH, and TCIBH). The antibacterial activity ordering reflects combined dependencies on hydrogen-bonding ability and membrane permeability: DHIBH > 4-MBIBH > PIBH ≈ TCIBH > 4-PCIBH > NIBH > MBIBH > ICIBH. It is noteworthy that NIBH displays favorable antioxidant docking scores yet weak experimental radical scavenging, emphasizing that binding alone is not a reliable prediction of functional activity, since physicochemical parameters, including aqueous solubility and membrane permeability, also critically determine *in vitro* performance.

## 4 Conclusion

A family of indole-3-butyric acid-based hydrazone compounds (DHIBH, PIBH, NIBH, MBIBH, 4-MBIBH, ICIBH, 4-PCIBH, and TCIBH) was successfully produced, and their structure was confirmed using melting point, FTIR, UV-vis, and NMR spectroscopy. The experimental biological assessment showed that all the compounds have concentration-dependent antioxidant and variable antibacterial properties. DHIBH was found to be the most active antioxidant derivative (84.79% at 100 µL mL^−1^, IC_50_ = 44.51 µL mL^−1^), while DHIBH also demonstrated the largest antibacterial zone of inhibition (18.3 mm against *Escherichia coli*) compared with ciprofloxacin (26.7 mm). However, it should be noted that these values remain considerably lower than those of the reference standards, ascorbic acid (IC_50_: 11.07 µL mL^−1^) and ciprofloxacin (MIC: 0.078 mg mL^−1^ against *E. coli*), indicating that further structural optimization is necessary to achieve clinically relevant potency. The DFT computations were a powerful indication of the experimental results. Frontier molecular orbital analysis showed that HOMO–LUMO energy gaps are between 3.57 and 5.11 eV, which depend on the substituent and are therefore capable of chemical reactivity and charge-transfer. The calculations of UV-vis indicated the predominance of π–π and n–π transitions with bathochromic shifts, showing derivatives with more extended conjugation. ELF, MEP and LOL studies revealed specific sites of electron localization and delocalization, which were associated with hydrogen-bonding and radical stabilization. Global reactivity descriptors also proved the increased electrophilicity and softness of the most bioactive compounds. The NBO analysis revealed good intramolecular interactions between charge transfers, especially between the LP (N/O) and *σ*/pi transitions. Enhanced values of the first hyperpolarizability (8 up to 1678 a.u.) were calculated using nonlinear optical (NLO) measurements and were found to be superior to those of urea, indicating multifunctional optoelectronic potential. The molecular docking experiments with DNA gyrase B (PDB ID: 6F86) and an antioxidant-linked protein (PDB ID: 1HD2) revealed a great correspondence with the experimental data. DHIBH had the best antibacterial binding affinity (−6.8 kcal mol^−1^), and NIBH had good docking scores for antioxidants (up to −6.0 kcal mol^−1^) due to the large number of hydrogen bonds and hydrophobic interactions. The ADMET results further confirmed the favorable drug-like and pharmacokinetic properties. Nevertheless, notable discrepancies between docking predictions and experimental outcomes for creation derivatives (*e.g.*NIBH shows favorable docking but weak radical scavenging) highlight limitations of static computational approaches and the need for dynamic validation. Overall, the integrated experimental and computational findings suggest that substituent-controlled electronic modulation significantly influences the antioxidant and antibacterial behaviors of indole-hydrazone derivatives. Among synthesized compounds, DHIBH emerges as the most promising lead scaffold; however, its moderate activity relative to drugs necessitates further medicinal chemistry optimization, like bioisosteric replacement and formulation strategies, to increase potency and bioavailability prior to any advancement toward therapeutic utility.

## Conflicts of interest

The authors declare that they have no conflicts of interest.

## Supplementary Material

RA-016-D6RA01763K-s001

## Data Availability

All data generated or analyzed during this study are included in this published article and its supplementary information (SI). Further datasets files can be obtained from the corresponding author upon reasonable request. Supplementary information is available. See DOI: https://doi.org/10.1039/d6ra01763k.
