# Supplementary material for: Synthesis and structure–activity relationships of indole-3-butyric acid-based hydrazones: predicting their antibacterial and antioxidant potential through integrated experimental, molecular docking and DFT studies
Source: RSC Adv. 2026 Jun 3;16(33):30408–33. doi: 10.1039/d6ra01763k (PMC13234927; doi:10.1039/d6ra01763k)
Supplement: RA-016-D6RA01763K-s001 [file RA-016-D6RA01763K-s001.pdf]

Supplementary Information (SI) for RSC Advances.
This journal is © The Royal Society of Chemistry 2026

Supporting Information

Synthesis and Structure–Activity Relationship of Indole-3-butyric Acid-Based Hydrazones: Predicting their Antibacterial and Antioxidant potential through Integrated Experimental, Molecular Docking and DFT Studies

Mudasara Azam[1], Talha Mashhood,[1] Shehar Bano[1], Muhammad Ibrahim,*[1] Sehar Nadeem,[2] Humaira Zulfiqar,[3] Nora Hamad Al-Shaalan,[4] Sarah Alharthi,[5,6] Mohammed A. Amin,[7] Muhammad Usman Khan,[2]*

[1]Department of Applied Chemistry, Government College University Faisalabad, Faisalabad-38000, Pakistan

[2]Department of Chemistry, University of Okara, Okara-56300, Pakistan

[3]Department of Chemistry, COMSATS University Islamabad, Abbottabad, Pakistan

[4]Department of Chemistry, College of Science, Princess Nourah bint Abdulrahman University, P.O. Box 84428, Riyadh 11671, Saudi Arabia

[5]Department of Chemistry, College of Science, Taif University, P.O. Box 11099, Taif 21944, Saudi Arabia

[6]Center of Advanced Research in Science and Technology, Taif University, P.O. Box 11099, Taif 21944, Saudi Arabia

[7]Chemistry Department, Faculty of Science, Ain Shams University, Abbassia, Cairo, 11566 Egypt

*** Corresponding authors E-mail addresses:**

- **Dr. Muhammad Ibrahim:**
  Email: ibrahim@gcuf.edu.pk
- **Dr. Muhammad Usman Khan**
  Email: usman.chemistry@gmail.com ; usmankhan@uo.edu.pk

Table S1: The interaction table of all synthesized compound with and 6F86 (Antibacterial receptor) and 1HD2 (Antioxidant receptor)

Complexes	Amino acids	Distance	Category	Type
DHIBH-6F86	A:GLY77:O	2.34469	Hydrogen Bond	Conventional Hydrogen Bond
	A:VAL71:O	2.53023	Hydrogen Bond	Conventional Hydrogen Bond
	A:ASP73:OD2	1.95259	Hydrogen Bond	Conventional Hydrogen Bond
	A:GLY77:O	2.87481	Hydrogen Bond	Carbon Hydrogen Bond
	A:ARG76:NE	3.9774	Electrostatic	Pi-Cation
	A:ARG76:NE	3.9262	Electrostatic	Pi-Cation
	A:GLU50:OE2	4.97133	Electrostatic	Pi-Anion
	A:ARG76	5.07042	Hydrophobic	Pi-Alkyl
	A:PRO79	5.25563	Hydrophobic	Pi-Alkyl
	A:ALA47	4.44692	Hydrophobic	Pi-Alkyl
	A:VAL167	5.27395	Hydrophobic	Pi-Alkyl
PIBH-6F86	A:THR165:HG1	3.06715	Hydrogen Bond	Conventional Hydrogen Bond
	A:THR165:HG1	2.96238	Hydrogen Bond	Conventional Hydrogen Bond
	A:ASN46:HB2	2.67615	Hydrophobic	Pi-Sigma
	A:PRO79	5.46044	Hydrophobic	Pi-Alkyl
	A:VAL43	5.47023	Hydrophobic	Pi-Alkyl
	A:ALA47	4.70991	Hydrophobic	Pi-Alkyl
	A:VAL167	5.03075	Hydrophobic	Pi-Alkyl
NIBH-6F86	A:ARG76:HH21	2.77543	Hydrogen Bond	Conventional Hydrogen Bond
	A:GLY77:O	2.76006	Hydrogen Bond	Conventional Hydrogen Bond
	A:ASP49:OD2	3.96838	Electrostatic	Pi-Anion
	A:ILE78	4.92568	Hydrophobic	Pi-Alkyl
	A:PRO79	5.46236	Hydrophobic	Pi-Alkyl
	A:ILE78	4.80199	Hydrophobic	Pi-Alkyl
	A:ALA53	5.36681	Hydrophobic	Pi-Alkyl
MBIBH-6F86	A:GLY77:H	2.97251	Hydrogen Bond	Conventional Hydrogen Bond
	A:ILE78	5.37883	Hydrophobic	Pi-Alkyl
	A:ILE94	5.19886	Hydrophobic	Pi-Alkyl
	A:ARG76	4.95218	Hydrophobic	Pi-Alkyl
4-MBIBH-6F86	A:ASN46:HD22	2.25173	Hydrogen Bond	Conventional Hydrogen Bond
	A:GLY77:O	2.71613	Hydrogen Bond	Conventional Hydrogen Bond
	A:LEU98:HA	2.60353	Hydrogen Bond	Carbon Hydrogen Bond
	A:VAL118:O	2.53502	Hydrogen Bond	Carbon Hydrogen Bond
	A:LEU98:O	2.87166	Hydrogen Bond	Carbon Hydrogen Bond
	A:LEU98	4.61274	Hydrophobic	Alkyl
	A:ILE78	4.65431	Hydrophobic	Pi-Alkyl
	A:ILE78	4.88952	Hydrophobic	Pi-Alkyl
ICIBH-6F86	A:ARG76:HH22	3.00402	Hydrogen Bond	Conventional Hydrogen Bond

	A:ASP73:OD2	2.1924	Hydrogen Bond	Conventional Hydrogen Bond
	A:ARG136:HH21	2.54934	Hydrogen Bond	Pi-Donor Hydrogen Bond
	A:ASN46:HB1	2.66859	Hydrophobic	Pi-Sigma
	A:ALA47	4.65189	Hydrophobic	Pi-Alkyl
	A:ILE78	5.42774	Hydrophobic	Pi-Alkyl
	A:ILE78	4.85283	Hydrophobic	Pi-Alkyl
	A:ASN46:HD22	2.62209	Hydrogen Bond	Conventional Hydrogen Bond
	A:ARG76	5.23801	Hydrophobic	Pi-Alkyl
	A:PRO79	5.02572	Hydrophobic	Pi-Alkyl
4-PCIBH-6F86	A:PRO79	3.97069	Hydrophobic	Pi-Alkyl
	A:GLU50:OE1	2.66617	Hydrogen Bond	Conventional Hydrogen Bond
	A:PRO79:HD1	2.82692	Hydrogen Bond	Carbon Hydrogen Bond
	A:PRO79:HD2	2.68391	Hydrogen Bond	Carbon Hydrogen Bond
	A:ARG76:NE	4.21603	Electrostatic	Pi-Cation
	A:ARG76:NE	4.76996	Electrostatic	Pi-Cation
	A:ASN46:HB1	2.71158	Hydrophobic	Pi-Sigma
	A:ILE78	4.9492	Hydrophobic	Alkyl
	A:ARG76	4.89672	Hydrophobic	Pi-Alkyl
	A:VAL43	5.49907	Hydrophobic	Pi-Alkyl
	A:ALA47	4.60469	Hydrophobic	Pi-Alkyl
TCIBH-6F86	A:VAL167	5.07801	Hydrophobic	Pi-Alkyl
	A:GLY46	2.35623	Hydrogen Bond	Conventional Hydrogen Bond
	A:CYS47	2.27374	Hydrogen Bond	Conventional Hydrogen Bond
	A:CYS47	2.45563	Hydrogen Bond	Conventional Hydrogen Bond
	A:ARG127	2.01333	Hydrogen Bond	Conventional Hydrogen Bond
	A:ARG127	2.63653	Hydrogen Bond	Conventional Hydrogen Bond
	A:GLY46:HA2	2.63471	Hydrogen Bond	Carbon Hydrogen Bond
	A:LEU116	4.8347	Hydrophobic	Pi-Alkyl
DHIBH-1HD2	A:ILE119	5.06123	Hydrophobic	Pi-Alkyl
	A:GLY46:HN	2.13141	Hydrogen Bond	Conventional Hydrogen Bond
	A:GLY46:HN	3.01875	Hydrogen Bond	Conventional Hydrogen Bond
	A:CYS47:HN	2.49813	Hydrogen Bond	Conventional Hydrogen Bond
	A:THR147:O	2.9471	Hydrogen Bond	Conventional Hydrogen Bond
	A:THR44:HB	2.9152	Hydrogen Bond	Carbon Hydrogen Bond
	A:GLY46:HA1	2.57212	Hydrogen Bond	Carbon Hydrogen Bond
	A:THR147:O	2.92857	Hydrogen Bond	Carbon Hydrogen Bond
	A:LEU116	4.6723	Hydrophobic	Pi-Alkyl
	A:ILE119	4.7523	Hydrophobic	Pi-Alkyl
PIBH-1HD2	A:LEU149	4.52152	Hydrophobic	Pi-Alkyl
	A:ARG127:HH21	2.65122	Hydrogen Bond	Conventional Hydrogen Bond
NIBH-1HD2	A:ARG127:HH22	2.83141	Hydrogen Bond	Conventional Hydrogen Bond

	A:ARG127:HH22	2.80994	Hydrogen Bond	Conventional Hydrogen Bond
	A:ILE119	5.05988	Hydrophobic	Pi-Alkyl
	A:PRO45	5.14154	Hydrophobic	Pi-Alkyl
MBIBH-1HD2	A:GLY46:HN	2.71823	Hydrogen Bond	Conventional Hydrogen Bond
	A:PRO45:HD1	2.47086	Hydrogen Bond	Carbon Hydrogen Bond
	A:PRO45:HD1	2.60447	Hydrogen Bond	Carbon Hydrogen Bond
	A:ASP145:OD2	4.43781	Electrostatic	Pi-Anion
	A:ILE119	4.41116	Hydrophobic	Alkyl
	A:PHE120	4.54954	Hydrophobic	Pi-Alkyl
4-MBIBH-1HD2	A:GLY46:HN	2.42032	Hydrogen Bond	Conventional Hydrogen Bond
	A:PRO45:HD1	2.42906	Hydrogen Bond	Carbon Hydrogen Bond
	A:PRO45:HD1	2.59229	Hydrogen Bond	Carbon Hydrogen Bond
	A:LEU149	2.65315	Hydrogen Bond	Carbon Hydrogen Bond
	A:ILE119	5.17603	Hydrophobic	Pi-Alkyl
ICIBH-1HD2	A:GLY46:HN	2.19577	Hydrogen Bond	Conventional Hydrogen Bond
	A:GLY46:HN	3.03707	Hydrogen Bond	Conventional Hydrogen Bond
	A:CYS47:HN	2.41606	Hydrogen Bond	Conventional Hydrogen Bond
	A:CYS47:HG	2.36919	Hydrogen Bond	Conventional Hydrogen Bond
	A:THR147:O	2.99342	Hydrogen Bond	Conventional Hydrogen Bond
	A:THR44:HB	2.99292	Hydrogen Bond	Carbon Hydrogen Bond
	A:GLY46:HA1	2.57335	Hydrogen Bond	Carbon Hydrogen Bond
	A:THR147:O	3.03629	Hydrogen Bond	Carbon Hydrogen Bond
	A:LEU116	4.86554	Hydrophobic	Pi-Alkyl
	A:LEU116	4.95657	Hydrophobic	Pi-Alkyl
	A:LEU149	5.42159	Hydrophobic	Pi-Alkyl
	A:LEU149	4.48856	Hydrophobic	Pi-Alkyl
4-PCIBH-1HD2	A:GLY46:HN	2.52834	Hydrogen Bond	Conventional Hydrogen Bond
	A:GLY46:HN	2.45894	Hydrogen Bond	Conventional Hydrogen Bond
	A:CYS47:HN	2.27907	Hydrogen Bond	Conventional Hydrogen Bond
	A:CYS47:HG	2.08769	Hydrogen Bond	Conventional Hydrogen Bond
	A:ARG127:HH21	2.22947	Hydrogen Bond	Conventional Hydrogen Bond
	A:GLY46:HA1	2.63699	Hydrogen Bond	Carbon Hydrogen Bond
	A:PRO45	5.21987	Hydrophobic	Alkyl
	A:LYS49	5.34278	Hydrophobic	Pi-Alkyl
TCIBH-1HD2	A:GLY46:HN	2.05777	Hydrogen Bond	Conventional Hydrogen Bond
	A:CYS47:HN	2.56681	Hydrogen Bond	Conventional Hydrogen Bond
	A:THR44:HB	3.04421	Hydrogen Bond	Carbon Hydrogen Bond
	A:THR44:HB	2.62796	Hydrogen Bond	Carbon Hydrogen Bond
	A:PRO45	5.20681	Hydrophobic	Alkyl
	A:PHE120	4.78334	Hydrophobic	Pi-Alkyl

2.2.1 *N'-(3,4-dihydroxybenzylidene)-4-(1H-indol-3-yl)butanehydrazide* (DHIBH)

M.P: 131, Yield: 72%

IR ύ$_{max}$ (*cm^{-1}*): 3373 (N-H, amidic) str, 3045 (C-H, sp^2) str, 2914-2868 (C-H, sp^3) str, 1664 (-C=O, amidic), 1533 (-C=N, iminic), 1372 (C-N).

UV $\boldsymbol{\lambda_{max}}$= 368 nm corresponds to π→π*, n→π* transitions of the conjugated hydrazone (–C=N–NH–) system and indicating extended conjugation in the molecule.

^{1}H-NMR (400 MHz, DMSO) δ 10.96 (s, 1H, N-H^{15}, Amide), 10.74 (s, 1H, N-H^{1}), 9.30 (s, 1H, OH^{25}), 9.24 (s, 1H, OH^{24}), 7.94 (s, 1H, Ar-H^{5}), 7.79 (s, 1H, Ar-H^{26}), 7.52 (dd, *J* = 7.9, 4.7 Hz, 1H, Ar-H^{8}), 7.33 (d, *J* = 8.3 Hz, 1H, Ar-H^{23}), 7.18 – 7.16 (m, 1H, Ar-H^{19}), 7.13 – 7.01 (m, 2H, Ar-H^{2,7}), 6.96 (td, *J* = 7.4, 4.1 Hz, 1H, Ar-H^{6}), 6.75 (d, *J* = 8.9 Hz, 1H, Ar-H^{22}), 2.79 – 2.62 (m, 3H, H$^{13'',12}$), 2.22 (t, *J* = 7.4 Hz, 1H, H$^{13'}$), 1.94 (td, *J* = 7.7, 4.3 Hz, 2H, H^{12}).

^{13}C-NMR (101 MHz, DMSO) δ 174.42, 168.69, 147.92, 146.06, 143.48, 136.78, 126.29, 122.77, 122.66, 121.29, 120.78, 120.12, 118.81, 118.57, 116.07, 115.96, 113.08, 111.79, 40.58, 40.37, 40.16, 39.96, 39.75, 39.54, 39.33, 32.41, 26.37, 24.98.

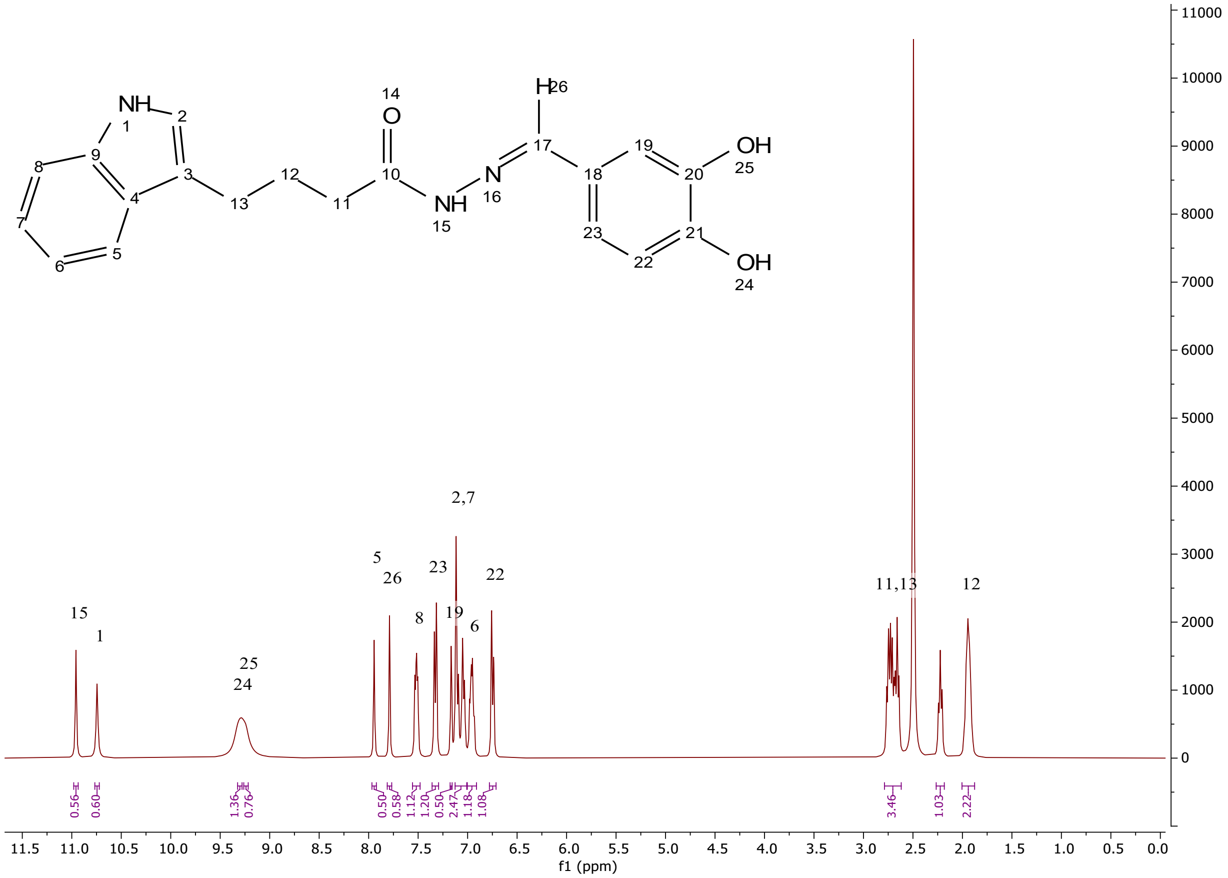

NH
1
2
3
4
5
6
7
8
9
13
12
11
10
O
14
NH
15
N
16
H
26
17
18
19
20
21
22
23
OH
25
OH
24
15
1
25
24
5
26
8
23
2,7
19
6
22
11,13
12
0.56
0.60
1.36
0.76
0.50
0.58
1.12
1.20
0.50
2.47
1.18
1.08
3.46
1.03
2.22
11000
10000
9000
8000
7000
6000
5000
4000
3000
2000
1000
0
11.5
11.0
10.5
10.0
9.5
9.0
8.5
8.0
7.5
7.0
6.5
6.0
5.5
5.0
4.5
4.0
3.5
3.0
2.5
2.0
1.5
1.0
0.5
0.0
f1 (ppm)

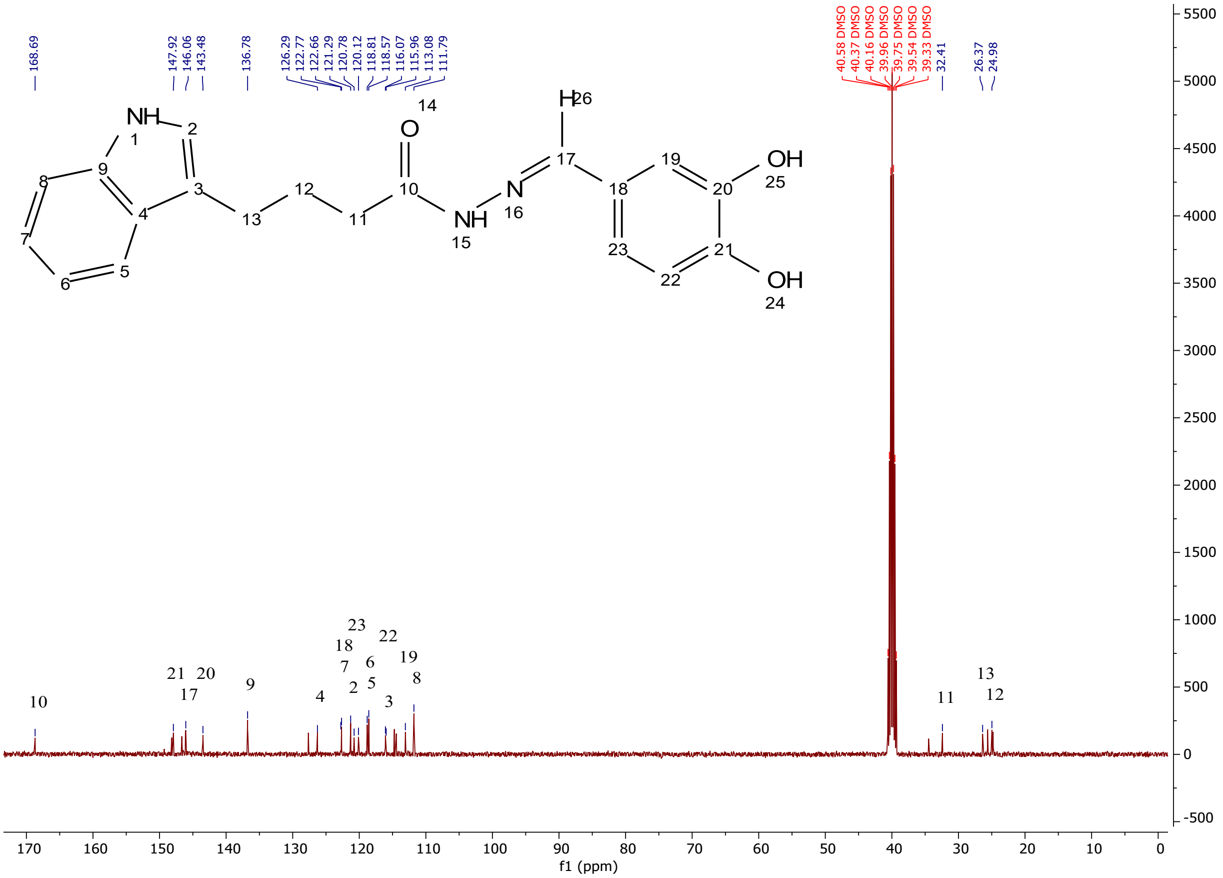

168.69
147.92
146.06
143.48
136.78
126.29
122.77
122.66
121.29
120.78
120.12
118.81
118.57
116.07
115.96
113.08
111.79
40.58 DMSO
40.37 DMSO
40.16 DMSO
39.96 DMSO
39.75 DMSO
39.54 DMSO
39.33 DMSO
32.41
26.37
24.98
NH
OH
OH
O
N
NH
f1 (ppm)

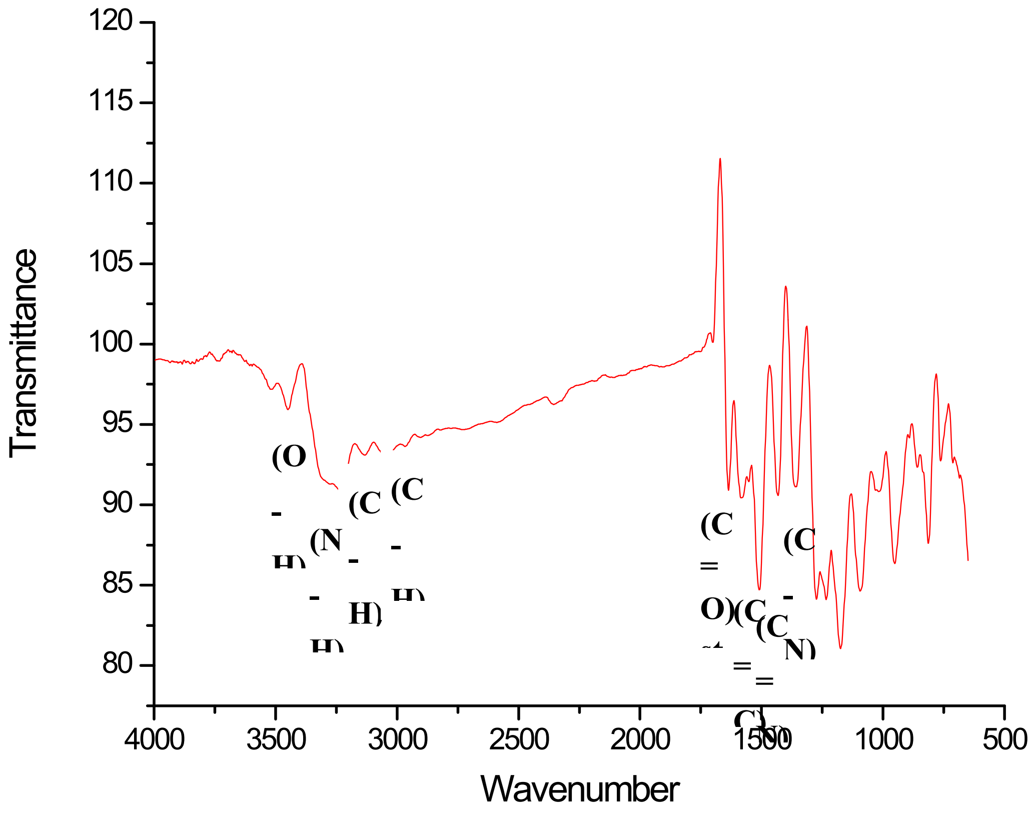
Transmittance
Wavenumber
120
115
110
105
100
95
90
85
80
4000
3500
3000
2500
2000
1500
1000
500
(O
-
H)
(N
-
H)
(C
-
H)
(C
-
H)
(C
=
O)
(C
=
C)
(C
=
N)
(C
-
N)

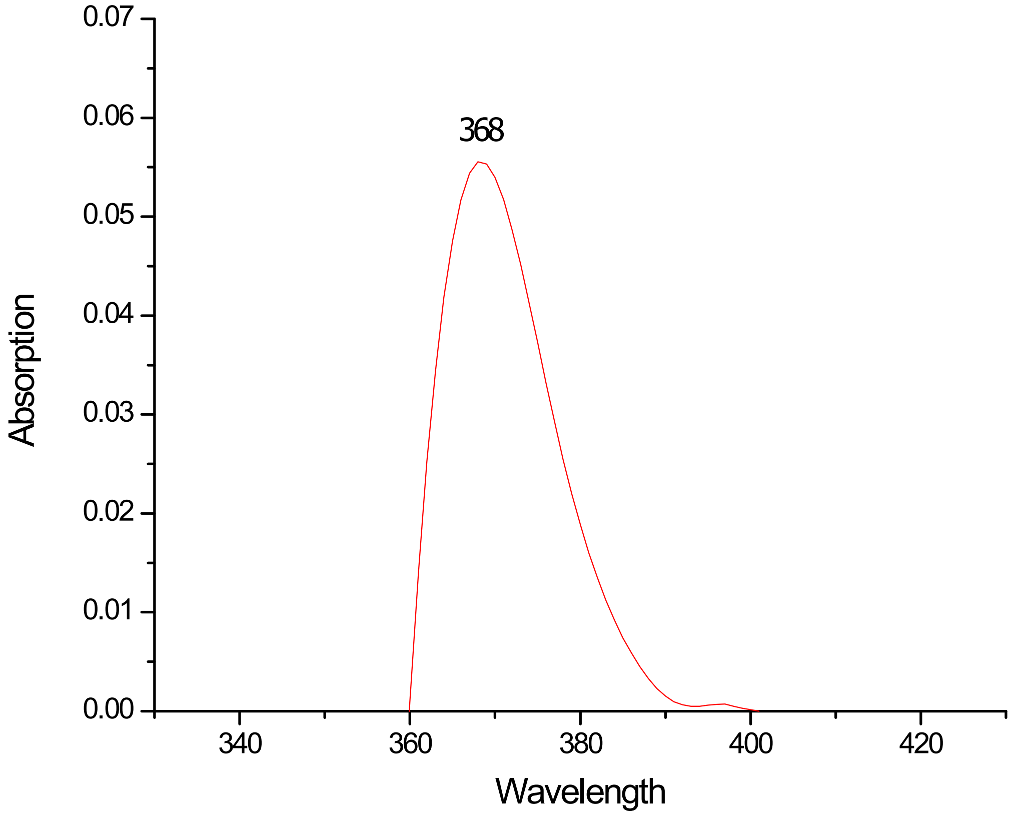
368
Absorption
0.07
0.06
0.05
0.04
0.03
0.02
0.01
0.00
340
360
380
400
420
Wavelength

2.2.2 *(E)-4-(1H-indol-3-yl)-N'-(pyridin-4-ylmethylene)butanehydrazide* (PIBH)

M.P: 129, Yield: 70%

IR $\acute{\upsilon}_{max}$(cm^{-1}): 3343 (N-H, amidic) str, 3055 (C-H, sp^2) str, 2914-2868 (C-H, sp^3) str, 1684 (-C=O, amidic), 1531 (-C=N, iminic), 1361 (C-N).

UV λ_{max}= 335 nm corresponds to π→π*, n→π* transitions of the conjugated hydrazone (–C=N–NH–) system and indicating extended conjugation in the molecule.

^{1}H-NMR (400 MHz, DMSO) δ 11.48 (s, 1H, N-H^1), 10.78 (s, 1H, N-H^{15}, Amide), 8.58 – 8.56 (m, 2H, Ar-$H^{17,21}$), 7.91 (s, 1H, Ar-H^{23}), 7.52 (dd, *J* = 7.9, 3.4 Hz, 1H, Ar-H^8), 7.48 – 7.42 (m, 2H, Ar-$H^{18,20}$), 7.34 (dd, *J* = 8.2, 4.3 Hz, 1H, Ar-H^5), 7.13 (d, *J* = 2.3 Hz, 1H, Ar-H^2), 7.06 (t, *J* = 7.6 Hz, 1H, Ar-H^7), 7.01 – 6.91 (m, 1H, Ar-H^6), 2.81 – 2.66 (m, 3H, $H^{13'',11}$), 2.30 (t, *J* = 7.4 Hz, 1H, $H^{13'}$), 1.96 (dt, *J* = 14.9, 5.5 Hz, 2H, H^{12}).

^{13}C-NMR (101 MHz, DMSO) δ 169.56, 150.65, 150.62, 143.71, 140.25, 136.78, 127.70, 122.84, 121.29, 120.99, 118.79, 118.59, 114.55, 111.81, 40.59, 40.39, 40.18, 39.97, 39.76, 39.55, 39.34, 32.39, 25.84, 24.82.

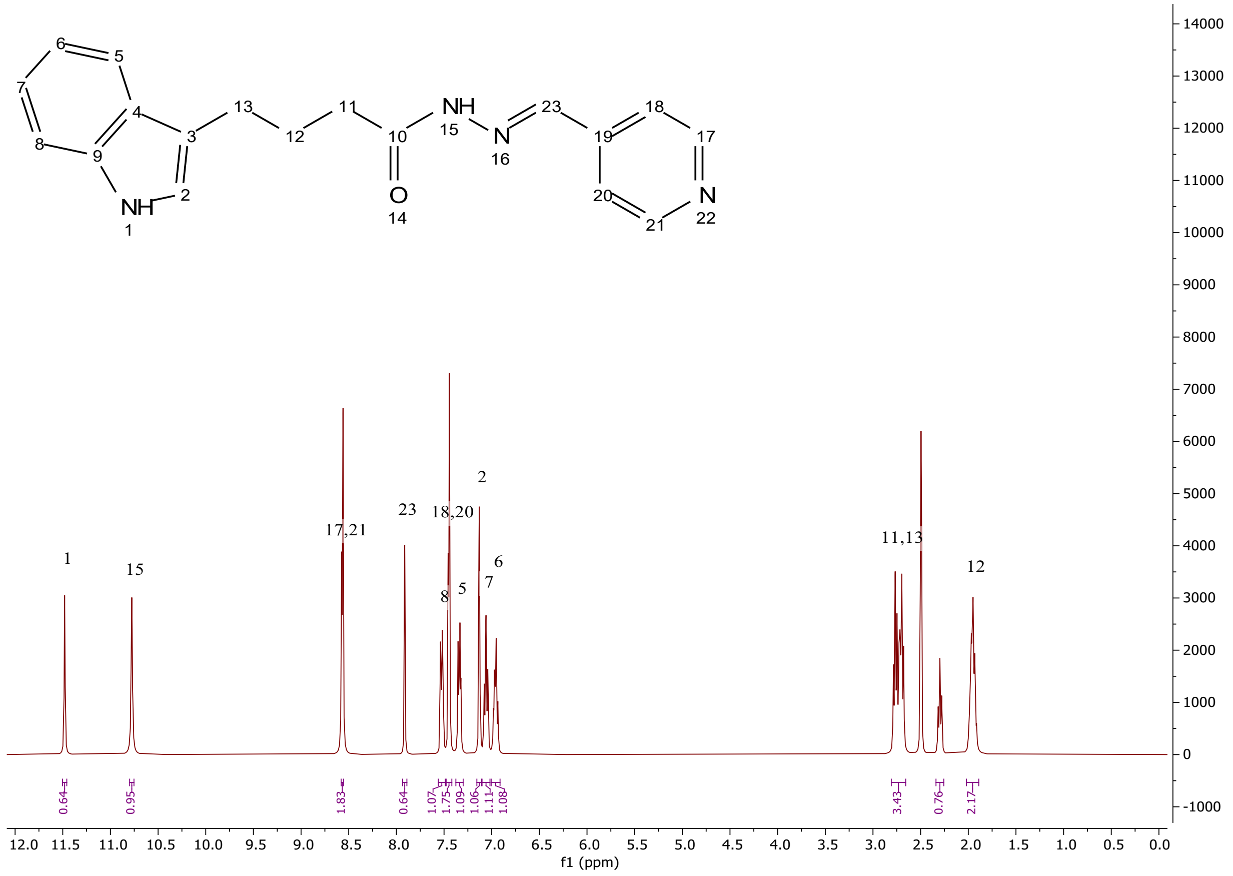

NH
1
2
3
4
5
6
7
8
9
13
12
11
10
O
14
NH
15
N
16
23
19
18
17
N
22
21
20
1
15
17,21
23
18,20
2
8
5
7
6
11,13
12
0.64
0.95
1.83
0.64
1.07
1.75
1.09
1.06
1.11
1.08
3.43
0.76
2.17
f1 (ppm)

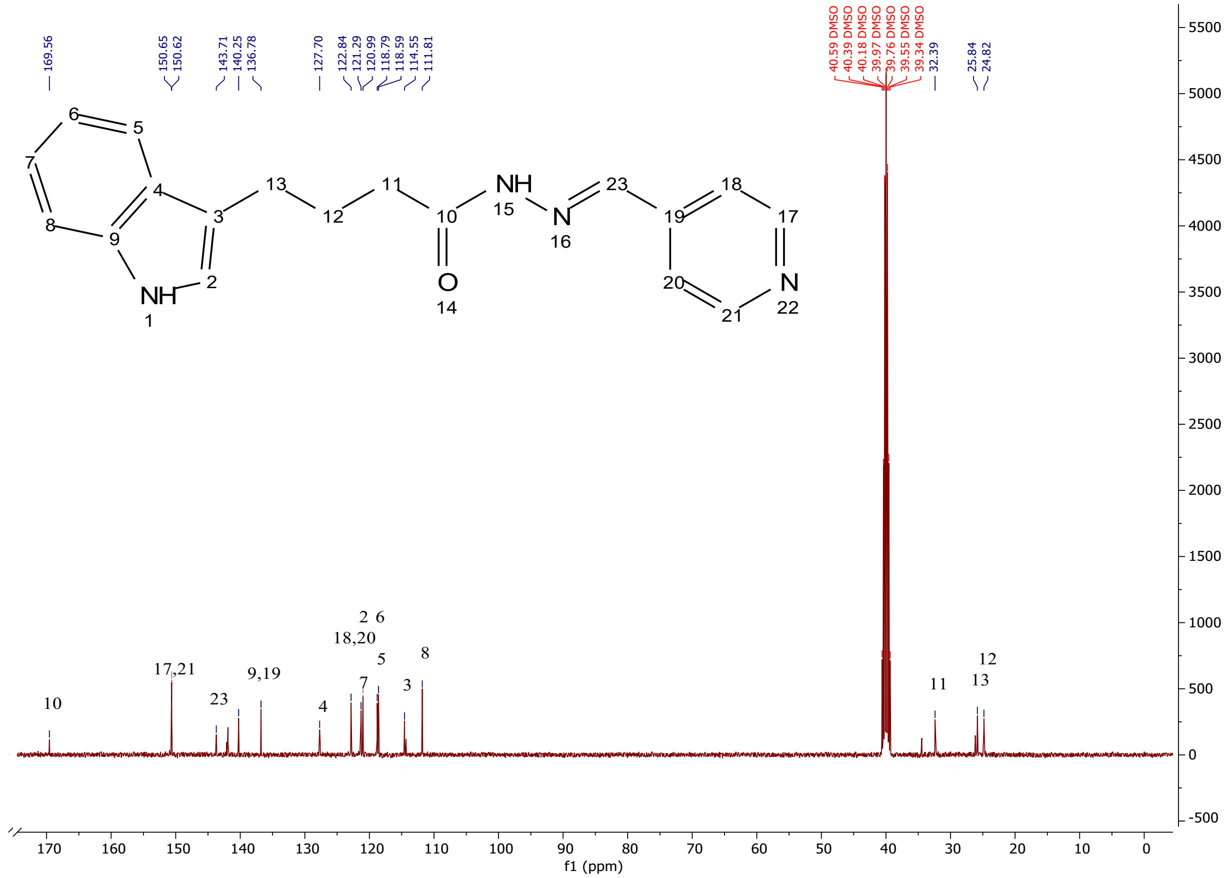
169.56
150.65
150.62
143.71
140.25
136.78
127.70
122.84
121.29
120.99
118.79
118.59
114.55
111.81
40.59 DMSO
40.39 DMSO
40.18 DMSO
39.97 DMSO
39.76 DMSO
39.55 DMSO
39.34 DMSO
32.39
25.84
24.82
10
17,21
23
9,19
4
18,20
2 6
7
5
3
8
11
12
13
f1 (ppm)

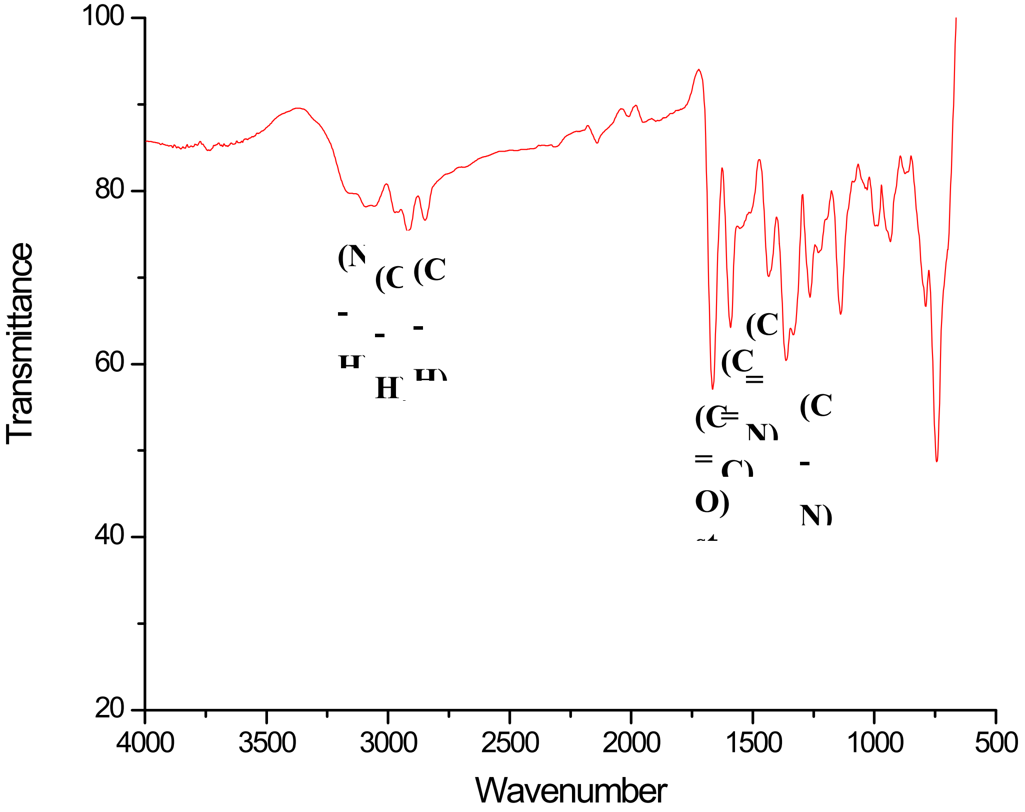

Transmittance
100
80
60
40
20
4000
3500
3000
2500
2000
1500
1000
500
Wavenumber

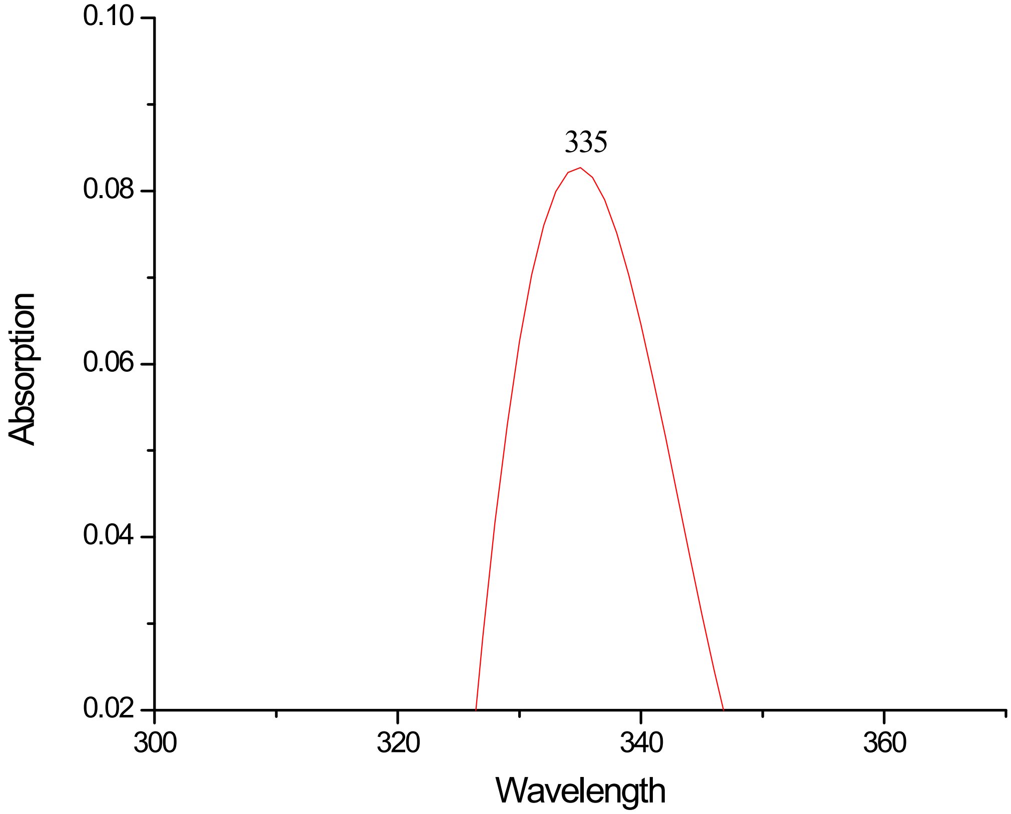

335
0.10
0.08
0.06
0.04
0.02
Absorption
300
320
340
360
Wavelength

2.2.3 *(Z)-4-(1H-indol-3-yl)-N'-(naphthalen-1-ylmethylene)butanehydrazide* (NIBH)

M.P: 132, Yield: 68%

IR $\acute{v}_{max}$(cm^{-1}): 3366 (N-H, amidic) str, 3054 (C-H, sp^2) str, 2924-2863 (C-H, sp^3) str, 1656 (-C=O, amidic), 1548 (-C=N, iminic), 1372 (C-N).

UV λ_{max}=347 nm corresponds to π→π*, n→π* transitions of the conjugated hydrazone (–C=N–NH–) system and indicating extended conjugation in the molecule.

^{1}H-NMR (400 MHz, DMSO) δ 11.27 (s, 1H, N-H^{15}, Amide), 10.78 (s, 1H, N-H^{1}), 8.60 (d, J = 8.0 Hz, 1H, Ar-H^{26}), 7.98 (dq, J = 6.3, 3.0 Hz, 2H, Ar-H^{19,24}), 7.86 (d, J = 7.2 Hz, 1H, Ar-H^{22}), 7.78 (d, J = 7.3 Hz, 1H, Ar-H^{8}), 7.68 – 7.51 (m, 4H, Ar-H^{5,20,21,25}), 7.34 (dd, J = 8.2, 4.3 Hz, 1H, Ar-H^{2}), 7.15 (s, 1H, Ar-H^{17}), 7.06 (q, J = 7.1 Hz, 1H, Ar-H^{7}), 6.95 (dt, J = 15.1, 7.4 Hz, 1H, Ar-H^{6}), 2.78 (dt, J = 15.8, 7.5 Hz, 3H, H$^{13'',11}$), 2.33 (t, J = 7.4 Hz, 1H, H$^{13'}$), 2.01 (td, J = 7.7, 3.7 Hz, 2H, H^{12}).

^{13}C-NMR (101 MHz, DMSO) δ 169.14, 142.60, 136.80, 133.98, 130.79, 130.51, 129.28, 129.21, 127.68, 127.36, 126.67, 126.03, 125.98, 124.13, 122.78, 121.27, 118.81, 118.60, 118.57, 111.81, 32.63, 25.72, 24.98.

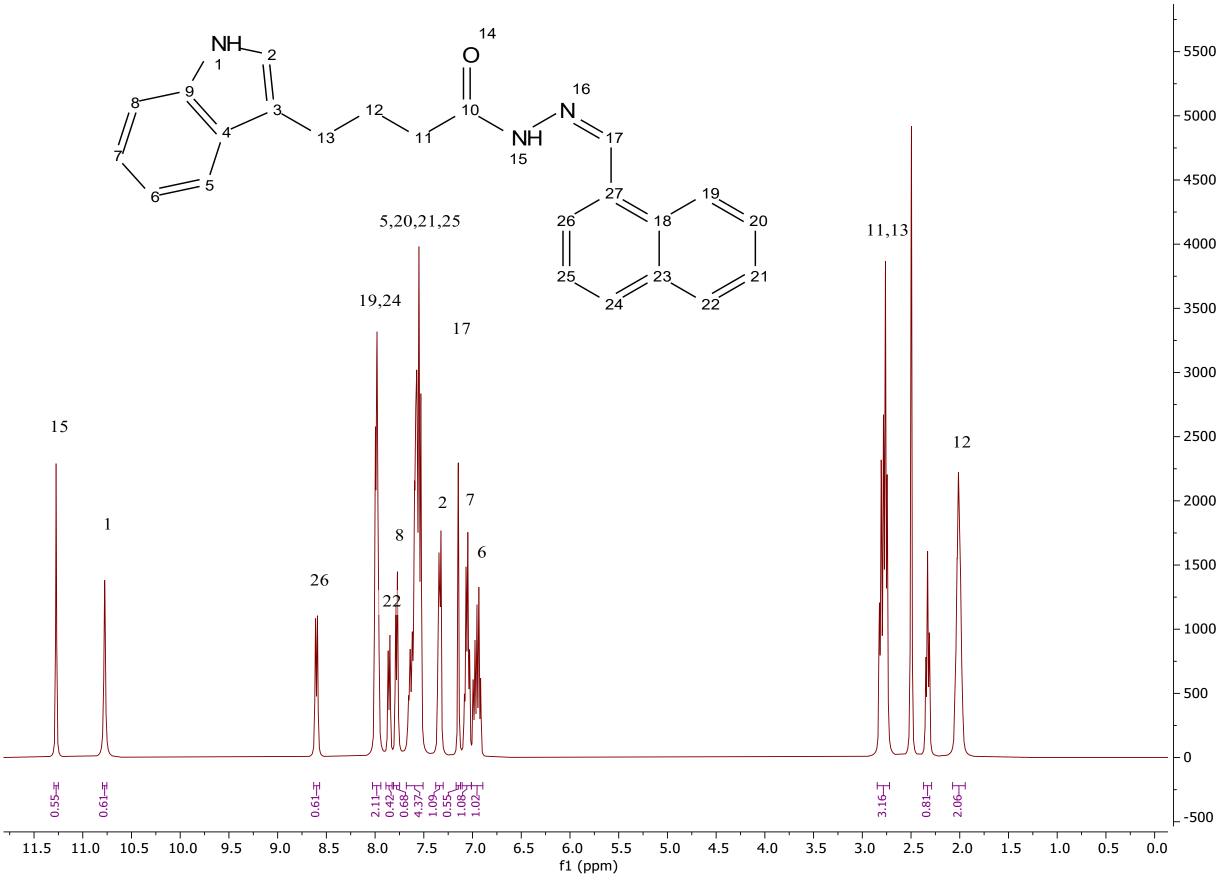

15
1
26
19,24
22
8
5,20,21,25
2
17
7
6
11,13
12
f1 (ppm)

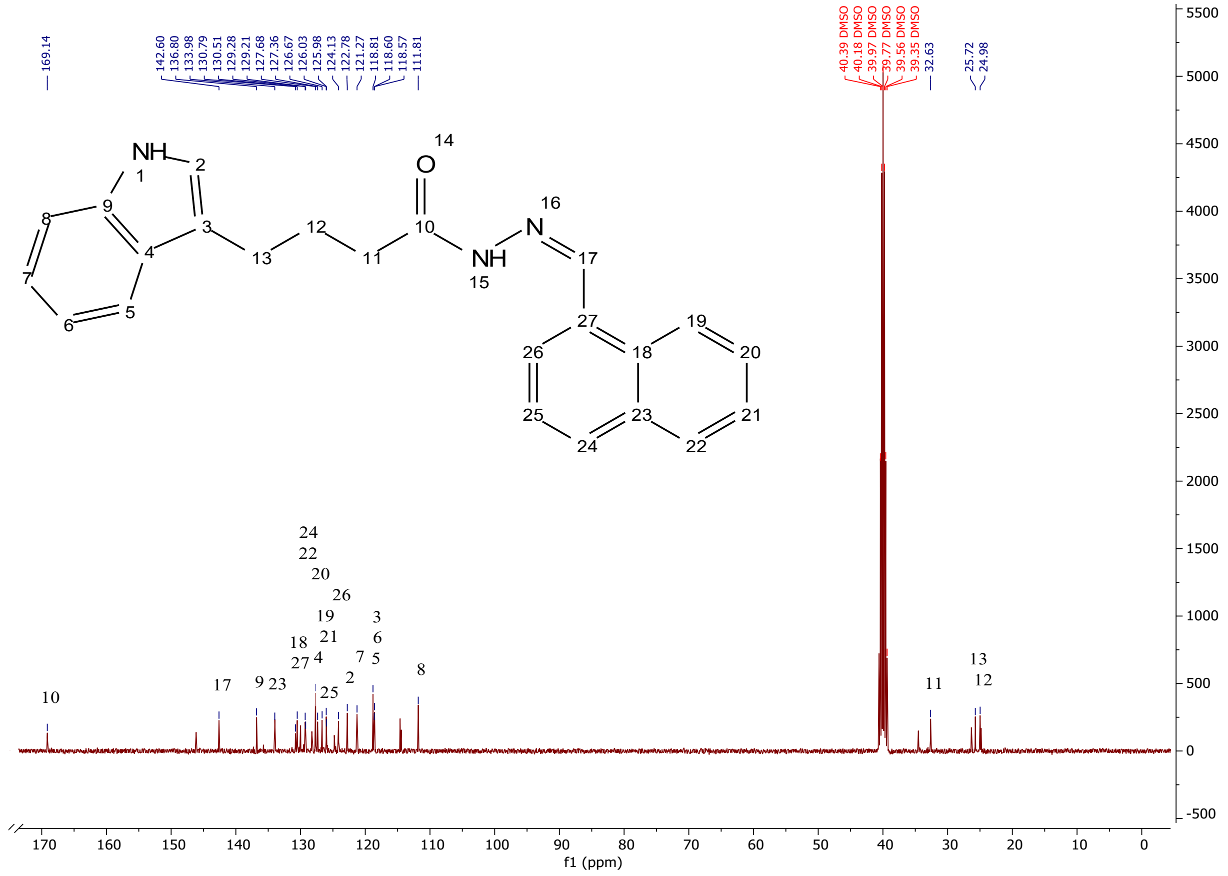

169.14
142.60
136.80
133.98
130.79
130.51
129.28
129.21
127.68
127.36
126.67
126.03
125.98
124.13
122.78
121.27
118.81
118.60
118.57
111.81
40.39 DMSO
40.18 DMSO
39.97 DMSO
39.77 DMSO
39.56 DMSO
39.35 DMSO
32.63
25.72
24.98
f1 (ppm)

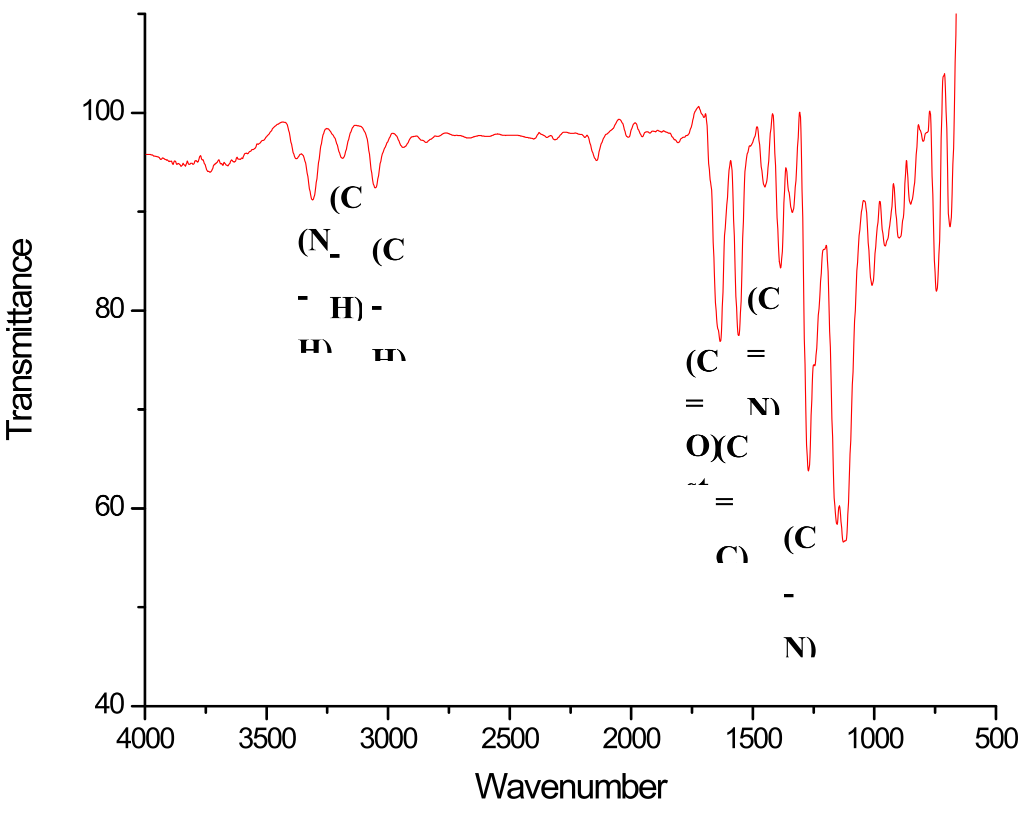

Transmittance
Wavenumber
100
80
60
40
4000
3500
3000
2500
2000
1500
1000
500
(N-H)
(C-H)
(C-H)
(C=O)
(C=C)
(C=N)
(C-N)

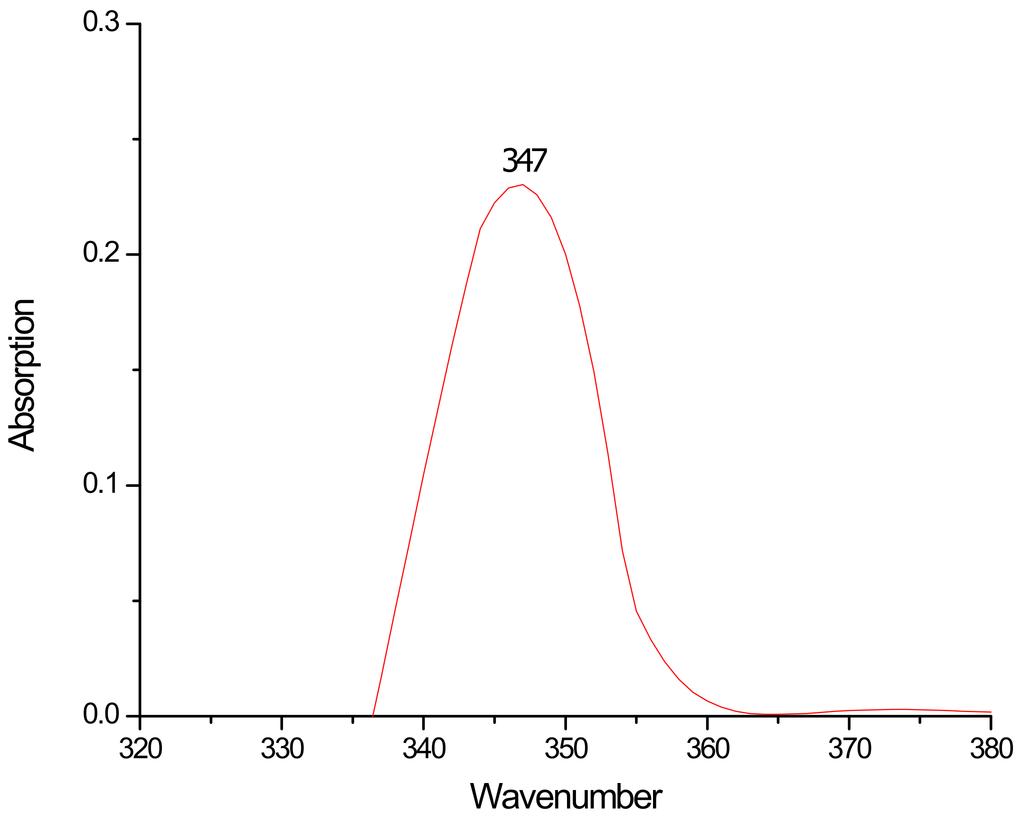

0.3
0.2
0.1
0.0
Absorption
347
320
330
340
350
360
370
380
Wavenumber

2.2.4 *(E)-4-(1H-indol-3-yl)-N'-(2-methylbenzylidene)butanehydrazide* (MBIBH)

M.P: 140, Yield: 78%

IR $\acute{\upsilon}_{max}$*(cm⁻¹)*: 3365 (N-H, amidic) str, 3042 (C-H, sp^2) str, 2915-2862 (C-H, sp^3) str, 1655 (-C=O, amidic), 1556 (-C=N, iminic), 1377 (C-N).

UV λ_{max}= 304 nm corresponds to π→π*, n→π* transitions of the conjugated hydrazone (–C=N–NH–) system and indicating extended conjugation in the molecule.

^{1}H-NMR (400 MHz, DMSO) δ 11.13 (s, 1H, N-H^{24}, Amide), 10.77 (s, 1H, N-H^{23}), 8.23 (s, 1H, Ar-H^{15}), 7.63 – 7.56 (m, 1H, Ar-H^{2}), 7.52 (d, *J* = 7.8 Hz, 1H, Ar-H^{4}), 7.37 – 7.17 (m, 4H, Ar-$H^{1,18,19,20}$), 7.12 (dd, *J* = 5.9, 2.3 Hz, 1H, Ar-H^{6}), 7.09 – 7.01 (m, 1H, Ar-H^{7}), 6.96 (q, *J* = 7.6 Hz, 1H, Ar-H^{5}), 2.80 – 2.63 (m, 4H, Ar-$H^{10'',12}$), 2.38 (d, *J* = 10.7 Hz, 3H, H^{22}), 2.26 (t, *J* = 7.4 Hz, 1H, $H^{10'}$), 1.95 (p, *J* = 7.4 Hz, 2H, H^{12}).

^{13}C-NMR (101 MHz, DMSO) δ 168.98, 144.69, 141.97, 136.78, 132.70, 131.34, 129.67, 127.68, 126.61, 126.55, 122.75, 121.26, 118.78, 118.55, 114.63, 111.79, 40.60, 40.39, 40.19, 39.98, 39.77, 39.56, 39.35, 32.51, 25.79, 24.92, 19.84.

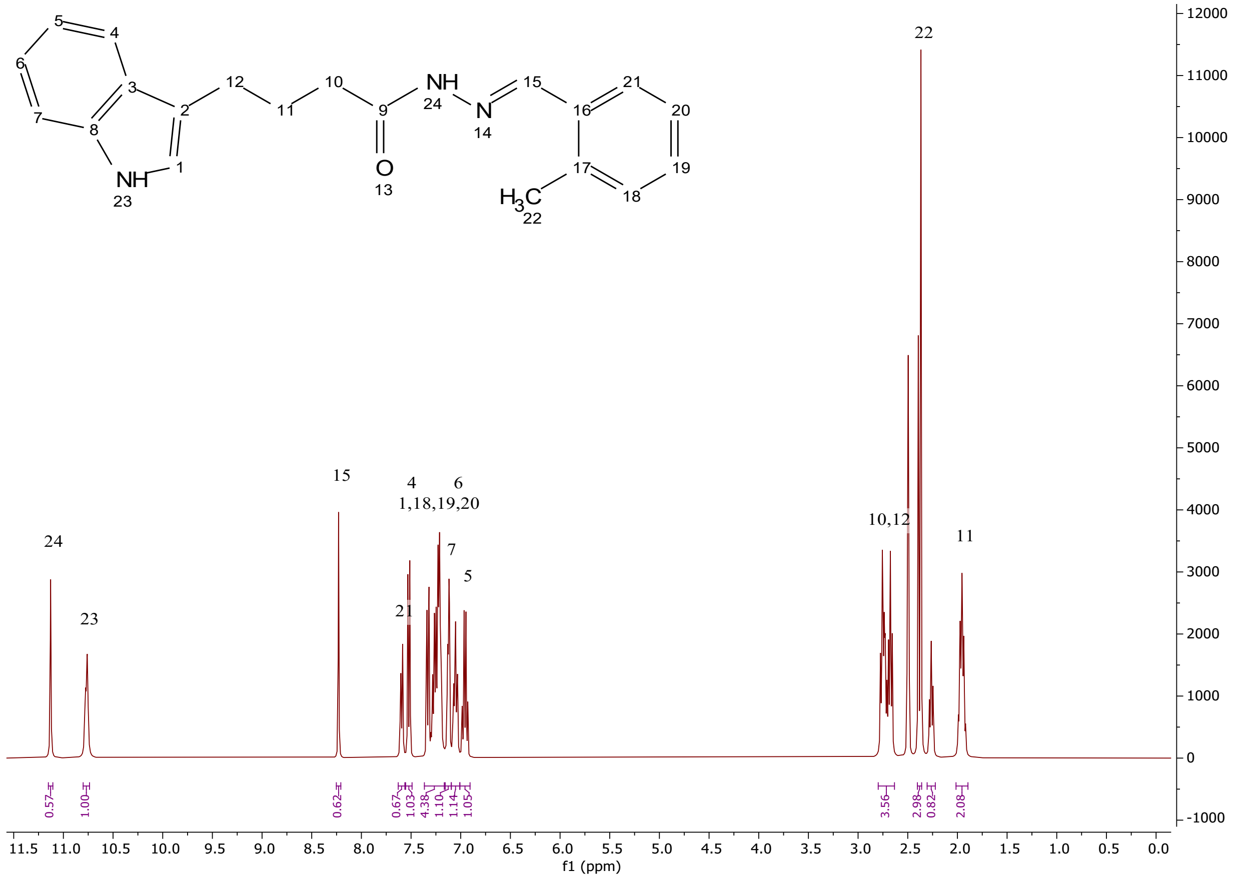

24
23
15
4
1,18,19,20
6
7
5
21
10,12
22
11
0.57
1.00
0.62
0.67
1.03
4.38
1.10
1.14
1.05
3.56
2.98
0.82
2.08
f1 (ppm)

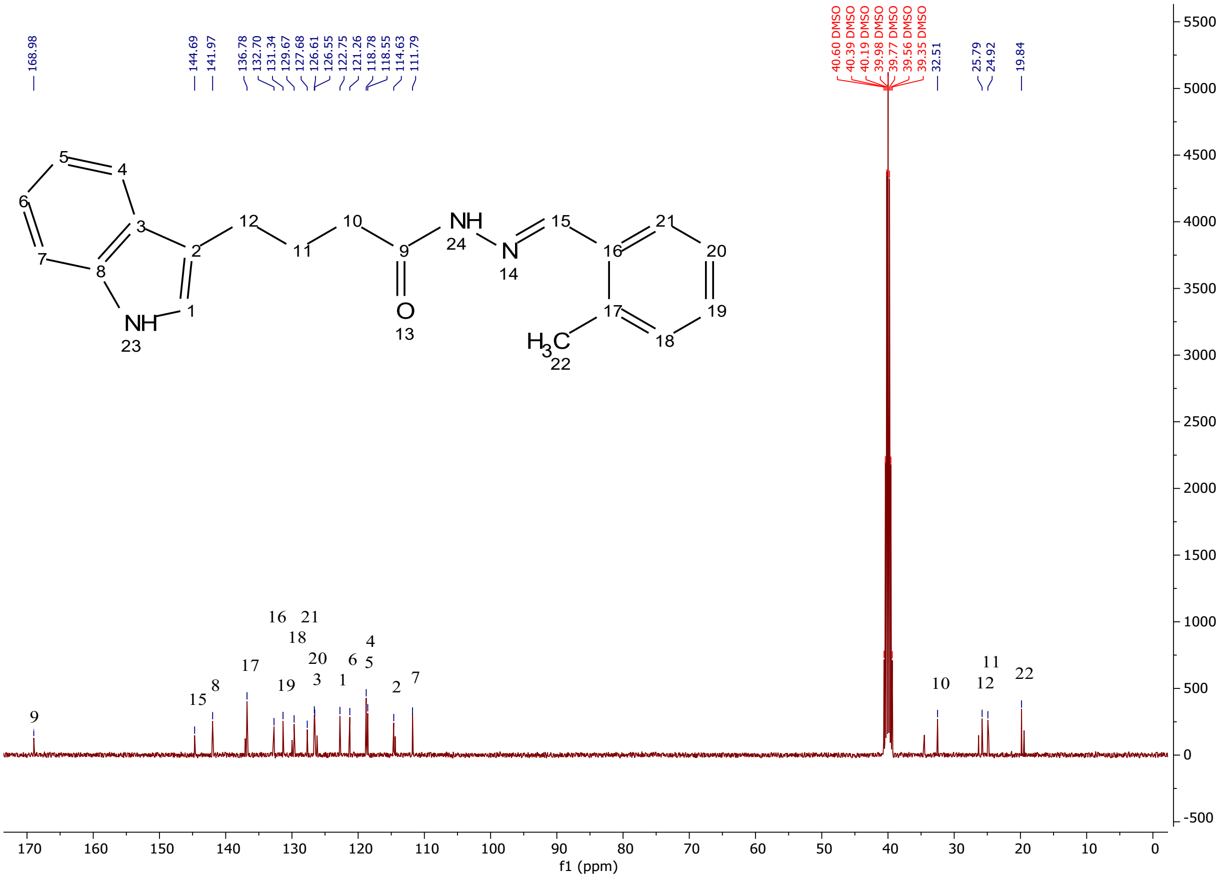

168.98
144.69
141.97
136.78
132.70
131.34
129.67
127.68
126.61
126.55
122.75
121.26
118.78
118.55
114.63
111.79
40.60 DMSO
40.39 DMSO
40.19 DMSO
39.98 DMSO
39.77 DMSO
39.56 DMSO
39.35 DMSO
32.51
25.79
24.92
19.84
NH
23
NH
24
N
14
O
13
H3C
22
f1 (ppm)

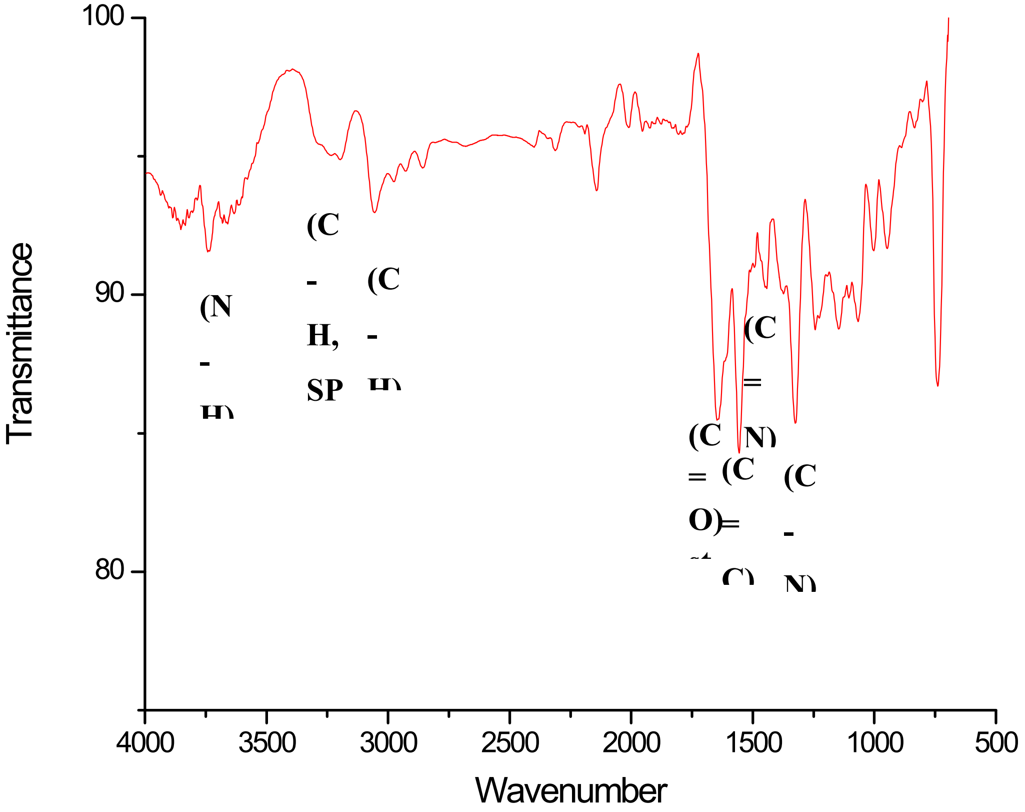

100
90
80
Transmittance
4000
3500
3000
2500
2000
1500
1000
500
Wavenumber
(N
-
(C
-
H,
SP
(C
-
(C
=
(C
=
O)
(C
=
(C
-

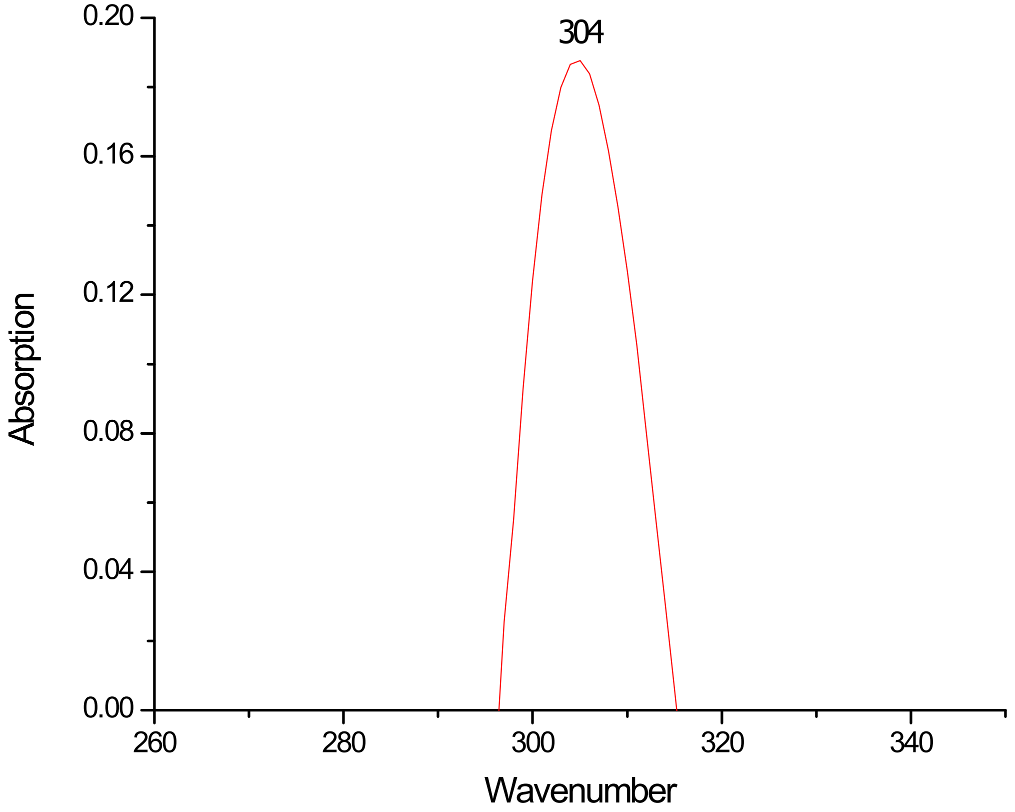

304
0.20
0.16
0.12
0.08
0.04
0.00
Absorption
260
280
300
320
340
Wavenumber

2.2.5 (E)-4-(1H-indol-3-yl)-N'-(4-methoxybenzylidene)butanehydrazide (4-MBIBH)

M.P: 118, Yield: 83%

IR $\acute{\upsilon}_{max}$ (cm^{-1}): 3299-3230 (-N–H, amidic), 3060 (Ar-H) str, 2938-2847 (C-H, sp^3) str, 1652 (-C=O, amidic)str, 1606 (C=C)str, 1542 (-C=N, iminic)str, 1246 (C-N)str.

UV λ_{max}= 389 nm corresponds to π→π*, n→π* transitions of the conjugated hydrazone (–C=N–NH–) system and indicating extended conjugation in the molecule.

^{1}H-NMR (400 MHz, DMSO) δ 11.05 (s, 1H, N-H^{1}), 10.75 (s, 1H, N-H^{15}, Amide), 7.88 (s, 1H, Ar-H^{25}), 7.64 – 7.56 (m, 1H, Ar-H^{5}), 7.55 – 7.43 (m, 2H, Ar-H^{18,22}), 7.33 (dq, J = 7.9, 1.1 Hz, 1H, Ar-H^{8}), 7.11 (d, J = 2.3 Hz, 1H, Ar-H^{2}), 7.05 (ddd, J = 8.1, 7.0, 1.2 Hz, 1H, Ar-H^{7}), 7.01 – 6.91 (m, 3H, Ar-H^{6,19,21}), 3.78 (d, J = 1.5 Hz, 3H, CH_3^{24}), 2.70 (ddd, J = 31.1, 15.1, 7.5 Hz, 3H, H$^{11'',13}$), 2.23 (t, J = 7.4 Hz, 1H, H$^{11'}$), 1.93 (p, J = 7.5 Hz, 2H, H^{12}).

^{13}C-(101 MHz, DMSO) δ 168.36, 160.38, 142.12, 136.29, 128.46, 128.07, 127.21, 126.92, 122.27, 120.77, 118.31, 118.09, 118.07, 114.23, 111.30, 55.24, 32.02, 25.35, 24.43.

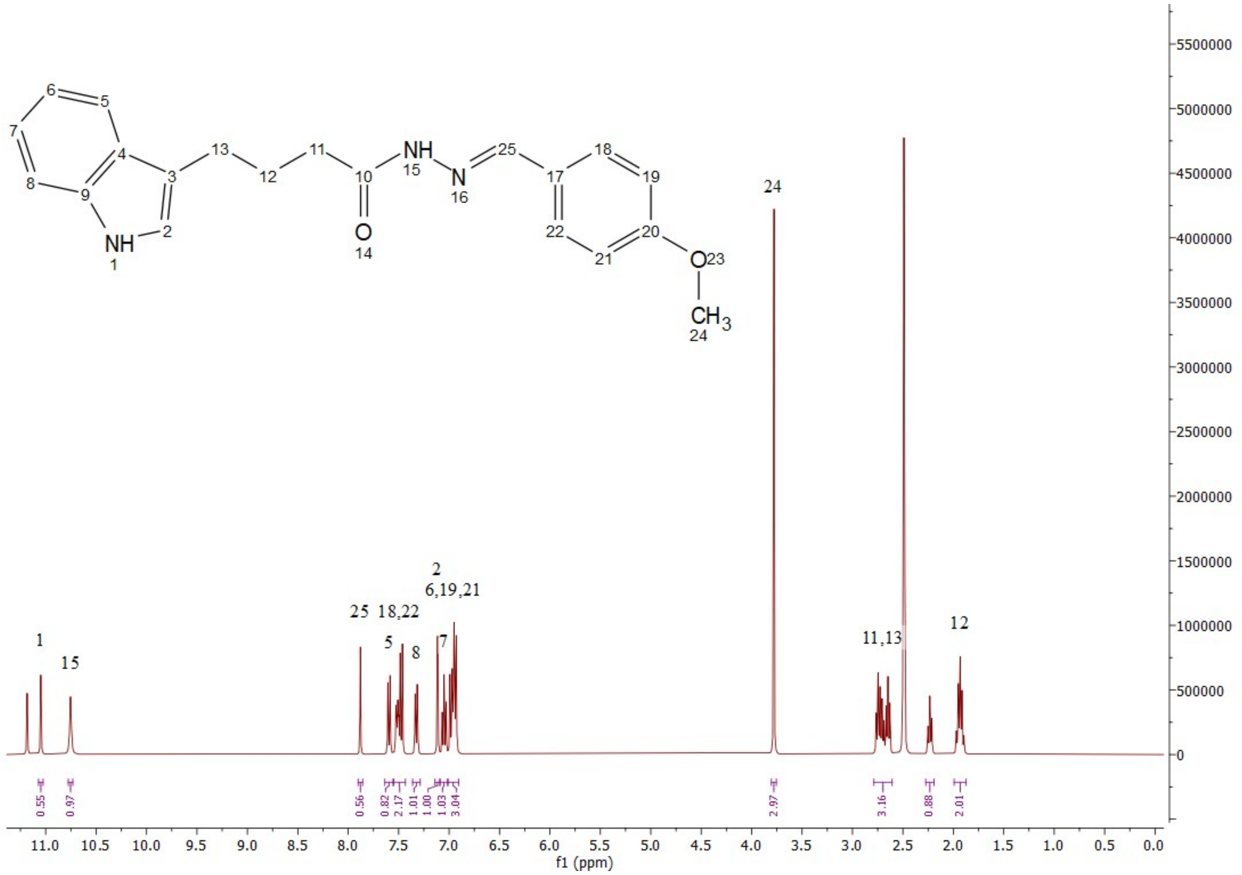

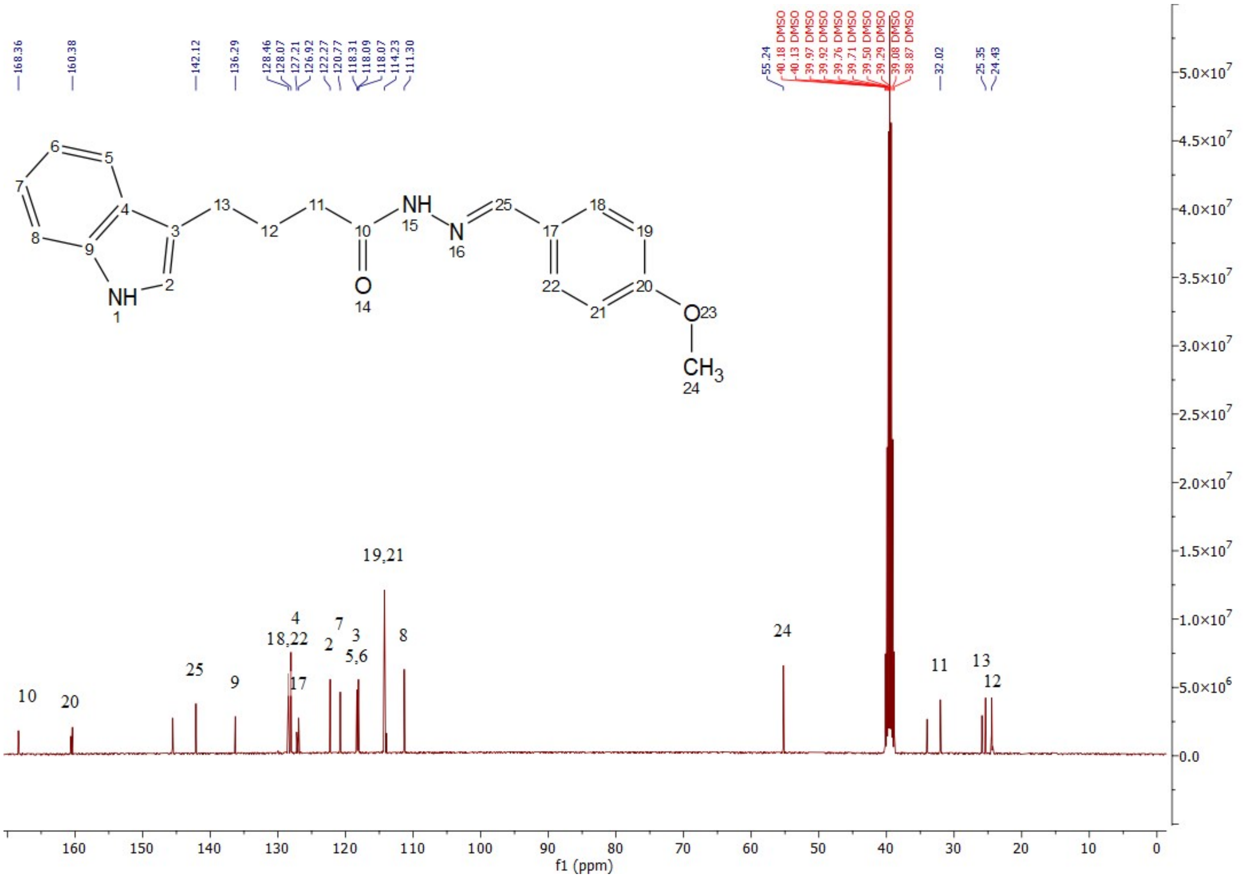
168.36
160.38
142.12
136.29
128.46
128.07
127.21
126.92
122.27
120.77
118.31
118.09
118.07
114.23
111.30
55.24
40.18 DMSO
40.13 DMSO
39.97 DMSO
39.92 DMSO
39.76 DMSO
39.71 DMSO
39.50 DMSO
39.29 DMSO
39.08 DMSO
38.87 DMSO
32.02
25.35
24.43
NH
O
CH3
10
20
25
9
4
18,22
17
2
7
3
5,6
19,21
8
24
11
13
12
f1 (ppm)

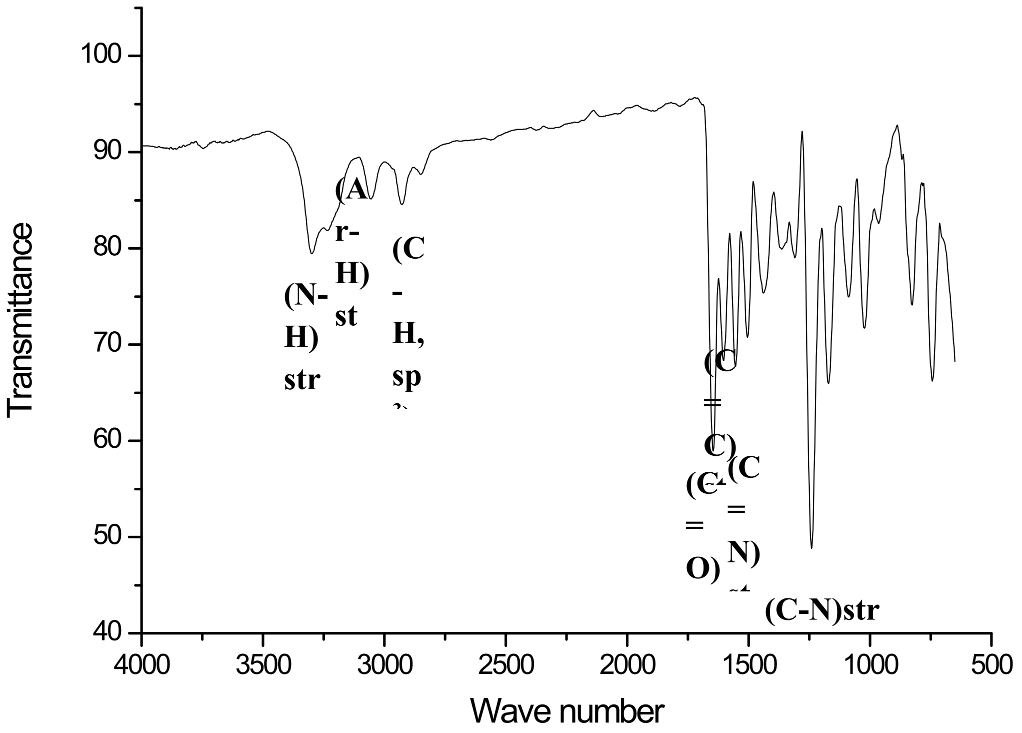
Transmittance
Wave number
100
90
80
70
60
50
40
4000
3500
3000
2500
2000
1500
1000
500
(N-H) str
(Ar-H) st
(C-H, sp
(C=C)
(C=O)
(C=N)
(C-N)str

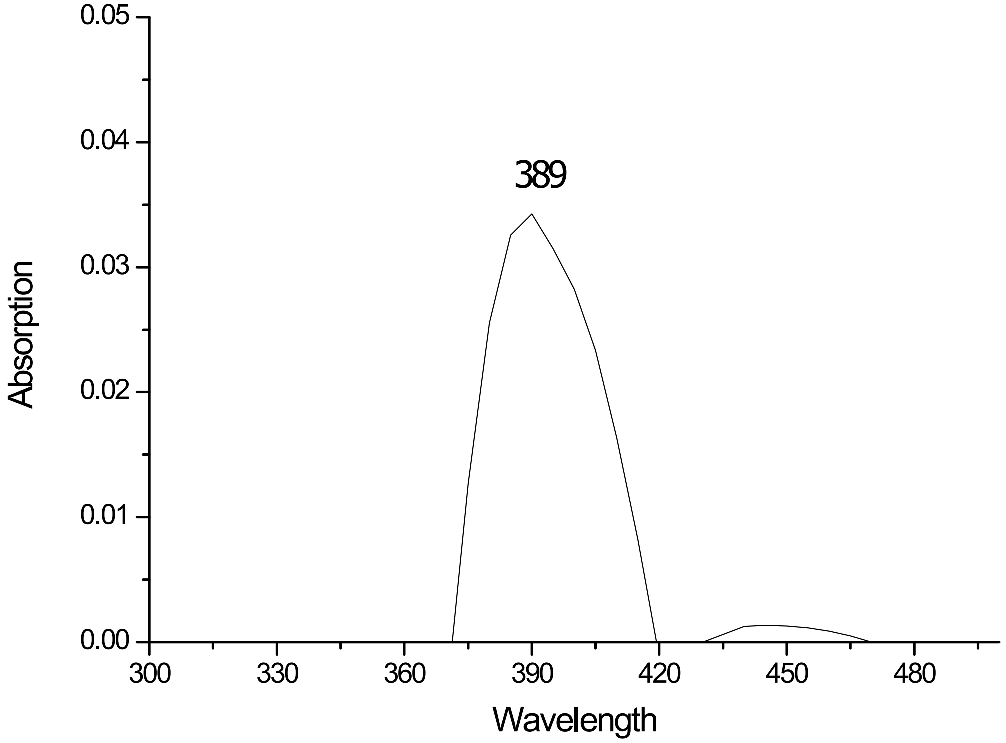
389
Absorption
0.05
0.04
0.03
0.02
0.01
0.00
300
330
360
390
420
450
480
Wavelength

2.2.6 (E)-N'-((1H-indol-5-yl)methylene)-4-(1H-indol-3-yl)butanehydrazide (ICIBH)

M.P: 126, Yield: 76%

IR $\acute{\upsilon}_{max}(cm^{-1})$: 3368 (-N–H, amidic), 3036 (Ar-H) str, 2926-2865 (C-H, sp^3) str, 1643 (-C=O, amidic)str, 1598 (C=C)str 1557 (-C=N, iminic)str, 1284 (C-N)str.

UV λ_{max}= 308 nm corresponds to π→π*, n→π* transitions of the conjugated hydrazone (–C=N–NH–) system and indicating extended conjugation in the molecule.

^{1}H-NMR (500 MHz, DMSO) δ 11.26 (s, 1H, Ar-H^{21}), 10.99 (s, 1H, Ar-H^{17}), 10.76 (s, 1H, Ar-H^{15}, Amide), 8.03 (s, 1H, Ar-H^{26}), 7.70 (s, 1H, Ar-H^{18}), 7.53 (t, J = 9.5 Hz, 2H, Ar-$H^{5,8}$), 7.42 (d, J = 9.8 Hz, 1H, Ar-H^{24}), 7.37 (s, 1H, Ar-H^{23}), 7.33 (d, J = 7.8 Hz, 1H, Ar-H^{2}), 7.13 (s, 1H, Ar-H^{21}), 7.05 (d, J = 7.8 Hz, 1H, Ar-H^{6}), 6.98 – 6.92 (m, 1H, Ar-H^{7}), 6.48 (s, 1H, Ar-H^{19}), 2.77 (t, J = 7.3 Hz, 1H, $H^{12''}$), 2.71 (d, J = 9.9 Hz, 2H, H^{13}), 2.25 (t, J = 7.3 Hz, 1H, H^{12}), 2.00 – 1.93 (m, 2H, H^{11}).

^{13}C-NMR (126 MHz, DMSO) δ 168.21, 144.35, 136.68, 136.30, 127.56, 127.23, 126.26, 125.52, 122.27, 120.75, 120.33, 118.95, 118.32, 118.05, 111.92, 111.88, 111.29, 101.82, 32.04, 25.32, 24.48.

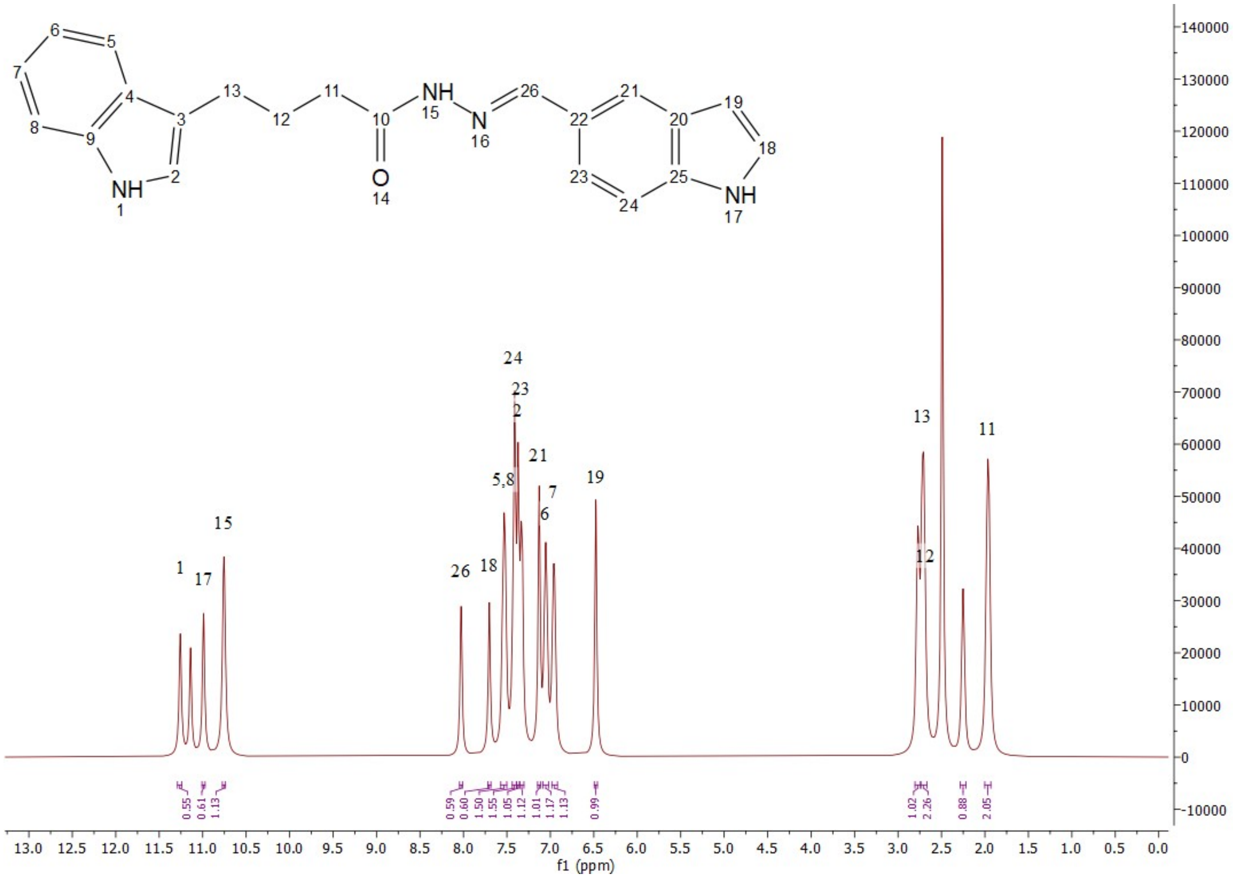

NH
1
2
3
4
5
6
7
8
9
13
12
11
10
O
14
NH
15
N
16
26
22
21
20
19
18
NH
17
23
24
25
24
23
2
21
5,8
7
6
19
15
1
17
26
18
13
12
11
0.55
0.61
1.13
0.59
0.60
1.50
1.55
1.05
1.12
1.01
1.17
1.13
0.99
1.02
2.26
0.88
2.05
140000
130000
120000
110000
100000
90000
80000
70000
60000
50000
40000
30000
20000
10000
0
-10000
13.0
12.5
12.0
11.5
11.0
10.5
10.0
9.5
9.0
8.5
8.0
7.5
7.0
6.5
6.0
5.5
5.0
4.5
4.0
3.5
3.0
2.5
2.0
1.5
1.0
0.5
0.0
f1 (ppm)

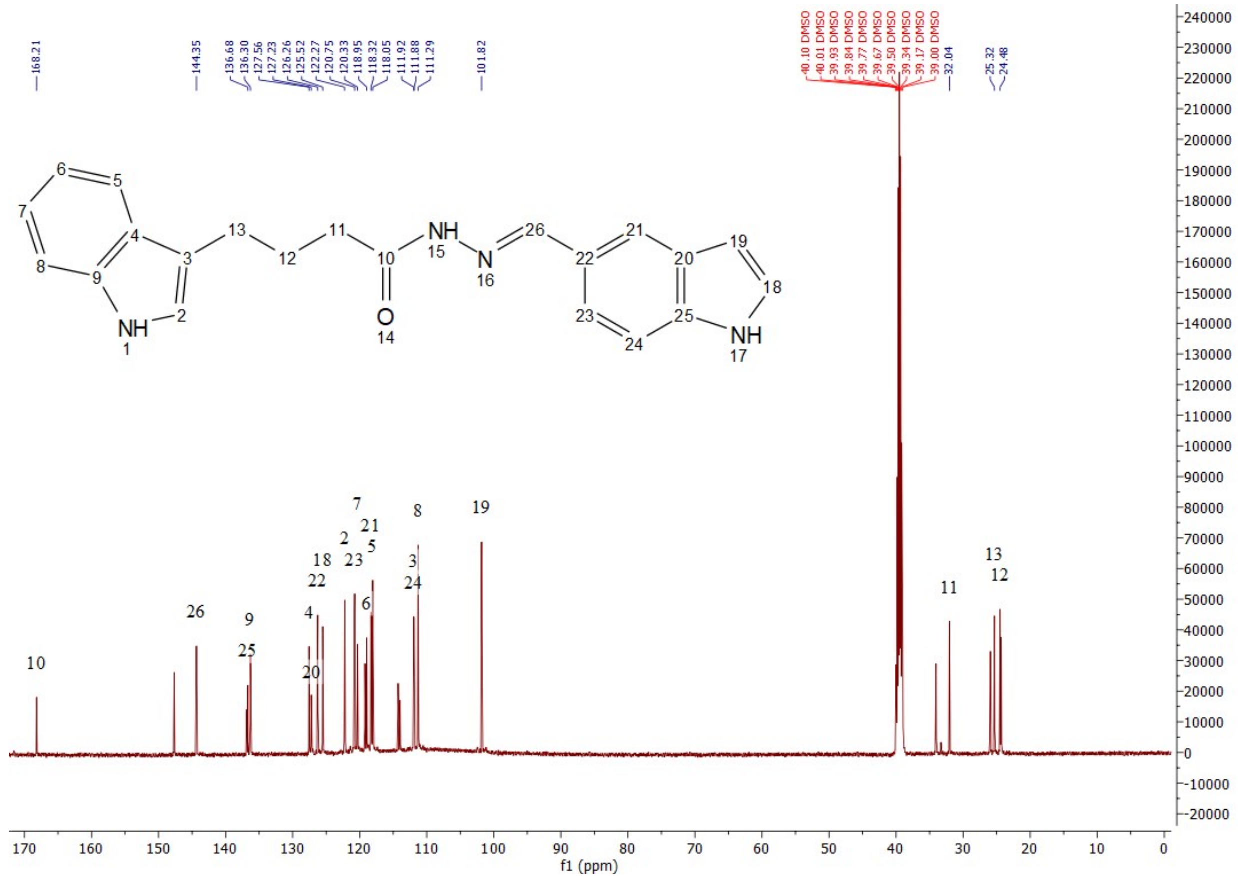

168.21
144.35
136.68
136.30
127.56
127.23
126.26
125.52
122.27
120.75
120.33
118.95
118.32
118.05
111.92
111.88
111.29
101.82
40.10 DMSO
40.01 DMSO
39.93 DMSO
39.84 DMSO
39.77 DMSO
39.67 DMSO
39.50 DMSO
39.34 DMSO
39.17 DMSO
39.00 DMSO
32.04
25.32
24.48
f1 (ppm)

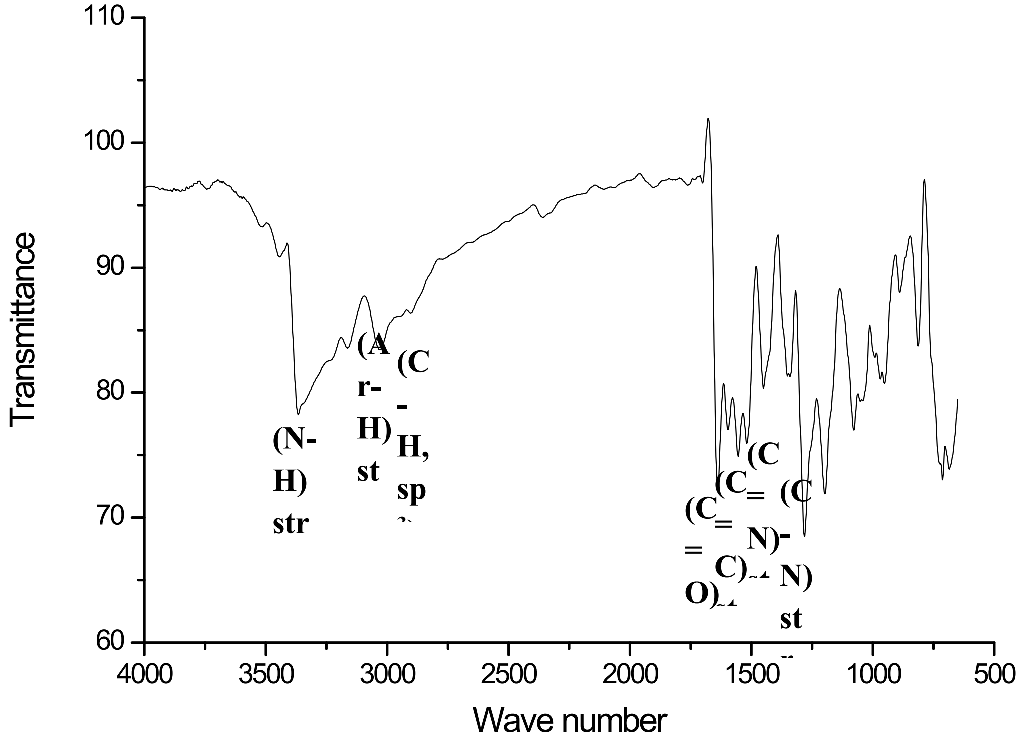
Transmittance
Wave number
110
100
90
80
70
60
4000
3500
3000
2500
2000
1500
1000
500
(N-H) str
(Ar-H) st
(C-H, sp
(C=O)
(C=C)
(C=N)
(C-N) st

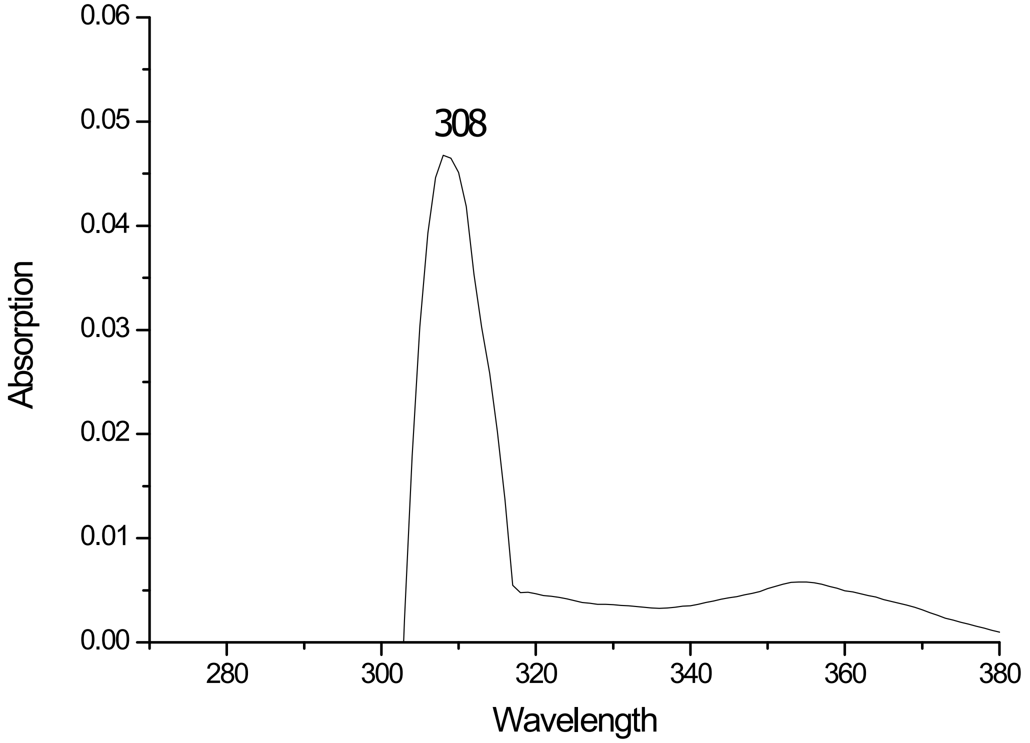
308
0.06
0.05
0.04
0.03
0.02
0.01
0.00
Absorption
280
300
320
340
360
380
Wavelength

2.2.7 (E)-4-(1H-indol-3-yl)-N'-(4-phenylbutan-2-ylidene)butanehydrazide (4-PCIBH)

M.P: 138, Yield: 64%

IR $\acute{\upsilon}_{max}$$(cm^{-1})$: 3363 (-N–H, amidic)str, 3036 (Ar-H)str, 2946-2865 (C-H, sp^3)str, 1646 (-C=O, amidic)str, 1599 (C=C)str, 1565 (-C=N, iminic)str, 1278 (C-N)str.

UV λ_{max}= 309 nm corresponds to π→π*, n→π* transitions of the conjugated hydrazone (–C=N–NH–) system and indicating extended conjugation in the molecule.

^{1}H-NMR (500 MHz, DMSO) δ 10.74 (s, 1H, N-H^{15}, Amide), 9.91 (s, 1H, N-H^{1}), 7.52 – 7.48 (m, 1H, Ar-H^{5}), 7.29 – 7.21 (m, 5H, Ar-$H^{22,23,24,25,26}$), 7.18 – 7.13 (m, 2H, Ar-$H^{7,8}$), 7.10 – 7.04 (m, 2H, Ar-$H^{2,6}$), 2.77 (dt, *J* = 12.7, 7.7 Hz, 2H, H^{13}), 2.68 (d, *J* = 7.5 Hz, 2H, H^{12}), 2.53 (d, *J* = 7.5 Hz, 1H, $H^{11''}$), 2.47 – 2.43 (m, 1H, $H^{11'}$), 1.93 – 1.83 (m, 4H, $H^{12,18}$), 1.82 (s, 3H, CH_3^{19}).

^{13}C-NMR (126 MHz, DMSO) δ 168.57, 151.19, 141.37, 136.28, 128.24, 128.13, 127.18, 125.79, 125.69, 122.14, 120.77, 120.74, 118.25, 118.01, 111.27, 33.72, 32.19, 31.54, 26.03, 24.43, 15.99.

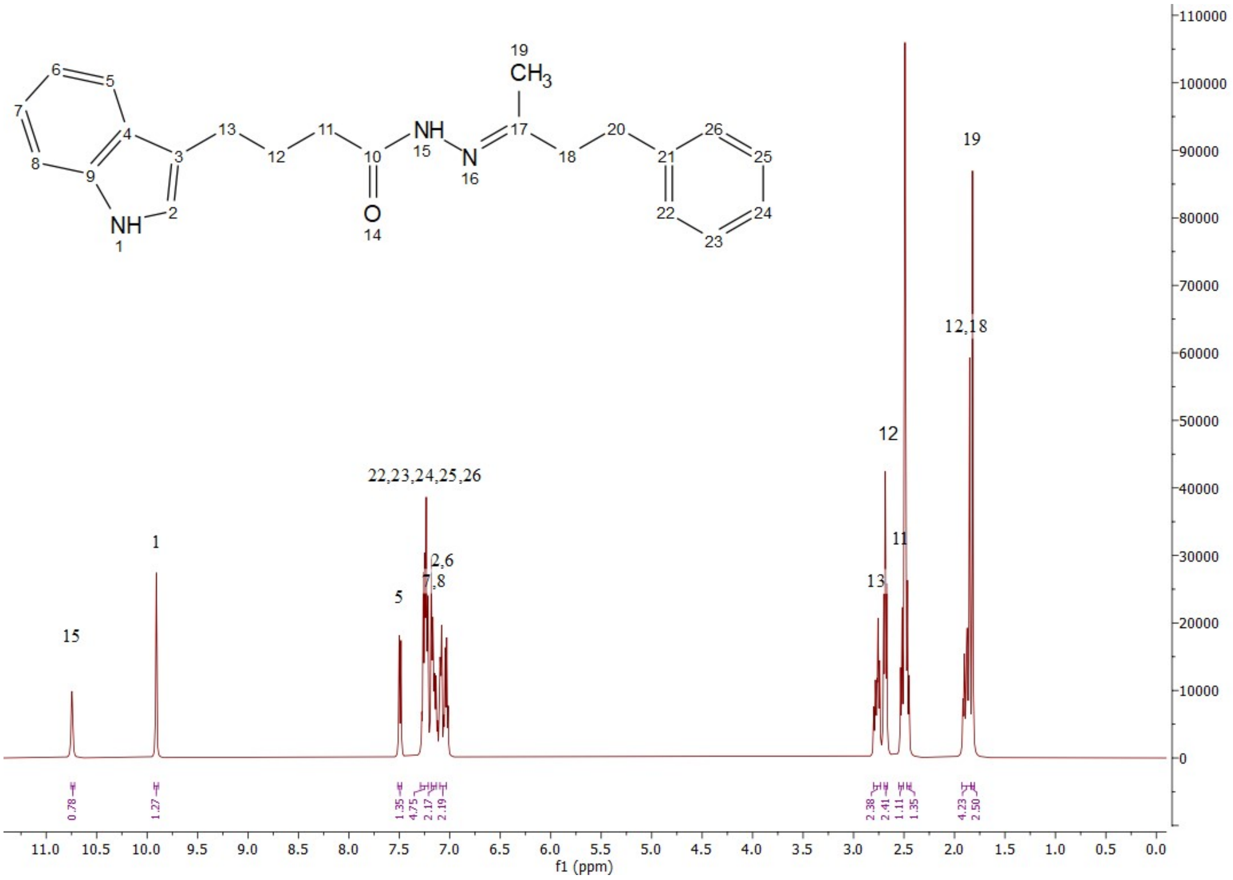

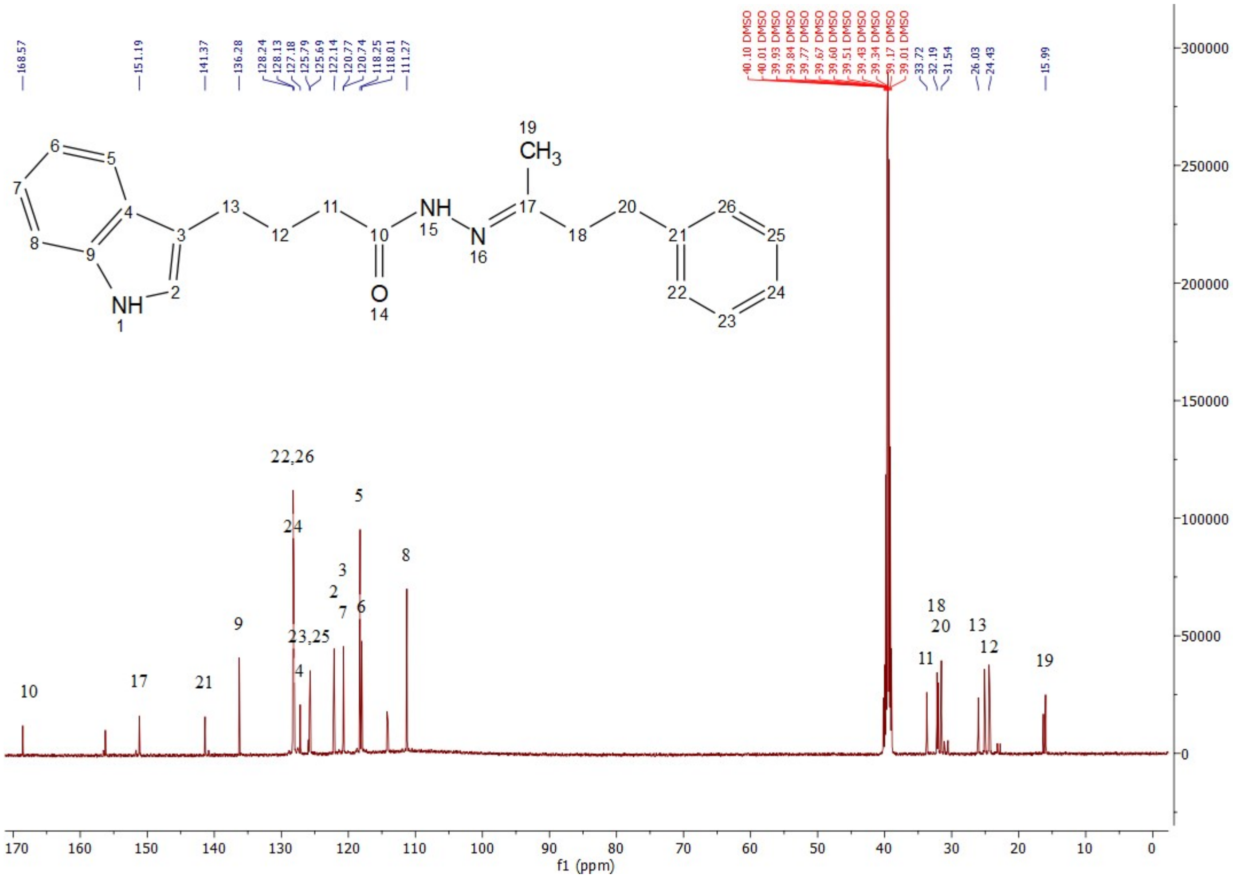

168.57
151.19
141.37
136.28
128.24
128.13
127.18
125.79
125.69
122.14
120.77
120.74
118.25
118.01
111.27
40.10 DMSO
40.01 DMSO
39.93 DMSO
39.84 DMSO
39.77 DMSO
39.67 DMSO
39.60 DMSO
39.51 DMSO
39.43 DMSO
39.34 DMSO
39.17 DMSO
39.01 DMSO
33.72
32.19
31.54
26.03
24.43
15.99
f1 (ppm)

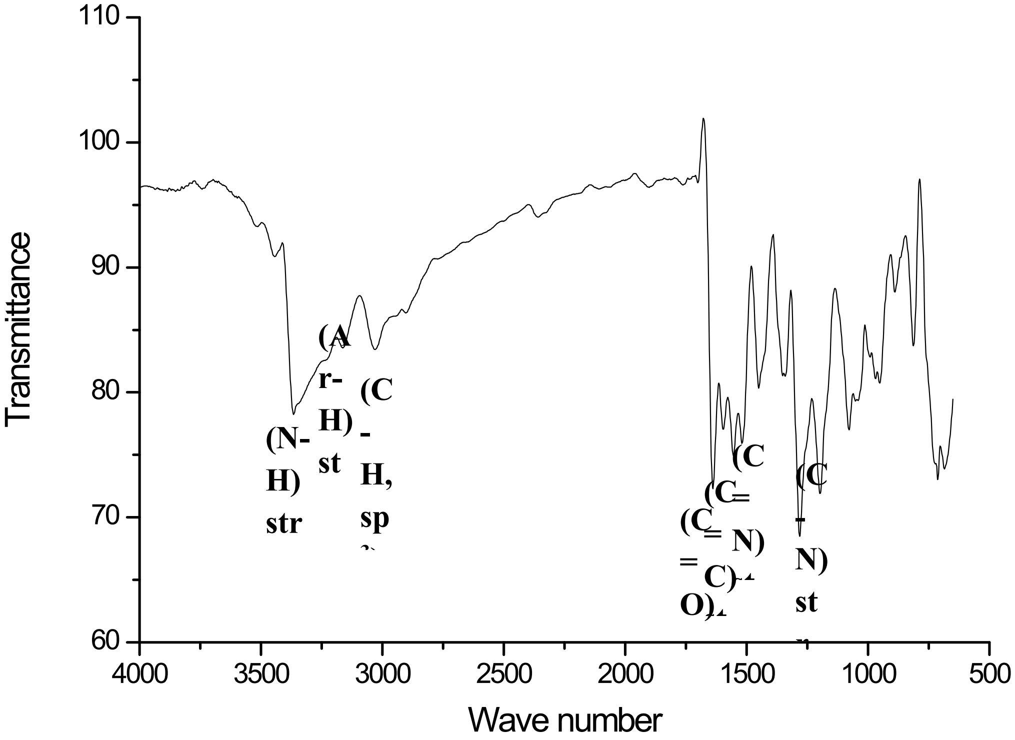

Transmittance
Wave number
110
100
90
80
70
60
4000
3500
3000
2500
2000
1500
1000
500
(N-H) str
(Ar-H) st
(C-H, sp
(C=O)
(C=C)
(C=N)
(C-N) st

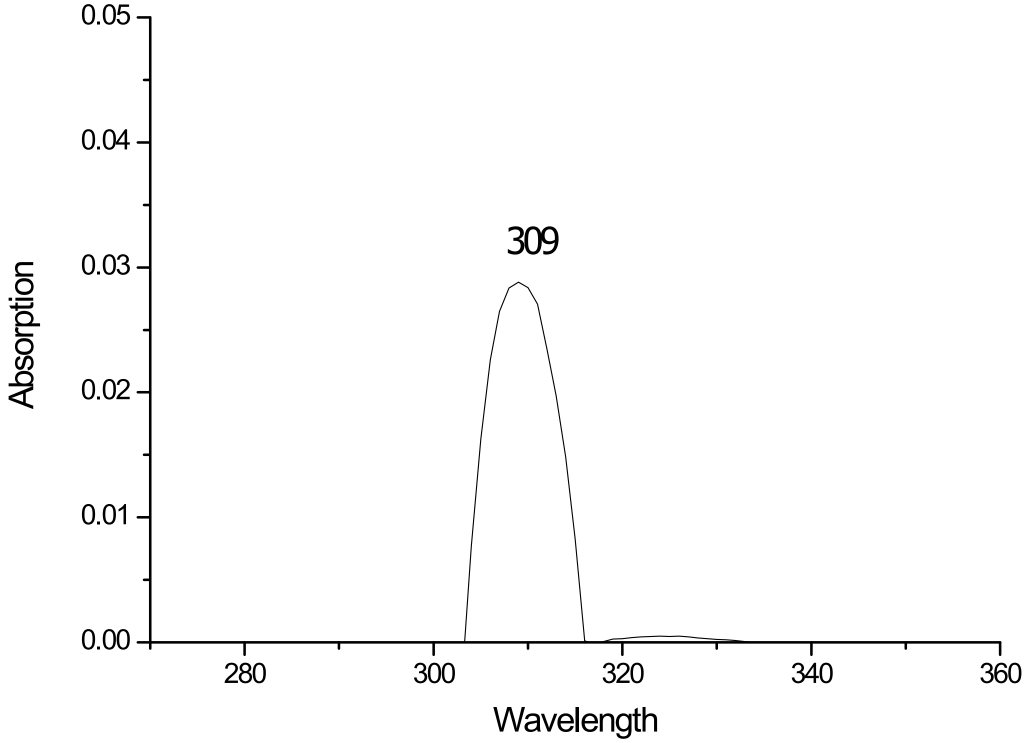

309
Absorption
0.05
0.04
0.03
0.02
0.01
0.00
280
300
320
340
360
Wavelength

2.2.8 4-(1H-indol-3-yl)-N'-((1E,2E)-3-phenylallylidene)butanehydrazide (TCIBH)

M.P: 116, Yield: 62%

IR $\acute{\upsilon}_{max}$(cm^{-1}): 3305 (-N–H, amidic)str,3160 (C-H, sp^2)str, 3041 (Ar-H)str, 2921-2865 (C-H, sp^3)str, 1643 (-C=O, amidic)str, 1544 (C=C)str, 1486 (-C=N, iminic)str, 1202 (C-N)str.

UV λ_{max}= 332 nm corresponds to π→π*, n→π* transitions of the conjugated hydrazone (–C=N–NH–) system and indicating extended conjugation in the molecule.

^{1}H-NMR (400 MHz, DMSO) δ 11.12 (s, 1H, N-H^{25}), 10.76 (s, 1H, N-H^{1}), 7.79 (d, *J* = 8.4 Hz, 1H, Ar-H^{5}), 7.58 (dt, *J* = 8.2, 2.5 Hz, 2H, Ar-$H^{20,24}$), 7.52 (dd, *J* = 7.9, 3.4 Hz, 1H, Ar-H^{16}), 7.40 – 7.27 (m, 4H, Ar-$H^{6,8,18,22}$), 7.12 (d, *J* = 2.3 Hz, 1H, Ar-H^{2}), 7.06 (t, *J* = 7.5 Hz, 1H, Ar-H^{7}), 7.00 – 6.82 (m, 3H, Ar-$H^{21,23,27}$), 2.72 (q, *J* = 7.9 Hz, 2H, H^{13}), 2.60 (t, *J* = 7.4 Hz, 1H, $H^{11''}$), 2.24 (t, *J* = 7.4 Hz, 1H, $H^{11'}$), 1.94 (p, *J* = 7.4 Hz, 2H, H^{12}).

^{13}C-NMR (101 MHz, DMSO) δ 168.98, 138.54, 136.80, 136.41, 129.28, 129.24, 129.18, 129.11, 127.64, 127.44, 122.72, 121.29, 118.82, 118.78, 118.55, 111.80, 32.13, 26.25, 24.87.

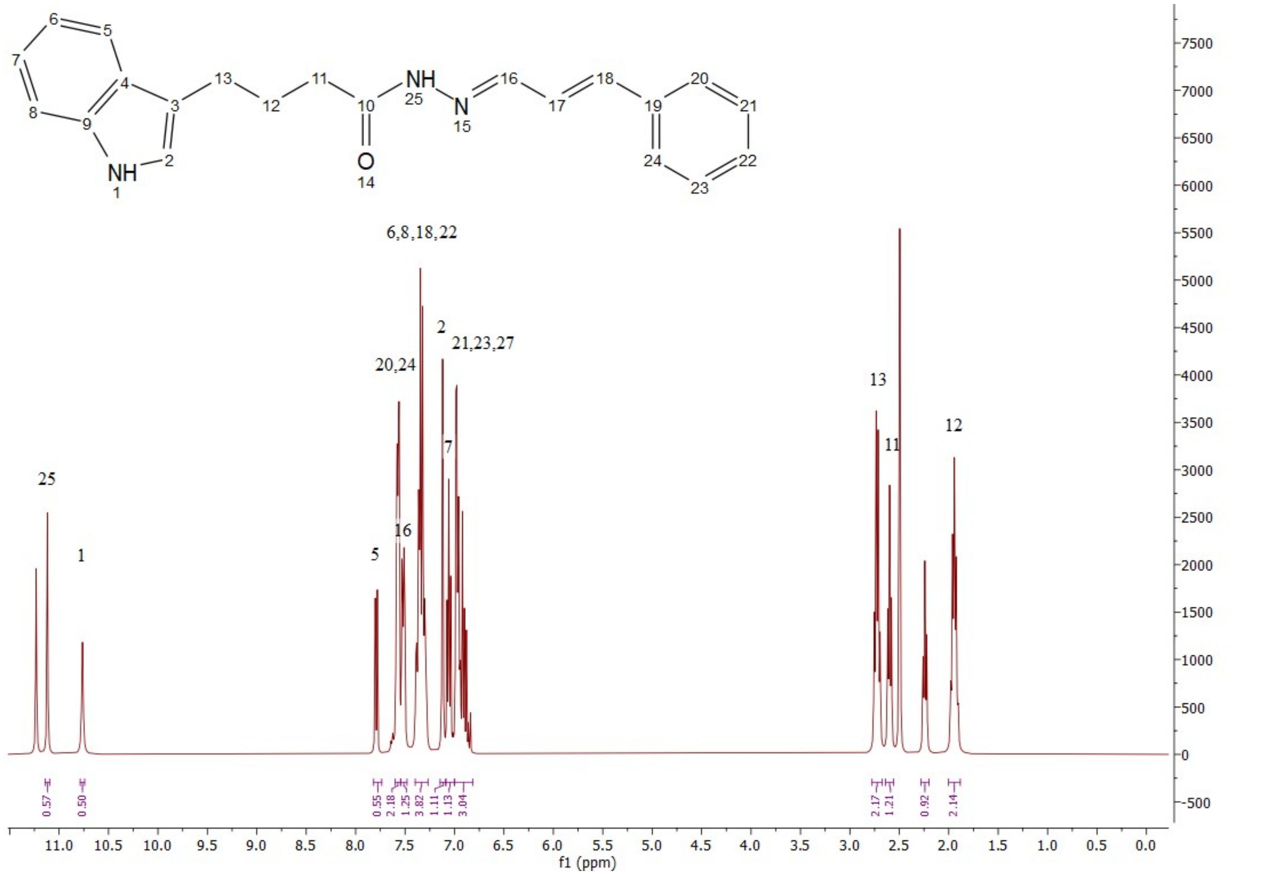

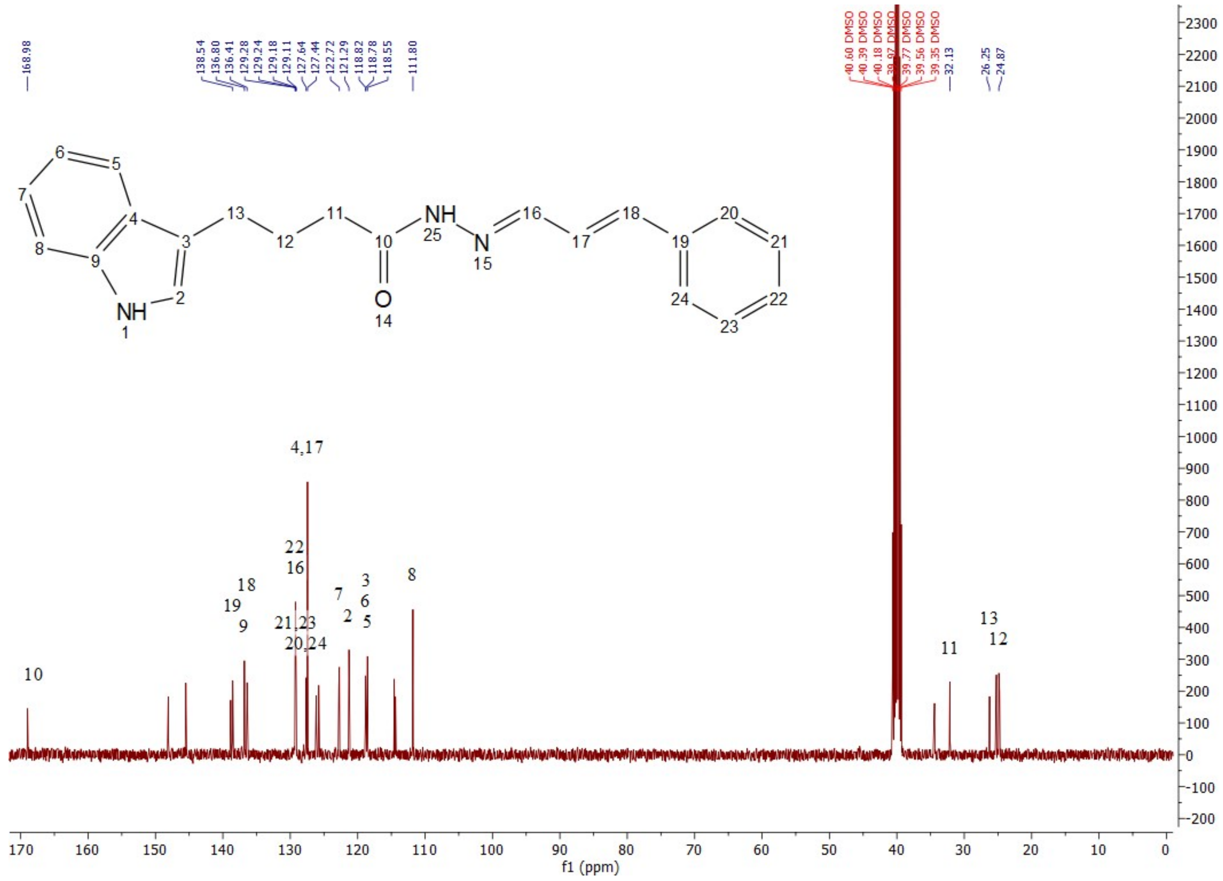

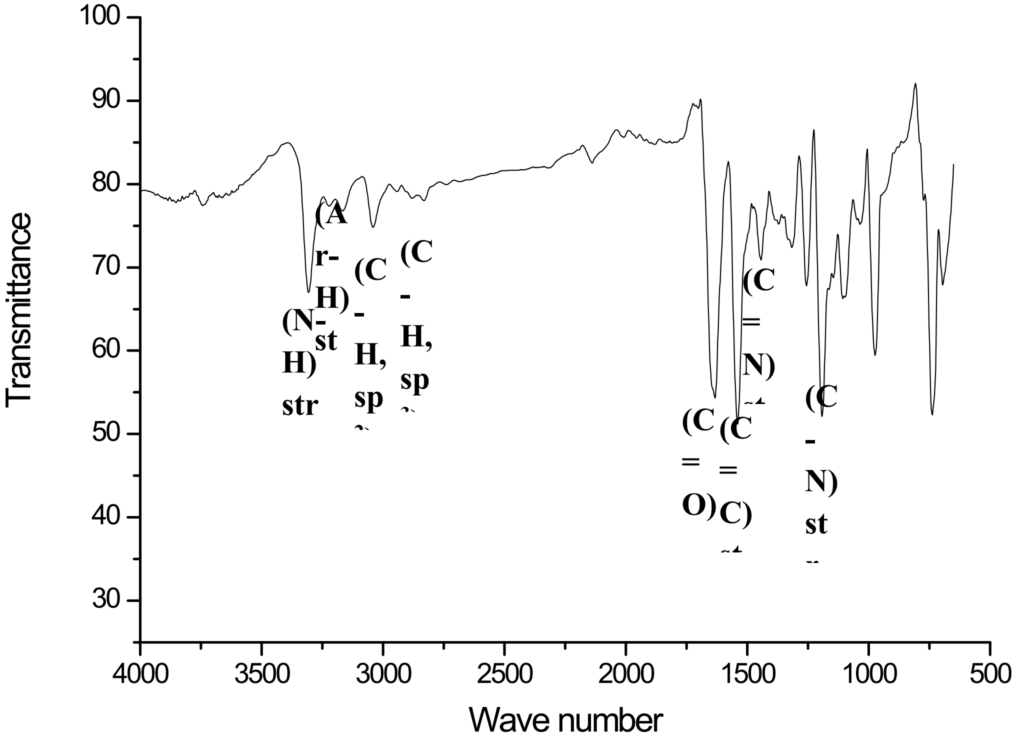
Transmittance
Wave number
100
90
80
70
60
50
40
30
4000
3500
3000
2500
2000
1500
1000
500
(N-H) str
(Ar-H) st
(C-H, sp
(C-H, sp
(C=O)
(C=C)
(C=N)
(C-N) st

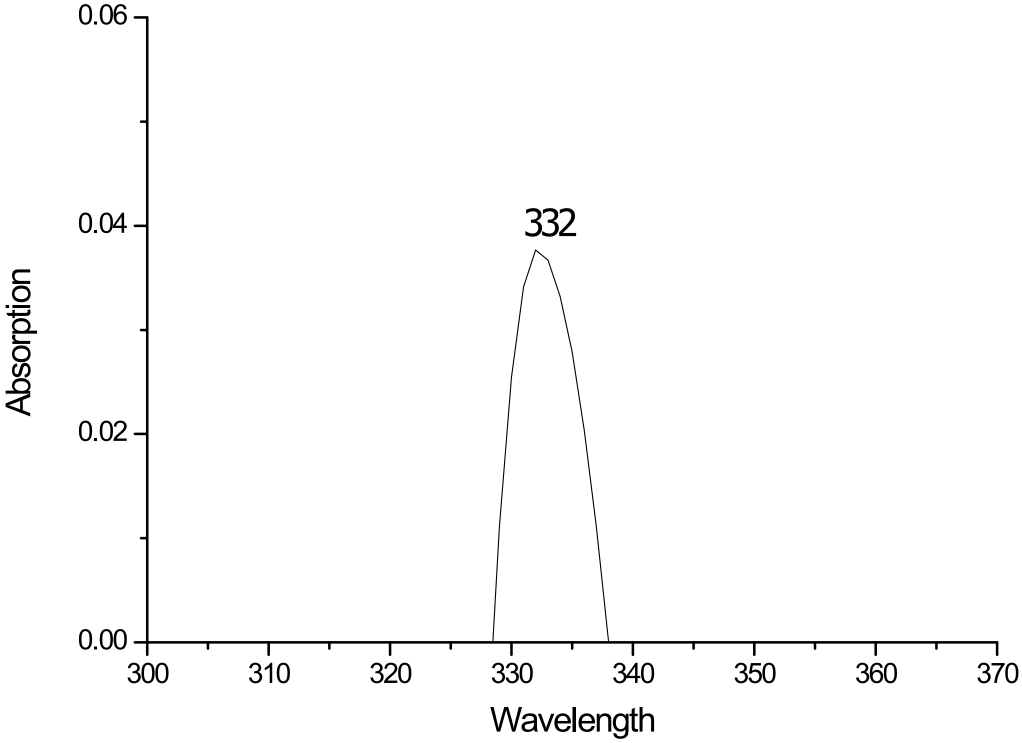

Correlation 1: HOMO-LUMO gap vs DPPH scavenging activity

Smaller energy gap → greater electronic softness → higher antioxidant activity

Regression equation: Y = 29.114X + (-57.028) | R^2 = 0.746 | r = 0.864 | n = 8

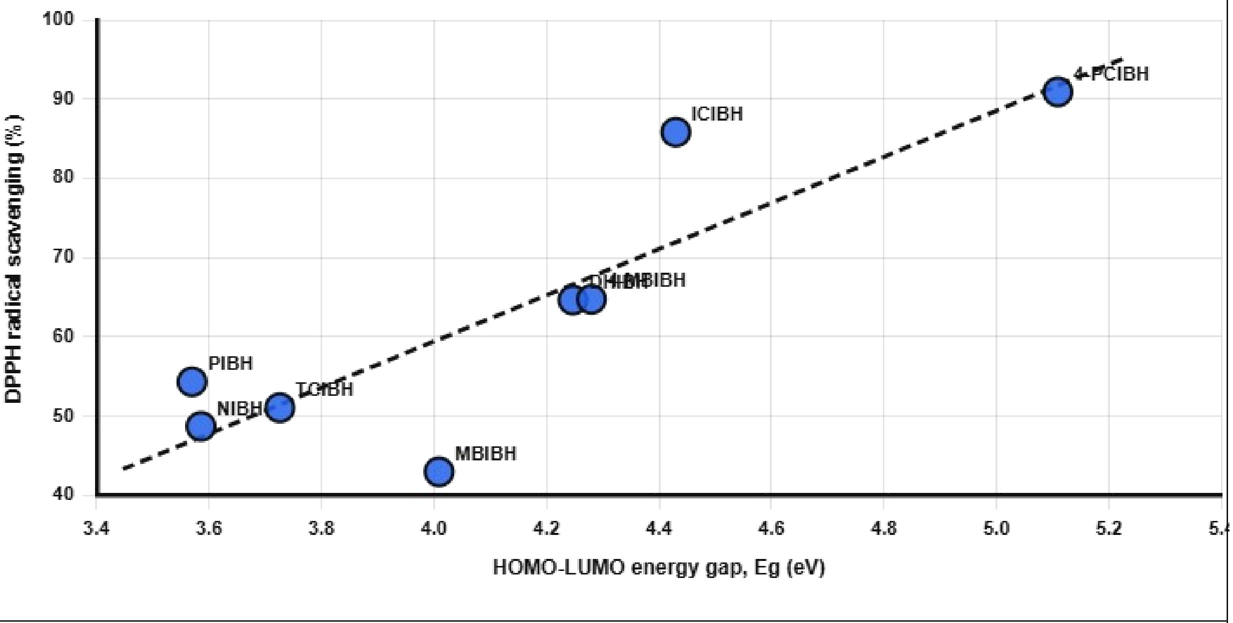

Good negative correlation. Compounds with smaller HOMO-LUMO gaps (PIBH, NIBH) are softer

electronically but 4-PCIBH dominates antioxidant activity due to its superior radical stabilization
**Correlation 2:** Electrophilicity index vs antibacterial activity (ZOI)<br>Higher electrophilicity → stronger interaction with nucleophilic bacterial enzyme residues<br>Regression equation: Y = 1.106X + (8.066) | R^2 = 0.028 | r = 0.167 | n = 8
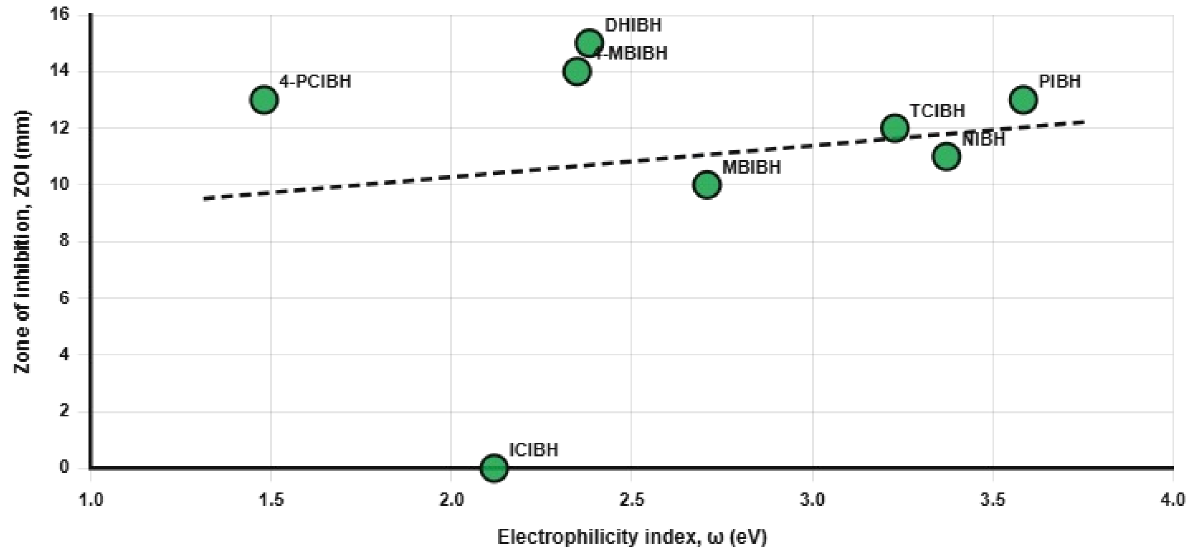
Moderate positive correlation. Higher electrophilicity associates with better antibacterial activity, supporting electrophilic attack on nucleophilic bacterial enzyme sites.
**Correlation 3:** Dipole moment vs DNA gyrase B docking score<br>Higher dipole moment → stronger interaction with polar active-site residues<br>**Regression equation:** Y = 0.024X + (-6.338) | R^2 = 0.008 | r = 0.091 | n = 8
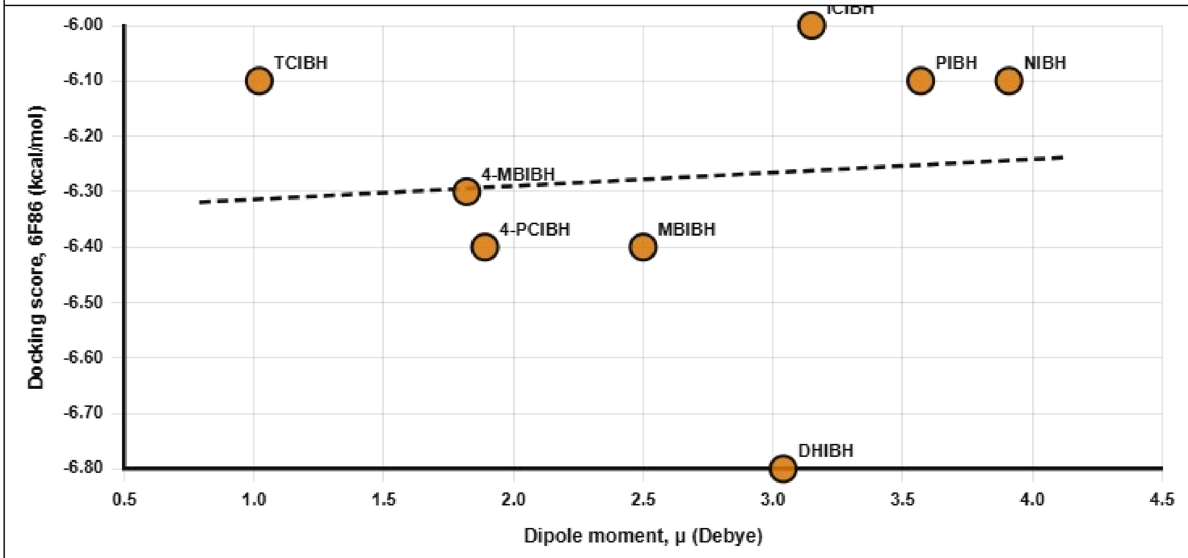
Moderate correlation. Higher dipole moments favor more negative (stronger) binding scores with the polar gyrase B

Antioxidant % Data (Control OD=0.539)

Triplicate 1	T1 %	Triplicate 2	T2 %	Triplicate 3	T3 %	% RSA Mean ± SE	IC_{50}
0.531	1.484230056	0.526	2.41187384	0.527	2.226345083	2.04±0.28	
0.533	1.113172542	0.52	3.525046382	0.512	5.009276438	3.22±1.14	
0.199	63.07977737	0.187	65.30612245	0.178	66.97588126	65.12±1.13	
0.08	85.15769944	0.084	84.41558442	0.082	84.78664193	84.79±0.21	44.51±7.46
0.394	26.90166976	0.398	26.15955473	0.391	27.45825603	26.84±0.38	
0.359	33.39517625	0.351	34.87940631	0.348	35.43599258	34.57±0.61	
0.303	43.78478664	0.307	43.04267161	0.301	44.15584416	43.66±0.33	
0.251	53.432282	0.255	52.69016698	0.258	52.13358071	52.75±0.38	81.16±0.67
0.398	26.15955473	0.402	25.4174397	0.397	26.34508349	25.97±0.28	
0.356	33.95176252	0.355	34.13729128	0.359	33.39517625	33.83±0.22	
0.305	43.41372913	0.309	42.6716141	0.306	43.22820037	43.1±0.22	
0.258	52.13358071	0.26	51.76252319	0.253	53.06122449	52.32±0.39	83.94±0.46
0.533	1.113172542	0.535	0.742115028	0.529	1.85528757	1.24±0.33	
0.452	16.14100186	0.451	16.32653061	0.44	18.36734694	16.94±0.71	
0.291	46.01113173	0.288	46.567718	0.3	44.34137291	45.64±0.67	
0.272	49.53617811	0.274	49.16512059	0.275	48.97959184	49.23±0.16	>100µL/mL
0.534	0.927643785	0.533	1.113172542	0.534	0.927643785	0.99±0.06	
0.532	1.298701299	0.532	1.298701299	0.529	1.85528757	1.48±0.19	>100µL/mL

0.53	1.669758813	0.527	2.226345083	0.526	2.41187384	2.10±0.22	
0.528	2.040816327	0.521	3.339517625	0.524	2.782931354	2.72±0.38	
0.48	10.94619666	0.482	10.57513915	0.487	9.647495362	10.39±0.39	
0.441	18.18181818	0.439	18.5528757	0.446	17.2541744	18±0.39	
0.351	34.87940631	0.348	35.43599258	0.352	34.69387755	35±0.22	
0.281	47.86641929	0.278	48.42300557	0.282	47.68089054	47.99±0.22	>100µL/mL
0.534	0.927643785	0.533	1.113172542	0.531	1.484230056	1.18±0.16	
0.533	1.113172542	0.527	2.226345083	0.528	2.040816327	1.79±0.34	
0.522	3.153988868	0.514	4.638218924	0.52	3.525046382	3.77±0.45	
0.51	5.380333952	0.503	6.67903525	0.501	7.050092764	6.37±0.51	>100µL/mL
0.53	1.669758813	0.531	1.484230056	0.534	0.927643785	1.36±0.22	
0.496	7.977736549	0.494	8.348794063	0.497	7.792207792	8.04±0.16	
0.451	16.32653061	0.453	15.9554731	0.454	15.76994434	16.02±0.16	
0.387	28.20037106	0.385	28.57142857	0.384	28.75695733	28.51±0.16	>100µL/mL
0.248	53.98886827	0.245	54.54545455	0.241	55.28756957	54.61±0.38	
0.152	71.79962894	0.153	71.61410019	0.156	71.05751391	71.49±0.22	
0.071	86.82745826	0.073	86.45640074	0.076	85.89981447	86.39±0.27	
0.02	96.28942486	0.023	95.73283859	0.027	94.99072356	95.67±0.38	11.07±1.55

Antioxidant

12.5µL/mL **25µL/mL** **50µL/mL** **100µL/mL**

**DHIBH**	2.040816327	3.215831787	65.12059369	84.78664193
**PIBH**	26.83982684	34.57019171	43.6611008	52.75200989
**NIBH**	25.97402597	33.82807669	43.10451453	52.31910946
**MBIBH**	1.23685838	16.9449598	45.64007421	49.22696351
**4-MBIBH**	0.989486704	1.484230056	2.102659246	2.721088435
**ICIBH**	10.38961039	17.99628942	35.00309215	47.99010513
**4-PCIBH**	1.175015461	1.793444651	3.772418058	6.369820656
**TCIBH**	1.360544218	8.039579468	16.01731602	28.50958565
**Ascorbic Acid**	54.60729746	71.49041435	86.39455782	95.67099567

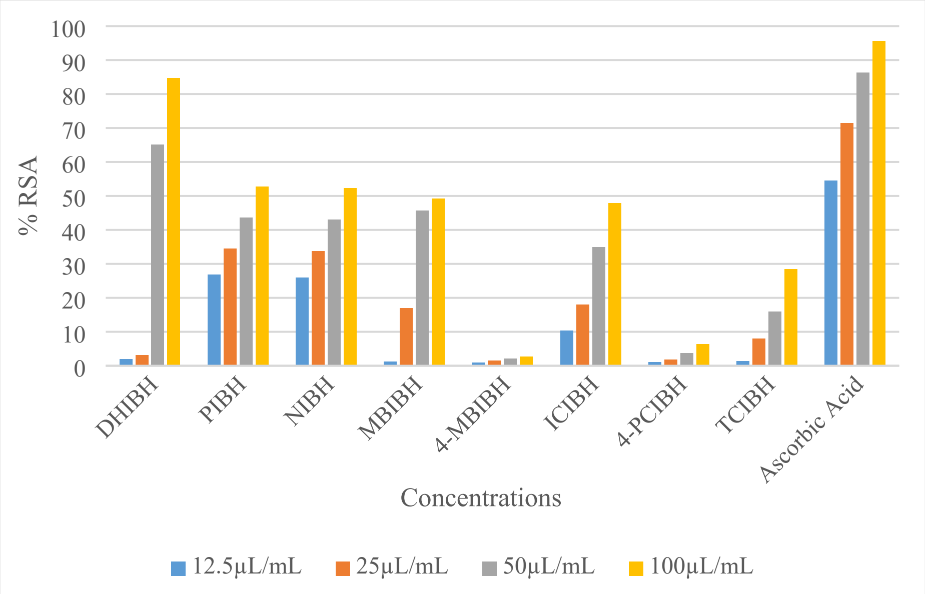

Anti-bacterial

E. coli

	Replicate 1 (mm)	Replicate 2 (mm)	Replicate 3 (mm)	Median ± SE (mm)
**Ciprofloxacin**	26.0	27.0	27.0	26.7±0.33
**DHIBH**	17.0	18.0	20.0	18.3±0.88
**PIBH**	16.0	15.0	16.0	15.7±0.33
**NIBH**	14.0	15.0	16.0	15.0±0.58
**MBIBH**	11.0	12.0	11.0	11.3±0.33
**4-MBIBH**	9.0	8.0	8.0	8.3±0.33
**ICIBH**	11.0	10.0	11.0	10.7±0.38
**4-PCIBH**	12.0	14.0	13.0	13.0±0.58
**TCIBH**	14.0	11.0	13.0	12.7±0.88

S. aureus

	Replicate 1 (mm)	Replicate 2 (mm)	Replicate 3 (mm)	Median ± SE (mm)
**Ciprofloxacin**	26.0	27.0	26.0	26.3±0.33
**DHIBH**	19.0	20.0	19.0	19.3±0.33
**PIBH**	10.0	11.0	9.0	10.0±0.58
**NIBH**	9.0	8.0	9.0	8.7±0.33
**MBIBH**	0.0	0.0	0.0	0.0
**4-MBIBH**	8.0	9.0	8.0	8.3±0.33
**ICIBH**	10.0	10.0	9.0	9.7±0.38
**4-PCIBH**	12.0	11.0	13.0	12.0±0.58
**TCIBH**	11.0	10.0	11.0	10.7±0.33
